# Supplementary material for: Can prognostic factors for indirect muscle injuries in elite football (soccer) players be identified using data from preseason screening? An exploratory analysis using routinely collected periodic health examination records
Source: BMJ Open. 2023 Jan 24;13(1):e052772. doi: 10.1136/bmjopen-2021-052772 (PMC9884927; doi:10.1136/bmjopen-2021-052772)
Supplement: Supplementary data [file bmjopen-2021-052772supp001.pdf]

Does preseason screening provide a source of potential prognostic factors for indirect muscle injuries in elite football (soccer) players? An exploratory analysis using routinely-collected periodic health examination data

Hughes, T., Riley, R.D., Callaghan, M.J. and Sergeant, J.C. (2022)

Supplementary file 1: Detailed descriptions of all PHE tests.

Anthropometric measurements

For each participant, height was recorded in centimetres (cm) and body weight was measured in kilograms (kg) using a combined weight and height scales (Seca, Hamburg, Germany). Body mass index was calculated as weight (kg) divided by the squared height (metres). Body compositions were estimated through percentage body fat calculations; using a generic skinfold calliper, measurements were taken from the of triceps, subscapular, lower abdominal, anterior thigh, pectoral and iliac crest anatomical locations by the same examiner.

Past Medical/Injury history

All participants underwent an interview with a club medical doctor to discuss their past medical and injury history. For participants who were transferred to the club during the data collection period, their initial PHE included questions regarding family medical history and any previous significant injuries sustained at their previous club (which resulted in time lost to training of > 7-10 days). This information was cross referenced with their medical records received from the previous football club. Subsequent PHEs consisted of an interview which reviewed the medical information and injury history that was routinely recorded on the medical notes system during each participant's employment with the club.

Musculoskeletal examination

Participants underwent a series of musculoskeletal tests that were completed by one examiner, while another examiner monitored the procedure for any accessory or compensatory movements and verified the measurement before recording it into a database. If any accessory or compensatory movements were noted the procedure for that particular test was repeated. All examination procedures were performed according to a standardised protocol.

Passive Hip Internal/External Rotation

Hip rotational range of movement was used to evaluate hip joint mobility.(1) Participants laid supine with both thighs supported by the treatment plinth and knees flexed over the end of the plinth to allow the lower legs to hang freely. A digital inclinometer (Digi-Pas Uk – Tarax Technology Ltd, Cambridge) was placed on the medial aspect of the tibia immediately proximal to the medial malleolus of the tibia. The examiner instructed the participant to flex the contralateral hip and knee and place the foot on the plinth to stabilise the pelvis. The hip was passively rotated to the maximal range of external rotation, until resistance prevented any further movement or symptoms were experienced. The range was documented in degrees and entered into the database. The test was repeated for passive internal rotation. Both procedures were repeated on the contralateral limb. Use of a digital inclinometer to evaluate hip rotation has previously been shown to have excellent intra-rater (intraclass correlation coefficient (ICC)= 0.84- 0.90)(1, 2), inter-rater (ICC=0.89-0.93)(3) and test-retest reliability (ICC=0.93-0.96).(4)

Modified Thomas Test

The modified Thomas test was used to measure the flexibility of the anterior hip musculature.(5) Participants stood against a plinth which was adjusted so it was aligned to the height of the anterior superior iliac spine (ASIS). Participants lay supine on the edge of the plinth with both legs hanging freely. Both hips and knees were maximally flexed and held to the chest with both hands of the participant. One limb was released and the examiner extended the hip with as far as possible, whilst ensuring that the lumbar spine remained in contact with the plinth and the contralateral knee maintained the flexed position. A digital inclinometer (Digi-Pas Uk – Tarax Technology Ltd, Cambridge) was placed on the muscle belly of the upper 1/3 of the quadriceps and the measurement was recorded in degrees. If the femur was angled so it was above horizontal, a negative measurement was recorded. If it was below the horizontal, a positive measure was recorded. The procedure was then repeated on the contralateral leg. The inter-rater reliability of using an inclinometer with this test has be previously established as good (ICC= 0.89).(6)

Straight Leg Raise

The straight leg raise (SLR) was used to measure of hamstring and neurodynamic mobility.(5) The participant laid supine on a plinth. A digital inclinometer (Digi-Pas Uk – Tarax Technology Ltd, Cambridge) was placed on the anterior margin of the tibia (10 centimetres distal to the tibial plateau) by the examiner. The examiner maintained full knee extension and passively flexed the hip to its maximal range in the sagittal plane, or to the point where any symptoms were noted. The range was recorded in degrees and the test was repeated on the contralateral limb. Previously for this method of measurement, intra-rater reliability has been shown to be excellent (ICC 0.95-0.98) (7) and inter-rater reliability has been shown to be good (ICC 0.80-0.97). (8)

Weight Bearing Lunge

The weight bearing lunge (WBL) was used to measure dorsiflexion mobility of the ankle.(9) Participants were instructed to stand along a tape line which was perpendicular to the wall, where the second toe and centre of the heel were placed over the line. A digital inclinometer (Digi-Pas Uk – Tarax Technology Ltd, Cambridge) was placed over the anterior margin of the tibia (10 centimetres distal to the tibial plateau) in a neutral position. Participants were instructed to step as far forward as possible with the contralateral leg, into a forward stride position with both hands on the wall in front, which dorsiflexed the rear ankle. Participants were instructed to lean forward as much as possible until resistance or symptoms were experienced, whilst maintaining knee extension with contact of the heel on the floor. If the heel lifted from the ground the test was deemed as invalid and then repeated. The maximal range of dorsiflexion was documented in degrees. The test was repeated on the contralateral leg. Previously, this method has been shown to have good intra-rater reliability (ICC=0.88) and good to excellent inter-rater reliability (ICC 0.80- 0.95.(10, 11)

Strength and Power Tests

Maximal Loaded Leg Press

This test was used to measure the force, power and velocity for both lower limbs during an incremental leg double press test, which progressed from minimal to maximal resistance,

using a Keiser Air 300 machine (Keiser UK, Tetbury, Gloucestershire). Prior to this test participants warmed up on a Keiser M3 exercise cycle (Keiser UK, Tetbury, Gloucestershire) for 5 mins at resistance level one. Participants sat on the leg press machine and an appropriate seat position was selected, with the feet firmly rested on the footplates and the knees were at 90 degrees of flexion. Participants were instructed to generate as much force as possible, as quickly as possible by pushing against the footplate and keeping the spine flat against the seat during completion of the test movement. A test was deemed invalid if any compensatory movements were observed (such as elevating the pelvis from the seat to gain a mechanical advantage) and the test was repeated. The test consisted of repeated single repetitions, with each repetition increasing in resistance until the maximum resistance was reached and the participant could not complete the leg press movement. The measurements obtained for this test are peak power (Watts/kg(W/kg)), Peak velocity (metres/second (m/s)) and peak force (Newtons/kg (N/kg)) for each limb. The test-retest reliability has been shown to have good-excellent reliability for the power (ICC= 0.89), velocity (ICC= 0.79) and force (ICC=0.91) parameters. (12)

Countermovement jump (CMJ)

This test provided a functional measure of explosive power.(13, 14) All CMJs were performed on a dual plate force platform system (ForceDecks FD4000, ForceDecks, Salford, UK) and analysed using the manufacturer's dedicated software. The test procedure was explained and demonstrated to each participant. Participants were instructed to stand on the platform, with foot placement approximately hip width apart and their hands placed on the pelvis for the duration of the CMJ. In one dynamic movement, participants squatted to approximately 90 degrees of knee flexion before forcefully propelling vertically into the jump phase of the movement through triple extension of hips, knees and ankles, landing approximately in the original start position. Four CMJ repetitions were completed, although if jump height progressively increased with each effort, further CMJs were completed until height scores plateaued. If participants hands shifted position from the pelvis or the legs did not remain in an extended position during the take-off or flight phase then the repetition was deemed invalid and the test was repeated. The measurements taken from this test were peak jump height (cm), peak power (watts) and force per kg of body mass (N/kg). The test-retest reliability of peak jump height has been previously shown to be good (ICC=0.80-0.88).(15)

Similarly, for CMJ power, test-retest reliability has been shown to be excellent (ICC=0.92-0.98)(16).

References

1. Roach S, San Juan JG, Suprak DN, Lyda MA. Concurrent validity of digital inclinometer and universal goniometer assessing passive hip mobility in healthy subjects. The Int J Sport Phys Ther. 2013;8(5):680-8.

2. Krause DA, Hollman JH, Krych AJ, Kalisvaart MM, Levy BA. Reliability of hip internal rotation range of motion measurement using a digital inclinometer. Knee Surg Sports Traumatol Arthrosc. 2015;23(9):2562-7.

3. Hollman JH, Ginos BE, Kozuchowski J, Vaughn AS, Krause D, Youdas J. Relationships Between Knee Valgus, Hip-Muscle Strength, and Hip-Muscle Recruitment During a Single-Limb Step-Down. J Sport Rehab. 2009;18:104-17.

4. Pua YH, Wrigley TV, Cowan SM, Bennell KL. Intrarater test-retest reliability of hip range of motion and hip muscle strength measurements in persons with hip osteoarthritis. Arch Phys Med Rehabil. 2008;89(6):1146-54.

5. Magee DJ. Orthopedic Physical Assessment. 5th Ed. ed. Missouri: Saunders Elsevier; 2008.

6. Clapis PA, Davis SM, Davis RO. Reliability of inclinometer and goniometric measurements of hip extension flexibility using the modified Thomas test. Phys Theory Practice. 2008;24(2):135-41.

7. Boyd BS. Measurement properties of a hand-held inclinometer during straight leg raise neurodynamic testing. Physiotherapy. 2012;98(2):174-9.

8. Gabbe BJ, Bennell KL, Wajswelner H, Finch CF. Reliability of common lower extremity musculoskeletal screening tests. Phys Ther Sport. 2004;5(2):90-7.

9. Catalatayud J, Martin F, Gargallo P, Garcia-Redondo J, Colado JC, Marin PJ. The validity and reliability of a new instrumented device for measuring ankle dorsiflexion range of motion. Int J Sports Phys Ther. 2015;10(2):197-202.

10. Williams CM, Caserta AJ, Haines TP. The TiltMeter app is a novel and accurate measurement tool for the weight bearing lunge test. J Sci Med Sport. 2013;16(5):392-5.

11. Munteanu SE, Strawhorn AB, Landorf KB, Bird AR, Murley GS. A weightbearing technique for the measurement of ankle joint dorsiflexion with the knee extended is reliable. J Sci Med Sport. 2009;12(1):54-9.

12. Redden J, Stokes K, Williams S. Establishing the Reliability and Limits of Meaningful Change of Lower Limb Strength and Power Measures during Seated Leg Press in Elite Soccer Players. J Sports Sci Med. 2018;17:539-46.

13. Reiser RF, Rocheford EC, Armstrong CJ. Building a better understanding of basic mechanical prinicples through analysis of the vertical jump. Strength Cond J (Lippincott Williams & Wilkins). 2006;28(4):70-80.

14. Maulder P, Cronin J. Horizontal and vertical jump assessment: reliability, symmetry, discriminative and predictive ability. Phys Ther Sport. 2005;6(2):74-82.

15. Slinde F, Suber C, Suber L, Edwen CE, Svantesson U. Test–Retest Reliability Of Three Different Countermovement Jumping Tests. J Strength Cond Res. 2008;22(2):640-3.

16. Hori N, Newton RU, Kawamori N, McGuigan MR, Kraemer WJ, Nosaka K. Reliability of performance measurements derived from ground reaction force data during countermovement jump and the influence of sampling frequency. J Strength Cond Res (Allen Press Publishing Services Inc). 2009;23(3):874-82.
