# Supplementary material for: Can prognostic factors for indirect muscle injuries in elite football (soccer) players be identified using data from preseason screening? An exploratory analysis using routinely collected periodic health examination records
Source: BMJ Open. 2023 Jan 24;13(1):e052772. doi: 10.1136/bmjopen-2021-052772 (PMC9884927; doi:10.1136/bmjopen-2021-052772)
Supplement: Supplementary data [file bmjopen-2021-052772supp002.pdf]

Does preseason screening provide a source of potential prognostic factors for indirect muscle injuries in elite football (soccer) players? An exploratory analysis using routinely-collected periodic health examination data

Hughes, T., Riley, R.D., Callaghan, M.J. and Sergeant, J.C. (2022)

Supplementary file 2: Sample size calculation

Tests for the Odds Ratio in Logistic Regression with One Normal X and Other Xs (Wald Test)

Numeric Results

Power	N	P0	P1	Odds Ratio	R Squared	Alpha	Beta
0.79904	314	0.430	0.542	1.570	0.500	0.050	0.20096

References

Hsieh, F.Y., Block, D.A., and Larsen, M.D. 1998. 'A Simple Method of Sample Size Calculation for Linear and
Logistic Regression', Statistics in Medicine, Volume 17, pages 1623-1634.

Report Definitions

Power is the probability of rejecting a false null hypothesis. It should be close to one.
N is the size of the sample drawn from the population.
P0 is the response probability at the mean of X.
P1 is the response probability when X is increased to one standard deviation above the mean.
Odds Ratio is the odds ratio when P1 is on top. That is, it is [P1/(1-P1)]/[P0/(1-P0)].
R-Squared is the R2 achieved when X is regressed on the other independent variables in the regression.
Alpha is the probability of rejecting a true null hypothesis.
Beta is the probability of accepting a false null hypothesis.

Summary Statements

A logistic regression of a binary response variable (Y) on a continuous, normally distributed variable (X) with a sample size of 314 observations achieves 80% power at a 0.050 significance level to detect a change in Prob(Y=1) from the value of 0.430 at the mean of X to 0.542 when X is increased to one standard deviation above the mean. This change corresponds to an odds ratio of 1.570. An adjustment was made since a multiple regression of the independent variable of interest on the other independent variables in the logistic regression obtained an R-Squared of 0.500.

Dropout-Inflated Sample Size

Dropout Rate	Sample Size N	Dropout-Inflated Enrollment Sample Size N'	Expected Number of Dropouts D
20%	314	393	79

Definitions

Dropout Rate (DR) is the percentage of subjects (or items) that are expected to be lost at random during the
course of the study and for whom no response data will be collected (i.e. will be treated as "missing").
N is the evaluable sample size at which power is computed. If N subjects are evaluated out of the N' subjects
that are enrolled in the study, the design will achieve the stated power.
N' is the total number of subjects that should be enrolled in the study in order to end up with N

evaluable
subjects, based on the assumed dropout rate. After solving for N, N' is calculated by inflating N using the
formula N' = N / (1 - DR), with N' always rounded up. (See Julious, S.A. (2010) pages 52-53, or Chow, S.C.,
Shao, J., Wang, H., and Lokhnygina, Y. (2018) pages 32-33.)
D is the expected number of dropouts. D = N' - N.

Tests for the Odds Ratio in Logistic Regression with One Normal X and Other Xs (Wald Test)

Procedure Input Settings

Autosave Inactive

Design Tab

Solve For:	Sample Size
Alternative Hypothesis:	Two-Sided
Power:	0.80
Alpha:	0.05
P0 (Baseline Probability that Y=1):	0.43
Use P1 or Odds Ratio:	Odds Ratio
Odds Ratio (Odds1/Odds0):	1.57
R-Squared of X1 with Other X's:	0.5
