# Supplementary material for: Can prognostic factors for indirect muscle injuries in elite football (soccer) players be identified using data from preseason screening? An exploratory analysis using routinely collected periodic health examination records
Source: BMJ Open. 2023 Jan 24;13(1):e052772. doi: 10.1136/bmjopen-2021-052772 (PMC9884927; doi:10.1136/bmjopen-2021-052772)
Supplement: Supplementary data [file bmjopen-2021-052772supp003.pdf]

BMJ Publishing Group Limited (BMJ) disclaims all liability and responsibility arising from any reliance placed on this supplemental material which has been supplied by the author(s)

Does preseason screening provide a source of potential prognostic factors for indirect muscle injuries in elite football (soccer) players? An exploratory analysis using routinely-collected periodic health examination data

Hughes, T., Riley, R.D., Callaghan, M.J. and Sergeant, J.C. (2022)

Supplementary file 3: Candidate prognostic factors that were excluded

Type of prognostic factor	Candidate Prognostic Factor	Composite variable created	Measurement unit	Measurement method	Data type	Reason for elimination
Anthropometric	Body fat	No	Percentage	Skin calipers	Continuous	Missing data > 15%
Musculoskeletal test	Quadriceps muscle length difference*	Yes	Degrees	Ely's test using digital inclinometer	Continuous	Intra-rater ICC = 0.69[1] Inter-rater ICC = 0.66. [1]
	Mean quadriceps muscle length**	Yes	Degrees	Ely's test using goniometer	Continuous	Intra-rater ICC = 0.69[1] Inter-rater ICC = 0.66. [1]
	Toe touch in standing	No	Centimetres	Fingertips to floor distance	Continuous	Missing data > 15%
	Sacroiliac joint kinematic function	No	Subjective score	Gillets test	Categorical	Missing data > 15%
Functional movement/balance tests	Y Balance Test – anterior translation difference*	Yes	Centimetres	Y Balance Test	Continuous	Missing data >15%
	Y Balance Test – Mean anterior translation**	Yes	Centimetres	Y Balance Test	Continuous	Missing data >15%
	Y Balance Test – posteromedial translation difference*	Yes	Centimetres	Y Balance Test	Continuous	Missing data >15%
	Y Balance Test – Mean posteromedial translation**	Yes	Centimetres	Y Balance Test	Continuous	Missing data >15%
	Y Balance Test –posterolateral translation difference*	Yes	Centimetres	Y Balance Test	Continuous	Missing data >15%
	Y Balance Test – Mean posterolateral translation**	Yes	Centimetres	Y Balance Test	Continuous	Missing data >15%
	R relative tibial angles	No	Degrees	SLS measurement with Dorsavi Viperform IMU	Continuous	Within-session ICCs=0.27-0.75<br><br>Between-session ICCs = 0.55-0.77[2]
	L relative tibial angles (left leg)	No	Degrees	SLS measurement with Dorsavi Viperform IMU	Continuous	Within-session ICCs= 0.27-0.75<br><br>Between-session ICCs = 0.55-0.77[2]
Strength/power tests	Upper body peak power difference*	Yes	Normalised watts per kilo ($W/kg^{-0.67}$)	Double horizontal press using a Keiser Chest Press Air 350 machine	Continuous	Missing data > 15%
	Mean upper body peak power**	Yes	Normalised watts per kilo ($W/kg^{-0.67}$)	Double horizontal press using a Keiser Chest Press Air 350 machine	Continuous	Missing data > 15%

*Key: PHE=periodic health examination; WBL=weight bearing lunge; CMJ=countermovement jump; PROM=passive range of movement; ICC=intraclass correlation coefficient; SLR= straight leg raise; SLS = single leg squat; BMI= body mass index; f=force; W= watts; (note that $W/kg^{-0.67}$ has a scaling factor to normalise power to body mass) Kg=kilos; IMU= inertial measurement units; m = mass; Note: composite factors are identified in the table with * denoting between limb differences and **denoting combined mean values of both limbs.*

References:

1. Peeler J, Anderson JE. Reliability of the Ely's test for assessing rectus femoris muscle flexibility and joint range of motion. *J Orth Res* 2008;26(6):793-9.

2. Hughes T, Jones RK, Starbuck C, et al. Are tibial angles measured with inertial sensors useful surrogates for frontal plane projection angles measured using 2-dimensional video analysis during single leg squat tasks? A reliability and agreement study in elite football (soccer) players. *J Electromyogr Kinesiol* 2019;44:21-30. doi: 10.1016/j.jelekin.2018.11.005
