# Supplementary material for: Can prognostic factors for indirect muscle injuries in elite football (soccer) players be identified using data from preseason screening? An exploratory analysis using routinely collected periodic health examination records
Source: BMJ Open. 2023 Jan 24;13(1):e052772. doi: 10.1136/bmjopen-2021-052772 (PMC9884927; doi:10.1136/bmjopen-2021-052772)
Supplement: Supplementary data [file bmjopen-2021-052772supp006.pdf]

Does preseason screening provide a source of potential prognostic factors for indirect muscle injuries in elite football (soccer) players? An exploratory analysis using routinely-collected periodic health examination data

Hughes, T., Riley, R.D., Callaghan, M.J. and Sergeant, J.C. (2022)

Supplementary file 6: Results of univariable and multivariable analyses – Primary complete case analysis

	Univariable (unadjusted)				Multivariable (adjusted for age, height, weight)			
**Candidate PF & Type**	OR	95% CI	P	Best model fit	OR	95% CI	P	Best model fit
**Anthropometric (Adjustment Factors):**								
1: Age (years)	**1.12**	**1.06 to 1.18**	**<0.001**	**Linear**	**1.11**	**1.04 to 1.18**	**<0.001**	**Linear**
2: Height (cm)	1.03	0.99 to 1.06	0.18	-	1.02	0.97 to 1.07	0.50	-
3: Weight (kg)	1.03	0.99 to 1.06	0.07	-	0.99	0.95 to 1.04	0.81	-
**Within 3 years preceding PHE, frequency of:**								
4: foot/ankle injuries	1.04	0.87 to 1.23	0.68	-	1.04	0.86 to 1.25	0.70	-
5: hip/groin injuries	1.16	0.90 to 1.51	0.25	-	1.29	0.98 to 1.70	0.07	-
6: knee injuries	0.96	0.72 to 1.29	0.81	-	1.00	0.74 to 1.35	0.98	-
7: shoulder injuries	2.38	0.98 to 5.75	0.05	-	1.77	0.68 to 4.59	0.24	-
8: lumbar spine injuries	0.88	0.60 to 1.29	0.50	-	1.03	0.68 to 1.55	0.89	-
9: iliopsoas IMIs	0.73	0.38 to 1.43	0.37	-	0.88	0.44 to 1.76	0.72	-
10: hip adductor IMIs	1.38	0.92 to 2.09	0.12	-	1.22	0.78 to 1.90	0.39	-
11: hamstring IMIs	**1.56**	**1.17 to 2.09**	**<0.001**	**Linear**	1.30	0.96 to 1.77	0.09	-
12: quadriceps IMIs	1.08	0.67 to 1.73	0.75	-	1.00	0.61 to 1.63	0.99	-
13: calf IMIs	**1.80**	**1.09 to 2.97**	**0.02**	**Linear**	1.30	0.74 to 2.29	0.35	-
**Within 3 years preceding PHE, most recent:**								
14: foot/ankle injury (never)	ref	ref	ref	-	ref	ref	ref	-
14: foot/ankle injury (<6 months)	1.27	0.64 to 2.53	0.49	-	1.36	0.66 to 2.79	0.40	-
14: foot/ankle injury (6-12 months)	1.16	0.54 to 2.46	0.71	-	1.09	0.49 to 2.45	0.83	-
14: foot/ankle injury (>12 months)	1.27	0.75 to 2.13	0.37	-	1.05	0.60 to 1.84	0.86	-
15: hip/groin injury (never)	ref	ref	ref	-	ref	ref	ref	-
15: hip/groin injury (<6 months)	1.05	0.44 to 2.49	0.92	-	1.45	0.52 to 4.00	0.48	-
15: hip/groin injury (6-12 months)	0.59	0.23 to 1.50	0.27	-	1.17	0.35 to 3.84	0.80	-
15: hip/groin injury (>12months)	1.58	0.86 to 2.87	0.14	-	1.14	0.45 to 2.88	0.78	-
16: knee injury (never)	ref	ref	ref	-	ref	ref	ref	-
16: knee injury (<6 months)	1.15	0.40 to 3.28	0.80	-	1.11	0.33 to 3.72	0.87	-
16: knee injury (6-12 months)	1.23	0.58 to 2.62	0.60	-	1.24	0.57 to 2.72	0.59	-
16: knee injury (>12months)	0.93	0.53 to 1.60	0.79	-	1.10	0.61 to 1.98	0.76	-
17: shoulder injury (never)	ref	ref	ref	-	ref	ref	ref	-
17: shoulder injury (<6 months)	2.75	0.50 to 15.26	0.25	-	2.07	0.33 to 12.90	0.44	-
17: shoulder injury (6-12 months)	1.38	0.19 to 9.90	0.75	-	1.12	0.15 to 8.37	0.91	-
17: shoulder injury (>12months)	3.21	0.81 to 12.66	0.10	-	2.38	0.59 to 9.70	0.23	-
18: lumbar spine injury (never)	ref	ref	ref	-	ref	ref	ref	-
18: lumbar spine injury (<6 months)	1.24	0.30 to 5.05	0.77	-	2.66	0.57 to 12.51	0.22	-
18: lumbar spine injury (6-12 months)	0.62	0.15 to 2.52	0.50	-	0.72	0.17 to 3.03	0.66	-
18: lumbar spine injury (>12months)	0.70	0.34 to 1.44	0.33	-	0.90	0.42 to 1.94	0.79	-
19: iliopsoas IMI (never)	ref	ref	ref	-	ref	ref	ref	-
19: iliopsoas IMI (<6 months)	1.24	0.08 to 20.05	0.88	-	1.29	0.08 to 21.58	0.86	-
19: iliopsoas IMI (6-12 months)	0.62	0.15 to 2.53	0.51	-	0.80	0.19 to 3.33	0.76	-
19: iliopsoas IMI (>12months)	0.57	0.21 to 1.60	0.27	-	0.70	0.25 to 1.96	0.50	-
20: hip adductor IMI (never)	ref	ref	ref	-	ref	ref	ref	-
20: hip adductor IMI (<6 months)	1.37	0.53 to 3.56	0.52	-	1.45	0.52 to 4.00	0.48	-
20: hip adductor IMI (6-12 months)	1.37	0.43 to 4.36	0.60	-	1.17	0.35 to 3.84	0.80	-
20: hip adductor IMI (>12months)	1.37	0.59 to 3.16	0.46	-	1.14	0.45 to 2.88	0.78	-
21: hamstring IMI (never)	ref	ref	ref	-	ref	ref	ref	-
21: hamstring IMI (<6 months)	2.84	0.81 to 9.99	0.10	-	1.44	0.36 to 5.77	0.60	-
21: hamstring IMI (6-12 months)	1.42	0.66 to 3.06	0.37	-	1.25	0.55 to 2.86	0.60	-
21: hamstring IMI (>12months)	**2.95**	**1.51 to 5.73**	**<0.001**	**Linear**	2.10	1.01 to 4.34	0.05	-
22: quadriceps IMI (never)	ref	ref	ref	-	ref	ref	ref	-
22: quadriceps IMI (<6 months)	1.74	0.38 to 7.91	0.48	-	1.52	0.32 to 7.20	0.60	-
22: quadriceps IMI (6-12 months)	0.58	0.17 to 1.93	0.37	-	0.72	0.21 to 2.50	0.60	-
22: quadriceps IMI (>12months)	1.14	0.53 to 2.42	0.74	-	0.85	0.37 to 1.94	0.69	-
23: calf IMI (never)	ref	ref	ref	-	ref	ref	ref	-
23: calf IMI (<6 months)	3.78	0.98 to 14.56	0.05	-	2.80	0.68 to 11.47	0.15	-
23: calf IMI (6-12 months)	7.09	0.82 to 61.51	0.08	-	3.36	0.35 to 32.71	0.30	-
23: calf IMI (>12months)	1.26	0.56 to 0.89	0.64	-	0.87	0.29 to 2.62	0.80	-
**Musculoskeletal:**								
24. Mean PROM hip IR (deg.)	**0.97**	**0.95 to 0.99**	**0.01**	**Linear**	**0.97**	**0.95 to 1.00**	**0.04**	**Linear**
25. Mean PROM hip ER (deg.)	0.97	0.94 to 1.00	0.08	-	0.98	0.95 to 1.01	0.26	-
26. Mean hip flexor length (deg.)	1.01	0.98 to 1.04	0.50	-	1.01	0.98 to 1.04	0.50	-

27. Mean hamstring/neural mobility length (deg.)	0.99	0.96 to 1.02	0.53	-	0.98	0.94 to 1.01	0.21	-
28. Mean calf muscle length (deg.)	1.00	0.97 to 1.02	0.73	-	0.99	0.97 to 1.02	0.64	-
**Strength/Power:**								
29: Max. leg extension power difference (W/kg$^{-0.67}$)	1.00	0.93 to 1.07	0.92	-	0.99	0.92 to 1.07	0.78	-
30: Mean of max. leg extension power (W/kg$^{-0.67}$)	1.02	0.99 to 1.05	0.30	-	1.01	0.97 to 1.04	0.76	-
31: Max. leg extension velocity difference (m.s^{-1})	2.14	0.30 to 15.00	0.45	-	2.61	0.34 to 19.87	0.36	-
32: Mean of max. leg extension velocity (m.s^{-1})	1.86	0.63 to 5.53	0.26	-	1.44	0.46 to 4.53	0.53	-
33: Max leg extension force difference (N/kg$^{-0.67}$)	1.00	0.98 to 1.02	0.66	-	1.00	0.98 to 1.02	0.67	-
34: Mean of max. leg extension force (N/kg$^{-0.67}$)	1.00	0.99 to 1.01	0.96	-	1.00	0.98 to 1.01	0.69	-
35: CMJ Force per kg of body mass (N/kg)	0.99	0.92 to 1.06	0.73	-	0.99	0.92 to 1.06	0.82	-
36: CMJ height (cm)	1.03	0.97 to 1.07	0.34	-	1.01	0.96 to 1.07	0.59	-

Key: PHE= periodic health examination; PF= prognostic factor; OR=odds ratio; CI=confidence interval; ref=reference category; I-IMI=index indirect muscle injury; IMI= indirect muscle injury; Freq= frequency; WBL=weight bearing lunge; CMJ=countermovement jump; PROM=passive range of movement; deg. = degrees; SLR= straight leg raise; BMI= body mass index; kg/m^2 = kilograms/body height (metres) squared; *cm = centimetres; Kg=kilograms; W= watts (note: W/kg$^{-0.67}$ has a scaling factor to normalise force to body mass[42]); N= newtons (note: N/kg$^{-0.67}$ has a scaling factor to normalise force to body mass);max.=maximum; m.s.= metres per second; cm = centimetres; Kg=kilograms; - = not applicable.*
Note: *ORs are expressed per one-unit increase for all continuous factors, and according to category for all categorical factors; Factors in* ***bold*** *indicate significance at the 0.05 level.*
