# Supplementary material for: Can prognostic factors for indirect muscle injuries in elite football (soccer) players be identified using data from preseason screening? An exploratory analysis using routinely collected periodic health examination records
Source: BMJ Open. 2023 Jan 24;13(1):e052772. doi: 10.1136/bmjopen-2021-052772 (PMC9884927; doi:10.1136/bmjopen-2021-052772)
Supplement: Supplementary data [file bmjopen-2021-052772supp007.pdf]

Does preseason screening provide a source of potential prognostic factors for indirect muscle injuries in elite football (soccer) players? An exploratory analysis using routinely-collected periodic health examination data

Hughes, T., Riley, R.D., Callaghan, M.J. and Sergeant, J.C. (2022)

Supplementary file 7: Results of univariable and multivariable analyses – Sensitivity analysis using imputed data

	Univariable (unadjusted)				Multivariable (adjusted for age, height, weight)			
**Candidate PF & Type**	OR	95% CI	P	Best model fit	OR	95% CI	P	Best model fit
**Anthropometric (Adjustment Factors):**								
1: Age (years)	**1.15**	1.08 to 1.22	**<0.001**	**Linear**	**1.14**	**1.06 to 1.22**	**<0.001**	**Linear**
2: Height (cm)	1.02	0.98 to 1.06	0.25	-	0.99	0.94 to 1.05	0.85	-
3: Weight (kg)	**1.05**	**1.02 to 1.08**	**0.01**	**Linear**	1.02	0.97 to 1.07	0.49	-
**Within 3 years preceding PHE, frequency of:**								
4: foot/ankle injuries	1.03	0.86 to 1.25	0.73	-	1.04	0.85 to 1.27	0.70	-
5: hip/groin injuries	1.09	0.83 to 1.43	0.54	-	1.23	0.92 to 1.64	0.16	-
6: knee injuries	0.91	0.67 to 1.23	0.54	-	0.92	0.67 to 1.26	0.59	-
7: shoulder injuries	1.91	0.80 to 4.59	0.15	-	1.34	0.53 to 3.39	0.54	-
8: lumbar spine injuries	0.78	0.53 to 1.16	0.22	-	0.99	0.66 to1.50	0.98	-
9: iliopsoas IMIs	0.74	0.36 to 1.50	0.40	-	0.86	0.41 to 1.81	0.70	-
10: hip adductor IMIs	1.46	0.93 to 2.30	0.10	-	1.14	0.70 to 1.87	0.60	-
11: hamstring IMIs	**2.39**	**1.55 to 3.69**	**<0.001**	**Linear**	**1.87**	**1.19 to 2.96**	**0.01**	**Linear**
12: quadriceps IMIs	1.12	0.67 to 1.86	0.66	-	1.09	0.64 to 1.87	0.75	-
13: calf IMIs	**2.17**	**1.13 to 4.16**	**0.02**	**Linear**	1.47	0.70 to 3.08	0.31	-
**Within 3 years preceding PHE, most recent:**								
14: foot/ankle injury (never)	ref	ref	ref	-	ref	ref	ref	-
14: foot/ankle injury (<6 months)	1.25	0.59 to 2.61	0.56	-	1.52	0.70 to 3.31	0.29	-
14: foot/ankle injury (6-12 months)	1.27	0.57 to 2.85	0.57	-	1.30	0.55 to 3.09	0.55	-
14: foot/ankle injury (>12 months)	1.39	0.78 to 2.49	0.27	-	1.26	0.67 to 2.34	0.47	-
15: hip/groin injury (never)	ref	ref	ref	-	ref	ref	ref	-
15: hip/groin injury (<6 months)	0.77	0.31 to 1.91	0.57	-	1.36	0.52 to 3.52	0.53	-
15: hip/groin injury (6-12 months)	0.55	0.21 to 1.45	0.23	-	0.57	0.20 to 1.58	0.28	-
15: hip/groin injury (>12months)	1.56	0.79 to 3.09	0.20	-	1.87	0.92 to 3.81	0.09	-
16: knee injury (never)	ref	ref	ref	-	ref	ref	ref	-
16: knee injury (<6 months)	0.89	0.31 to 2.55	0.82	-	0.87	0.28 to 2.67	0.81	-
16: knee injury (6-12 months)	1.20	0.51 to 2.82	0.68	-	1.15	0.47 to 2.83	0.76	-
16: knee injury (>12months)	0.94	0.51 to 1.72	0.85	-	1.01	0.54 to 1.91	0.97	-
17: shoulder injury (never)	ref	ref	ref	-	ref	ref	ref	-
17: shoulder injury (<6 months)	2.14	0.38 to 11.89	0.39	-	1.83	0.31 to 11.00	0.51	-
17: shoulder injury (6-12 months)	1.07	0.15 to 7.71	0.95	-	0.80	0.10 to 6.32	0.83	-
17: shoulder injury (>12months)	2.49	0.63 to 9.87	0.19	-	1.58	0.37 to 6.62	0.53	-
18: lumbar spine injury (never)	ref	ref	ref	-	ref	ref	ref	-
18: lumbar spine injury (<6 months)	0.93	0.23 to 3.80	0.92	-	1.70	0.40 to 7.18	0.47	-
18: lumbar spine injury (6-12 months)	0.69	0.15 to 3.18	0.64	-	0.86	0.18 to 4.18	0.85	-
18: lumbar spine injury (>12months)	0.55	0.26 to 1.14	0.11	-	0.80	0.37 to 1.73	0.57	-
19: iliopsoas IMI (never)	ref	ref	ref	-	ref	ref	ref	-
19: iliopsoas IMI (<6 months)	0.98	0.06 to 15.77	0.99	-	1.01	0.06 to 17.67	0.99	-
19: iliopsoas IMI (6-12 months)	0.39	0.07 to 2.05	0.27	-	0.54	0.10 to 2.92	0.48	-
19: iliopsoas IMI (>12months)	0.73	0.25 to 2.17	0.57	-	0.81	0.26 to 2.50	0.71	-
20: hip adductor IMI (never)	ref	ref	ref	-	ref	ref	ref	-
20: hip adductor IMI (<6 months)	1.24	0.46 to 3.32	0.68	-	1.00	0.35 to 2.83	0.99	-
20: hip adductor IMI (6-12 months)	1.65	0.45 to 6.00	0.45	-	1.24	0.32 to 4.84	0.75	-
20: hip adductor IMI (>12months)	1.88	0.71 to 4.96	0.20	-	1.36	0.47 to 3.90	0.57	-
21: hamstring IMI (never)	ref	ref	ref	-	ref	ref	ref	-
21: hamstring IMI (<6 months)	4.06	0.80 to 20.64	0.09	-	2.64	0.49 to 14.29	0.26	-
21: hamstring IMI (6-12 months)	1.76	0.74 to 4.21	0.20	-	1.42	0.58 to 3.52	0.44	-
21: hamstring IMI (>12months)	**4.74**	**2.05 to 10.93**	**<0.001**	**Linear**	**3.03**	**1.25 to 7.36**	**0.01**	**Linear**
22: quadriceps IMI (never)	**ref**	ref	**ref**	-	**ref**	**ref**	**ref**	**-**
22: quadriceps IMI (<6 months)	4.11	0.45 to 37.37	0.21	-	4.09	0.42 to 39.53	0.22	-
22: quadriceps IMI (6-12 months)	0.51	0.15 to 1.76	0.29	-	0.51	0.14 to 1.85	0.31	-
22: quadriceps IMI (>12months)	1.21	0.52 to 2.83	0.65	-	1.13	0.47 to 2.77	0.77	-
23: calf IMI (never)	ref	ref	ref	-	ref	ref	ref	-
23: calf IMI (<6 months)	4.44	0.92 to 21.36	0.06	-	3.62	0.71 to 18.47	0.12	-
23: calf IMI (>6 months)*	1.66	0.66 to 4.23	0.28	-	0.79	0.27 to 2.33	0.67	-
**Musculoskeletal:**								
24. Mean PROM hip IR (deg.)	**0.97**	**0.94 to 0.99**	**0.01**	**Linear**	0.98	0.95 to 1.01	0.12	-
25. Mean PROM hip ER (deg.)	**0.96**	**0.93 to 1.00**	**0.03**	**Linear**	0.99	0.95 to 1.02	0.46	-
26. Mean hip flexor length (deg.)	1.02	0.99 to 1.05	0.15	-	1.03	0.99 to 1.07	0.07	-
27. Mean hamstring/neural mobility length (deg.)	0.99	0.95 to 1.02	0.44	-	0.97	0.93 to 1.01	0.15	-
28. Mean calf muscle length (deg.)	0.99	0.96 to 1.02	0.45	-	0.99	0.96 to 1.02	0.34	-

**Strength/Power:**								
29: Max. leg extension power difference (W/kg$^{-0.67}$)	0.98	0.91 to 1.06	0.58	-	0.97	0.90 to 1.06	0.48	-
30: Mean of max. leg extension power (W/kg$^{-0.67}$)	1.03	0.99 to 1.06	0.14	-	1.00	0.97 to 1.04	0.77	-
31: Max. leg extension velocity difference (m.s^{-1})	2.28	0.28 to 18.29	0.44	-	3.24	0.37 to 28.60	0.29	-
32: Mean of max. leg extension velocity (m.s^{-1})	2.44	0.77 to 7.72	0.13	-	1.65	0.46 to 5.80	0.44	-
33: Max leg extension force difference (N/kg$^{-0.67}$)	0.99	0.97 to 1.01	0.50	-	0.99	0.98 to 1.01	0.44	-
34: Mean of max. leg extension force (N/kg$^{-0.67}$)	1.00	0.99 to 1.01	0.94	-	0.99	0.98 to 1.02	0.63	-
35: CMJ Force per kg of body mass (N/kg)	0.99	0.92 to 1.06	0.80	-	0.98	0.90 to 1.06	0.60	-
36: CMJ height (cm)	1.03	0.98 to 1.09	0.25	-	1.00	0.95 to 1.07	0.85	-

Key: PHE= periodic health examination; PF= prognostic factor; OR=odds ratio; CI=confidence interval; ref=reference category; I-IMI=index indirect muscle injury; IMI= indirect muscle injury; Freq= frequency; WBL=weight bearing lunge; CMJ=countermovement jump; PROM=passive range of movement; deg. = degrees; SLR= straight leg raise; BMI= body mass index; kg/m^2 = kilograms/body height (metres) squared; *cm = centimetres; Kg=kilograms; W= watts (note: W/kg$^{-0.67}$ has a scaling factor to normalise force to body mass[42]); N= newtons (note: N/kg$^{-0.67}$ has a scaling factor to normalise force to body mass);max.=maximum; m.s.= metres per second; cm = centimetres; Kg=kilograms; - = not applicable; * indicates merged category (using calf IMI (6-12 months) and calf IMI (>12months). This was required due to the occurrence of perfect predictions in the Calf (6-12 months) category as a consequence of reduced sample size.* ***Note:*** *ORs are expressed per one-unit increase for all continuous factors, and according to category for all categorical factors; Factors in* ***bold*** *indicate significance at the 0.05 level.*
