# Supplementary material for: Can prognostic factors for indirect muscle injuries in elite football (soccer) players be identified using data from preseason screening? An exploratory analysis using routinely collected periodic health examination records
Source: BMJ Open. 2023 Jan 24;13(1):e052772. doi: 10.1136/bmjopen-2021-052772 (PMC9884927; doi:10.1136/bmjopen-2021-052772)
Supplement: Supplementary data [file bmjopen-2021-052772supp004.pdf]

Does preseason screening provide a source of potential prognostic factors for indirect muscle injuries in elite football (soccer) players? An exploratory analysis using routinely-collected periodic health examination data

Hughes, T., Riley, R.D., Callaghan, M.J. and Sergeant, J.C. (2022)

Supplementary file 4: Anthropometric parameters and all included candidate prognostic factor characteristics for participants included in the sensitivity analyses

Characteristic/candidate prognostic factors		***Min***	***Lower quartile***	***Median***	***Upper quartile***	***Max.***	***Freq. (%) - if categorical***	***Missing values n (%)***
**Anthropometrics**								
1. Age at PHE (years)		16.01	17.64	19.20	24.30	39.59	-	0 (0)
2. Height (cm)		164.30	176.10	180.00	185.50	195.00	-	15 (5.77)
3. Weight (kg)		56.80	68.09	73.10	80.00	94.00	-	16 (6.15)
**Past medical history**								
Within 3 years prior to PHE, freq. of:								
4. foot/ankle injuries		0.00	0.00	1.00	2.00	7.00	-	0 (0)
5. Hip/groin injuries		0.00	0.00	0.00	1.00	5.00	-	
6. Knee injuries		0.00	0.00	0.00	1.00	3.00	-	0 (0)
7. Shoulder injuries		0.00	0.00	0.00	0.00	2.00	-	0 (0)
8. lumbar spine injuries		0.00	0.00	0.00	0.00	3.00	-	0 (0)
9. iliopsoas IMIs		0.00	0.00	0.00	0.00	2.00	-	0 (0)
10. hip adductor IMIs		0.00	0.00	0.00	0.00	3.00	-	0 (0)
11.hamstring IMIs		0.00	0.00	0.00	1.00	5.00	-	0 (0)
12. quadriceps IMIs		0.00	0.00	0.00	0.00	3.00	-	0 (0)
13. calf IMIs		0.00	0.00	0.00	0.00	4.00	-	0 (0)
**Within 3 years prior to PHE, most recent:**								
14. foot/ankle injury	*Never*	-	-	-	-	-	120 (46.15)	0 (0)
	< 6 months	-	-	-	-	-	37 (14.23)	0 (0)
	6-12 months	-	-	-	-	-	29 (11.15)	0 (0)
	>12 months	-	-	-	-	-	74 (28.46)	0 (0)
15. Hip/groin injury	*Never*	-	-	-	-	-	176 (67.69)	0 (0)
	< 6 months	-	-	-	-	-	21 (8.08)	0 (0)
	6-12 months	-	-	-	-	-	20 (7.69)	0 (0)
	>12 months	-	-	-	-	-	43 (16.54)	0 (0)
16. Knee injury	*Never*	-	-	-	-	-	165 (63.46)	0 (0)
	< 6 months	-	-	-	-	-	15 (5.77)	0 (0)
	6-12 months	-	-	-	-	-	24 (9.23)	0 (0)
	>12 months	-	-	-	-	-	56 (21.54)	0 (0)
17. Shoulder injury	*Never*	-	-	-	-	-	240 (92.31)	0 (0)
	< 6 months	-	-	-	-	-	6 (2.31)	0 (0)
	6-12 months	-	-	-	-	-	4 (1.54)	0 (0)
	>12 months	-	-	-	-	-	10 (3.85)	0 (0)
18. lumbar spine injury	*Never*	-	-	-	-	-	210 (80.77)	0 (0)
	< 6 months	-	-	-	-	-	8 (3.08)	0 (0)
	6-12 months	-	-	-	-	-	7 (2.69)	0 (0)
	>12 months	-	-	-	-	-	35 (13.46)	0 (0)
19. iliopsoas IMI	*Never*	-	-	-	-	-	237 (91.15)	0 (0)
	< 6 months	-	-	-	-	-	2 (0.77)	0 (0)
	6-12 months	-	-	-	-	-	7 (2.69)	0 (0)
	>12 months	-	-	-	-	-	14 (5.38)	0 (0)
20. hip adductor IMI	*Never*	-	-	-	-	-	214 (82.31)	0 (0)
	< 6 months	-	-	-	-	-	17 (6.54)	0 (0)
	6-12 months	-	-	-	-	-	10 (3.85)	0 (0)
	>12 months	-	-	-	-	-	19 (7.31)	0 (0)
21.hamstring muscle IMI	*Never*	-	-	-	-	-	193 (74.23)	0 (0)
	< 6 months	-	-	-	-	-	8 (3.08)	0 (0)
	6-12 months	-	-	-	-	-	23 (8.85)	0 (0)
	>12 months	-	-	-	-	-	36 (13.85)	0 (0)
22. quadriceps IMI	*Never*	-	-	-	-	-	165 (63.46)	0 (0)
	< 6 months	-	-	-	-	-	5 (1.92)	0 (0)
	6-12 months	-	-	-	-	-	12 (4.62)	0 (0)
	>12 months	-	-	-	-	-	24 (9.23)	0 (0)
23. calf IMI	*Never*	-	-	-	-	-	230 (88.46)	0 (0)
	< 6 months	-	-	-	-	-	10 (3.85)	0 (0)
	6-12 months	-	-	-	-	-	5 (1.92)	0 (0)
	>12 months	-	-	-	-	-	15 (5.77)	0 (0)
**Musculoskeletal Examination**								
24. Mean PROM hip IR (deg.)		9.50	22.00	32.50	40.00	55.00	-	15 (5.77)
25. Mean PROM hip ER (deg.)		17.50	33.70	39.00	43.50	62.00	-	15 (5.77)
26. Mean hip flexor length (deg.)		-7.00	3.00	8.68	15.00	55.00	-	18 (6.92)
27. Mean hamstring/neural mobility length (deg.)		45.00	83.00	90.00	90.00	102.00	-	15 (5.77)
28. Mean calf muscle length (deg.)		9.50	25.65	30.50	37.00	57.50	-	15 (5.77)
**Strength/Power Tests**								
29: Max. leg extension power difference ($W/kg^{-0.67}$)		-11.94	-1.47	0.43	1.91	15.26	-	39 (15.00)
30: Mean of max. leg extension power ($W/kg^{-0.67}$)		23.01	41.67	46.40	51.10	78.70	-	39 (15.00)
31: Max. leg extension velocity difference ($m.s^{-1}$)		-0.34	-0.05	0.02	0.11	0.40	-	35 (13.46)
32: Mean of max. leg extension velocity ($m.s^{-1}$)		1.02	1.68	1.84	1.98	2.35	-	35 (13.46)
33: Max leg extension force difference ($N/kg^{-0.67}$)		-83.59	-5.74	-1.07	4.39	55.41	-	39 (15.00)
34: Mean of max. leg extension force ($N/kg^{-0.67}$)		50.19	90.85	101.14	112.02	217.96	-	39 (15.00)

35: CMJ Force per kg of body mass	20.50	23.30	25.18	27.90	39.20	-	35 (13.46)
36: CMJ height (cm)	28.70	37.20	39.90	43.10	58.00	-	35 (13.46)

Key: PHE= periodic health examination; I-IMI=index indirect muscle injury; IMI= indirect muscle injury; min = minimum; max = maximum; n = observations; Freq= frequency; WBL=weight bearing lunge; CMJ=countermovement jump; PROM=passive range of movement; deg. = degrees; SLR= straight leg raise; BMI= body mass index; kg/m^2 = kilograms/body height (metres) squared; *cm = centimetres; Kg=kilograms; Cont.=continuous; dis./cont.= discrete treated as continuous; cat.= categorical. Note that for the Musculoskeletal Examination and Strength/Power factors, positive values indicate greater left limb values compared to right limb values; negative values indicate greater right limb*
