# Supplementary material for: Can prognostic factors for indirect muscle injuries in elite football (soccer) players be identified using data from preseason screening? An exploratory analysis using routinely collected periodic health examination records
Source: BMJ Open. 2023 Jan 24;13(1):e052772. doi: 10.1136/bmjopen-2021-052772 (PMC9884927; doi:10.1136/bmjopen-2021-052772)
Supplement: Supplementary data [file bmjopen-2021-052772supp010.pdf]

Does preseason screening provide a source of potential prognostic factors for indirect muscle injuries in elite football (soccer) players? An exploratory analysis using routinely-collected periodic health examination data

Hughes, T., Riley, R.D., Callaghan, M.J. and Sergeant, J.C. (2022)

Supplementary File 10: Comparison of candidate prognostic factors with statistically significant associations across the primary and sensitivity multivariable analyses (using imputed and complete case datasets).

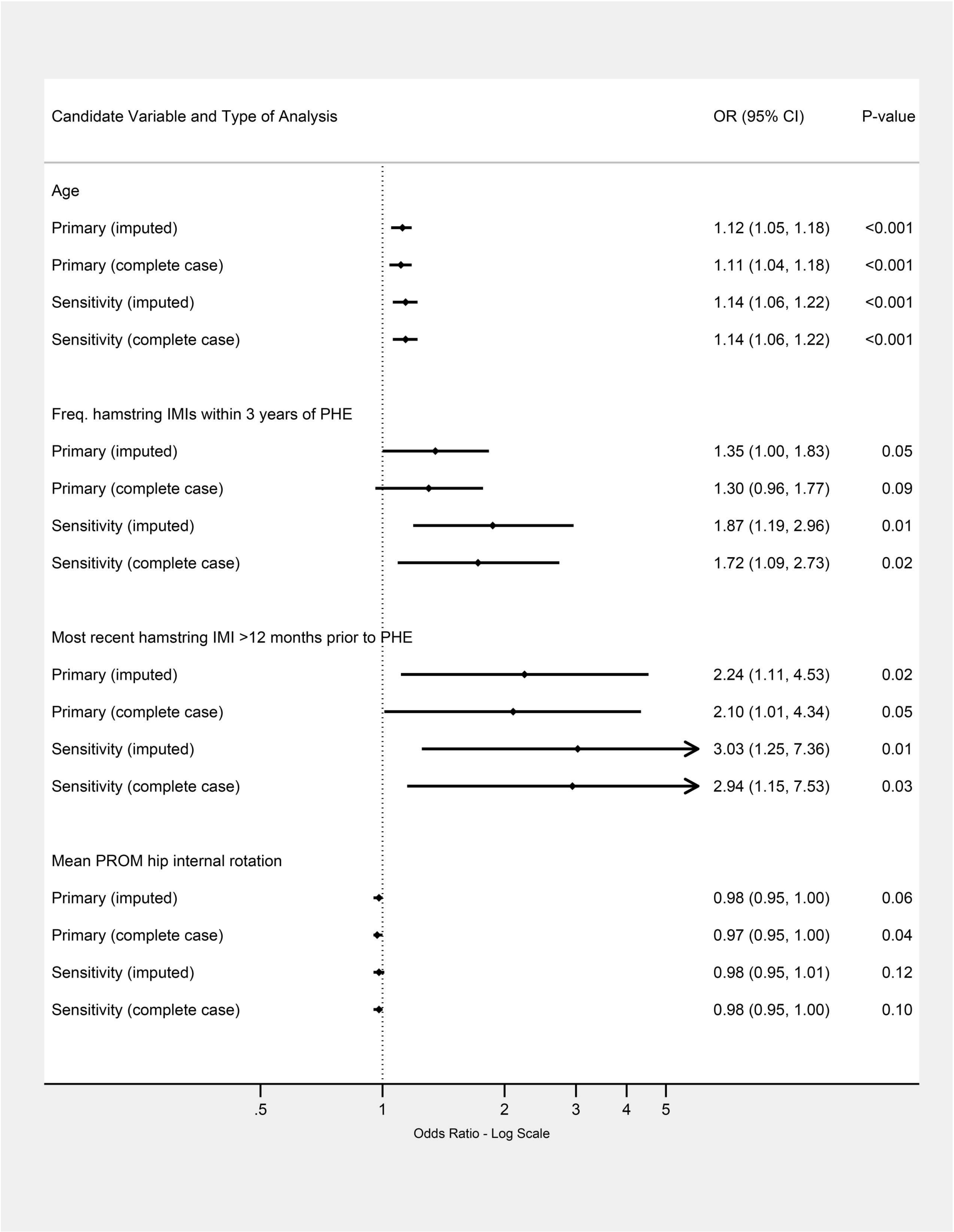

Key: IMI = indirect muscle injury; OR = odds ratio; freq. = frequency; PHE = periodic health examination; PROM = passive range of movement.

Note: the factor of age was adjusted for height and weight. All other factors were adjusted for age, height and weight.
