# Supplementary material for: Can prognostic factors for indirect muscle injuries in elite football (soccer) players be identified using data from preseason screening? An exploratory analysis using routinely collected periodic health examination records
Source: BMJ Open. 2023 Jan 24;13(1):e052772. doi: 10.1136/bmjopen-2021-052772 (PMC9884927; doi:10.1136/bmjopen-2021-052772)
Supplement: Author's manuscript [file bmjopen-2021-052772.draft_revisions.pdf]

BMJ Open

BMJ Open is committed to open peer review. As part of this commitment we make the peer review history of every article we publish publicly available.

When an article is published we post the peer reviewers' comments and the authors' responses online. We also post the versions of the paper that were used during peer review. These are the versions that the peer review comments apply to.

The versions of the paper that follow are the versions that were submitted during the peer review process. They are not the versions of record or the final published versions. They should not be cited or distributed as the published version of this manuscript.

BMJ Open is an open access journal and the full, final, typeset and author-corrected version of record of the manuscript is available on our site with no access controls, subscription charges or pay-per-view fees (http://bmjopen.bmj.com).

If you have any questions on BMJ Open's open peer review process please email info.bmjopen@bmj.com

BMJ Open

Can prognostic factors for indirect muscle injuries in elite football (soccer) players be identified using data from preseason screening? An exploratory analysis using routinely-collected periodic health examination records.

Journal:	*BMJ Open*
Manuscript ID	bmjopen-2021-052772
Article Type:	Original research
Date Submitted by the Author:	26-Apr-2021

Complete List of Authors:	Hughes, Tom; Manchester United Football Club; University of Manchester<br>Riley, Richard; Keele University<br>Callaghan, Michael J.; Manchester United Football Club; Manchester Metropolitan University<br>Sergeant, Jamie; University of Manchester, Arthritis Research UK Centre for Epidemiology; Manchester Academic Health Science Centre, Centre for Biostatistics

Keywords:	SPORTS MEDICINE, REHABILITATION MEDICINE, Musculoskeletal disorders < ORTHOPAEDIC & TRAUMA SURGERY

SCHOLARONE™
Manuscripts

I, the Submitting Author has the right to grant and does grant on behalf of all authors of the Work (as defined in the below author licence), an exclusive licence and/or a non-exclusive licence for contributions from authors who are: i) UK Crown employees; ii) where BMJ has agreed a CC-BY licence shall apply, and/or iii) in accordance with the terms applicable for US Federal Government officers or employees acting as part of their official duties; on a worldwide, perpetual, irrevocable, royalty-free basis to BMJ Publishing Group Ltd ("BMJ") its licensees and where the relevant Journal is co-owned by BMJ to the co-owners of the Journal, to publish the Work in this journal and any other BMJ products and to exploit all rights, as set out in our licence.

The Submitting Author accepts and understands that any supply made under these terms is made by BMJ to the Submitting Author unless you are acting as an employee on behalf of your employer or a postgraduate student of an affiliated institution which is paying any applicable article publishing charge ("APC") for Open Access articles. Where the Submitting Author wishes to make the Work available on an Open Access basis (and intends to pay the relevant APC), the terms of reuse of such Open Access shall be governed by a Creative Commons licence – details of these licences and which Creative Commons licence will apply to this Work are set out in our licence referred to above.

Other than as permitted in any relevant BMJ Author's Self Archiving Policies, I confirm this Work has not been accepted for publication elsewhere, is not being considered for publication elsewhere and does not duplicate material already published. I confirm all authors consent to publication of this Work and authorise the granting of this licence.

For peer review only

Can prognostic factors for indirect muscle injuries in elite football (soccer) players be identified using data from preseason screening? An exploratory analysis using routinely-collected periodic health examination records.

Tom Hughes, PhD,[1 2]
Professor Richard D. Riley, PhD,[3]
Professor Michael J. Callaghan, PhD, *[1,2,5]
Dr. Jamie C. Sergeant, PhD, * [2 4]

[1] Manchester United Football Club, AON Training Complex, Birch Road, Off Isherwood Road, Carrington, Manchester. UK.

[2] Centre for Epidemiology Versus Arthritis, Centre for Musculoskeletal Research, Manchester Academic Health Science Centre, University of Manchester, Manchester, UK.

[3] Centre for Prognosis Research, School of Medicine, Keele University, Staffordshire, UK.

[4] Centre for Biostatistics, University of Manchester, Manchester Academic Health Science Centre, Manchester, UK.

[5] Department of Health Professions, Manchester Metropolitan University, Brooks Building, Bonsall Street, Manchester. UK.

***JCS and MJC contributed equally and are joint last authors**

Corresponding author: Tom Hughes

Email: tom.hughes.physio@manutd.co.uk
Twitter: @dt_hughes
ORCID ID: orcid.org/0000-0003-2266-6615

Correspondence address: Manchester United Football Club, AON Training Complex, Birch Road, Off Isherwood Road, Carrington, Manchester. UK. M31 4BH.
Tel: 0161 868 8754

WORD COUNT: 3989 (excluding title, abstract, tables, figures and references)

For peer review only

ABSTRACT

Background: In elite football, periodic health examination (PHE) may be useful for injury risk prediction.

Objective: To explore whether PHE-derived variables are prognostic factors for indirect muscle injuries (IMIs) in elite players.

Design: Retrospective cohort study

Setting: An English Premier League football club.

Participants: 134 outfield elite male players, over 5 seasons (1st July 2013 to 19th May 2018).

Outcome and analysis: The outcome was any time-loss, lower extremity index indirect muscle injury (I-IMI). Prognostic associations were estimated using odds ratios (ORs) and corresponding statistical significance for 36 variables, derived from univariable and multivariable logistic regression models. Missing data were handled using multiple imputation. Non-linear associations were explored using fractional polynomials.

Results: During 317 participant-seasons, 138 I-IMIs were recorded. Univariable associations were determined for previous calf IMI frequency (OR=1.80, 95% confidence interval (CI) = 1.09 to 2.97), hamstring IMI frequency (OR=1.56, 95% CI=1.17 to 2.09), if the most recent hamstring IMI occurred > 12 months but < 3 years prior to PHE (OR= 2.95, 95% CI = 1.51 to 5.73) and age (OR =1.12 per 1-year increase, 95% CI = 1.06 to 1.18). Multivariable analyses showed that if a player's most recent previous hamstring IMI was >12 months but <3 years prior to PHE (OR= 2.24, 95% CI = 1.11 to 4.53), this was the only variable with added prognostic value over and above age, which was a confirmed prognostic factor (OR=1.12 per 1-year increase, 95%CI = 1.05 to 1.18). Allowing non-linear associations conferred no advantage over linear ones.

Conclusion: PHE has limited use for injury risk prediction. Most variables did not add prognostic value over and above age, other than if a player experienced a hamstring IMI >12 months but < 3 years prior to PHE. However, the precision of this prognostic association should be confirmed in future.

Trial registration number = NCT03782389

WORD COUNT = 300

For peer review only - http://bmjopen.bmj.com/site/about/guidelines.xhtml

STRENGTHS AND LIMITATIONS OF THIS STUDY

- This study investigated a wide selection of PHE-derived variables and their association with index indirect muscle injuries in a cohort of elite football players.
- We examined the prognostic value of these PHE-derived variables over and above standard anthropometric variables of age (which has a previously confirmed prognostic association), height and weight.
- This is the first known study in elite football to explore any non-linear associations for variables that produce continuous data, using a fractional polynomial approach.
- High-quality cohort data were used, with variables reliably measured in pre-season and any subsequent injury outcomes confirmed using a validated muscle injury classification system.
- For practical reasons, our imputation model did not assume non-linear associations and therefore may have reduced our ability to detect genuine non-linear relationships in the subsequent analyses; this is unlikely to be a material concern however, because the quantity of missing data for all factors was always less than 15%.
- We also only measured candidate factors at one timepoint each season, which means that dynamic associations were not investigated and we assumed that participant-seasons were independent.

For peer review only

For peer review only - http://bmjopen.bmj.com/site/about/guidelines.xhtml

BACKGROUND

Periodic health examination (PHE), or screening, is a well-established clinical evaluation strategy in elite football.[1] Typically during PHE, players undertake various medical, musculoskeletal, functional and performance tests[2] during preseason and in-season periods.[1] PHE allows opportunities for general health surveillance, identification of salient pathology[3] and monitoring of rehabilitation or performance.[4] PHE is also assumed to have an important role in the development of injury prevention strategies.[5 6] In particular, although PHE cannot establish specific causal factors for injuries,[4] it is perceived to be useful for the prediction of future injury risk in athletes,[2 4] where accurate predictions could help to identify individuals who may require interventions that are designed to reduce risk.[7]

To make such predictions, prognostic factors are required which, in the context of football, could be any variables, characteristics or measurements available at or derived from PHE (e.g. medical history, leg strength or range of motion tests) that are associated with increased injury risk.[4] The predictive power of a single prognostic factor is usually limited on its own.[8 9] However, if several prognostic factors are used in combination within a multivariable prognostic model, it may be possible to produce useful individualised risk estimates.[8 10]

Because the predictive function of PHE remains unsubstantiated[3 11] and given that indirect (non-contact) muscle injuries (IMIs) are a significant problem observed in elite football (accounting for 30.3% to 47.9% of all injuries),[12-16] we have recently developed and internally validated a multivariable prognostic model to predict individualised lower extremity IMI risk in elite players using PHE data.[17] However, sample size limitations meant that only 10 candidate prognostic factors could be considered in our model and these were selected using data quality assessment, clinical reasoning, or on the basis of our related

For peer review only - http://bmjopen.bmj.com/site/about/guidelines.xhtml

systematic review.[11] The performance of our model was modest and we concluded that its use would not be beneficial in clinical practice.[17]

Furthermore, our previous systematic review also highlighted several methodological limitations of the current evidence, which specifically included inadequate reporting of outcomes, prognostic factor measurement and reliability.[11] Additionally, while most studies performed appropriate statistical analyses, continuous prognostic factor measurements were often categorised[18-21] and non-linear associations were not investigated,[18-24] which does not conform to current methodological recommendations.[25-27]

To assist the development of future prognostic models, there is a clear need to ascertain the existence of robust and novel prognostic factors that have an association with IMIs.[17] Therefore, using routinely collected data from a 5-season period, we conducted an exploratory cohort study to examine: 1) prognostic associations between PHE-derived variables and IMI outcomes in elite footballers, using a wider set of candidates than had previously been considered[17] and; 2) the prognostic value of these PHE-derived variables over and above standard anthropometric variables of age (which has previously confirmed prognostic value[11 17]), height and weight. In particular, both linear and non-linear associations between candidate variables and outcomes were explored which, to the best of our knowledge, has not been conducted previously.

METHODS

The methodology has been described in a published protocol[28] so will only be briefly outlined. This study was registered on ClinicalTrials.gov (NCT03782389) and was reported according to the Reporting Recommendations for Marker Prognostic Studies (REMARK).[29]

For peer review only - http://bmjopen.bmj.com/site/about/guidelines.xhtml

Given the number of PHE-related variables examined, our study should be viewed as exploratory, but we emphasise that this an important phase in prognostic factor research.[9 30]

Data Sources

This study was of retrospective cohort design. Eligible participants were identified from a population of male elite footballers, aged 16-40 years old at Manchester United Football Club. A database was created by the principal investigator (TH) using routinely collected injury records and preseason PHE data over 5 seasons (1st July 2013 to 19th May 2018). This process included checks for accuracy, duplicate or missing entries. Participants completed a mandatory PHE during the first week of each season (which started on 1st July), and were followed up to the last first team game of the season.

The PHE process typically included: 1) anthropometric measurements; 2) a review of medical and previous injury history; 3) musculoskeletal examination tests; 4) functional movement and balance tests; 5) strength and power tests. The PHE test order was self-selected by each player and a standardised warm up was not implemented, although players could undertake their own warm up procedures if they wished. Each component of PHE was standardised according to a written protocol and was examined by physiotherapists, sports scientists or club medical doctors. The same examiners performed the same test every season to eliminate inter-tester variability. No examiner attrition occurred throughout the data collection period. If a participant was injured at the scheduled time of PHE, a risk assessment was completed by medical staff and participants only completed tests that were deemed appropriate and safe for the participant's condition; examiners were therefore not blinded to injury status.

For peer review only - http://bmjopen.bmj.com/site/about/guidelines.xhtml

Eligibility criteria

During any season, participants were eligible if they: 1) were not a goalkeeper; 2) participated in PHE for the relevant season. Participants were excluded if they were not under contract to the Club at the time of PHE.

Ethics and Data Use

Informed consent was not required as data were captured from the mandatory PHE completed through the participants' employment. The data usage was approved by the Club and the Research Ethics Service at the University of Manchester.

Participant involvement

Participants were not involved in the study design.

Outcome

The outcome was any time-loss, index lower extremity IMI (I-IMI) sustained by a participant during match play or training, which affected any lower abdominal, hip, thigh, calf or foot muscle groups and prohibited future match or training participation.[31] I-IMIs were confirmed and graded by a club doctor or physiotherapist according to the previously validated Munich Consensus Statement for the Classification of Muscle Injuries in Sport,[32 33] during routine assessments undertaken within 24h of injury occurrence. The medical professionals were not blinded to PHE data at diagnosis.

For peer review only - http://bmjopen.bmj.com/site/about/guidelines.xhtml

Sample size

Our sample size of 317 participant-seasons (with 138 I-IMI events) had 80% power to detect an adjusted odds ratio (OR) of at least 1.6 for a 1 standard deviation increase in a variable of interest, conservatively assuming a correlation of 0.5 with the adjustment variables of age, height and weight (see Supplementary File 1 for the sample size calculation).[34]

PHE-derived Candidate Variables

The dataset contained 60 variables[28] that were eligible for analysis unless there were >15% missing observations or if reliability (where applicable) was reported as fair to poor (that is, Intraclass Correlation Coefficient (ICC) < 0.70).[28 35] If any variables did not meet these eligibility criteria, they were excluded (Supplementary File 2). Collinearity between eligible variables was assessed with a scatterplot matrix; this was evident when tests were used to measure right and left limbs independently.[28] In these circumstances, composite variables were created for between-limb differences and the mean of the test measurements for both limbs, as described in the study protocol.[28]

Of the remaining eligible variables, 10 were used in our multivariable prognostic model study (represented by 12 parameters).[17] With the exception of age at PHE (which was used for adjustment purposes in this study), these candidates were therefore excluded.[28] The final number of candidate variables included for exploratory analysis was 36. Table 1 summarises all included variables with their measurement units and data type, as well as the methods and reliability of measurement.

For peer review only - http://bmjopen.bmj.com/site/about/guidelines.xhtml

Table 1: List of candidate variables with a summary of the units of measurement, methods and reliability of measurement and data type.

Outcome & Variable type		*Name of Outcome/Variable*	*Measurement unit*	*Measurement method*	*Reliability (if applicable/available)*	*Data type*
**Outcome**		Index indirect muscle injury (I-IMI)	Binary yes/no	Medical records; cross referenced against Munich Classification Criteria	-	Binary
**Anthropometric variables**		1: age;<br>2: height;<br>3: weight;	1: years;<br>2: centimetres (cm);<br>3: kilograms (kg)	1: Medical records;<br>2: Standing height measure;<br>3: Digital scales	-	Cont.
**Medical history variables**	*Within 3 years prior to PHE, the frequency of:*	4: foot/ankle injuries; 5: hip/groin injuries;<br>6: knee injuries; 7: shoulder injuries;<br>8: lumbar spine injuries;<br>9: iliopsoas IMIs;<br>10: hip adductor IMIs;<br>11: hamstring IMIs;<br>12: quadriceps IMIs;<br>13: calf IMIs	4-13: Count	4-13: Medical records	-	Dis./cont.
	Within 3 years prior to PHE, the most recent:	14: foot/ankle injury; 15: hip/groin injury;<br>16: knee injury; 17: shoulder injury;<br>18: lumbar spine injury;<br>19: iliopsoas IMI;<br>20: hip adductor IMI;<br>21: hamstring IMI;<br>22: quadriceps IMI;<br>23: calf IMI	14-23: Never, < 6 months, 6-12 months, > 12 months	14-23: Medical records	-	Cat.
**Musculoskeletal variables**		24: Mean PROM hip internal rotation (IR);<br>25: Mean PROM hip external rotation (ER);<br>26: Mean hip flexor length;<br>27: Mean hamstring length/neural mobility;<br>28: Mean calf muscle length	24-28: Degrees	24-25: Digital inclinometer + ROM;<br>26: Digital inclinometer + Thomas Test;<br>27: Digital inclinometer + SLR;<br>28: Digital inclinometer + WBL	24-25: Intra-rater ICC= 0.90;[36]<br>26: Inter-rater ICC = 0.89;[37]<br>27: Intra-rater ICC = 0.95-0.98;[38] Inter-rater ICC = 0.80-0.97;[38]<br>28: Inter-rater ICC = 0.80- 0.95;[39 40] Intra-rater ICC = 0.88.[40]	Cont.
**Strength/power variables**		29: Max. leg extension power difference;<br>30: Mean of max. leg extension power;<br>31: Max. leg extension velocity difference;<br>32: Mean of max. leg extension velocity;<br>33: Max leg extension force difference;<br>34: Mean of max. leg extension force;<br>35: CMJ Force per kg of body mass;<br>36: CMJ height	29-30: Normalised watts per kilo ($W/kg^{-0.67}$);<br>31-32: Peak velocity ($m.s^{-1}$);<br>33-34: Normalised peak force ($N/Kg^{-0.67}$);<br>35: Force per	29-34: Double leg press using Keiser Air 300 machine;<br>35-36: Countermovement jump (CMJ) + force plate	29-30: Test-retest ICC = 0.886;[41]<br>31-32: Test-retest ICC = 0.792;[41]<br>33-34: Test-retest ICC = 0.914;[41]<br>35-36: Test-retest ICC = 0.80-0.88.[42]	Cont.

For peer review only

	kg (N/kg);
	36: Centimetres (cm)

Key: BMI= body mass index; cat.= categorical; Cont.=continuous; cm = centimetres; CMJ=countermovement jump; deg. = degrees; dis./cont.= discrete treated as continuous; grp=group; ICC=intraclass correlation coefficient; I-IMI= index indirect muscle injury; Kg=kilograms; kg/m² = kilograms/body height squared; max.=maximum; m.s.= metres per second; N= newtons (note: N/kg$^{-0.67}$ has a scaling factor to normalise force to body mass [43]); PROM=passive range of movement; SLR= straight leg raise; W= watts (note: W/kg$^{-0.67}$ has a scaling factor to normalise force to body mass [43]); WBL=weight bearing lunge.

Statistical analysis

Data handling – outcome measures

Each participant-season was treated as independent. If an I-IMI occurred, the participant's outcome was determined for that season and they were no longer considered at risk. In these circumstances, participants were included for further analysis at the start of the consecutive season, if still eligible. Any upper limb IMI, trunk IMI or non-IMI injuries were ignored and participants were still considered at risk.

Eligible participants who were loaned to another club throughout that season, but had not sustained an I-IMI prior to the loan were still considered at risk. I-IMIs that occurred whilst on loan were included for analysis. Permanently transferred participants (who had not sustained an I-IMI prior to the transfer), were recorded as not having an I-IMI during the relevant season and exited the cohort at the season end.

Data Handling – Missing data

Missing values were assumed to be missing at random (i.e. missingness could be predicted conditional on other known variables).[28] The continuous parameters generally demonstrated non-normal distributions, so were transformed using normal scores[44] to approximate normality before imputation, and back-transformed following imputation.[45] Multivariate

For peer review only - http://bmjopen.bmj.com/site/about/guidelines.xhtml

normal multiple imputation was performed, using a model that included all candidate variables and I-IMI outcomes. Fifty imputed datasets were created in Stata 15.1 (StataCorp LLC, Texas, USA) using the '*mi impute*' command.

Univariable and multivariable analyses

All data were analysed in the form that they were recorded. In particular, variables that were recorded as continuous were kept continuous and not categorised, to avoid a loss of prognostic information.[25] Univariable logistic regression models were used to estimate the unadjusted linear associations between I-IMIs and each candidate variable. Multivariable logistic regression models were also used to estimate the linear association between I-IMIs and each variable, after adjustment for age (which has confirmed prognostic importance[11 17]), height and weight (which were both considered as potential confounders for I-IMIs and PHE-derived candidates). All parameter estimates were averaged across all imputed datasets using Rubin's Rules[46] and were computed using the '*mim*' module in Stata 15.1. Statistical significance thresholds were used to indicate the strength of exploratory evidence against null associations, where p-values of : 1) <0.05 indicated strong evidence and the factor was considered significant; 2) 0.05 to 0.10 indicated weak evidence and; 3) >0.10 indicated little or no evidence.[47] Prognostic importance was also considered by checking the magnitude of prognostic effects encompassed by the width of 95% confidence intervals.

For all variables, non-linear associations with the outcome were also explored using fractional polynomials for the univariable and multivariable models; the fit of first and second order fractional polynomial models were evaluated against the fit of the standard logistic regression models.[48] The parameter estimates were combined across all imputed

For peer review only - http://bmjopen.bmj.com/site/about/guidelines.xhtml

datasets[49] using Rubins Rules,[46] with the automated '*mfpmi*' algorithm in Stata 15.1, using a significance threshold set at $p < 0.05$. All analyses are summarised in Table 2.

Table 2: Summary of all statistical analyses performed

Statistical analyses of I-IMI outcomes					
**Analysis performed**	**Participant-seasons**	**Number of I-IMIs**	**Variables considered**	**Adjusted for:**	**Results**
Primary analysis, imputed data					
A1: Univariable Logistic Regression/FPs	317	138	Individual models for I-IMI + each variable (1-36)	None	Table 4
A2: Multivariable Logistic Regression/FPs	317	138	Individual models for I-IMI + each variable (4-36)	Variables 1-3 (age, height, weight)	Table 4
Primary analysis, complete case data					
B1: Univariable Logistic Regression/FPs	265	115	Individual models for I-IMI + each variable (1-36)	None	SF 5
B2: Multivariable Logistic Regression/FPs	265	115	Individual models for I-IMI + each variable (4-36)	Variables 1-3 (age, height, weight)	SF 5
Sensitivity analysis, imputed data					
C1: Univariable Logistic Regression/FPs	260	129	Individual models for I-IMI + each variable (1-36)	None	SF 6
C2: Multivariable Logistic Regression/FPs	260	129	Individual models for I-IMI + each variable (4-36)	Variables 1-3 (age, height, weight)	SF 6
Sensitivity analysis, complete case data					
D1: Univariable Logistic Regression/FPs	217	106	Individual models for I-IMI + each variable (1-36)	None	SF 7
D2: Multivariable Logistic Regression/FPs	217	106	Individual models for I-IMI + each variable (4-36)	Variables 1-3 (age, height, weight)	SF 7

Key: FP= fractional polynomials; I-IMI= index indirect muscle injury; SF= supplementary file

Primary and sensitivity analyses

To determine the effect of imputation and player transfers on variable associations, the analyses were repeated: 1) as complete cases analyses; and 2) as sensitivity analyses excluding participant-seasons for participants who were loaned or transferred (performed as both multiple imputation and complete case analyses). All primary complete case and sensitivity analyses are also summarised in Table 2.

RESULTS

Participants

During the five seasons, 134 participants were included, contributing 317 participant-seasons and 138 IMIs in the primary analysis (Figure 1). Three players were classified as injured at

For peer review only - http://bmjopen.bmj.com/site/about/guidelines.xhtml

For peer review only

the time of PHE (which affected three participant-seasons). This meant they were unavailable for selection for training or matches at that time. However, these players had commenced football specific, field-based rehabilitation so also had similar exposure to training activities to uninjured players and were therefore included in the cohort because it was reasonable to assume that they could also be considered at risk of an I-IMI event.

For the sensitivity analyses (excluding loans and transfers), 260 independent participant-seasons with 129 IMIs were included; 36 participants were transferred on loan, while 14 participants were permanently transferred during a season, which excluded 57 participant-seasons (Figure 1).

INSERT FIGURE 1 HERE

Table 3 summarises the participant characteristics and candidate variable values for participants included in the primary analyses. All values were similar to those included in the sensitivity analyses (Supplementary File 3).

Missing data and multiple imputation

Data were complete for age and all past medical history variables (Table 3). For all other candidates, the proportion of missing data ranged from 5.68% (for height and weight) to 14.20% (for the mean and between limb differences of maximal leg extension power and force) (Table 3). For all continuous variables, the distribution of imputed values approximated the observed values (Supplementary File 4), therefore confirming their plausibility.

For peer review only - http://bmjopen.bmj.com/site/about/guidelines.xhtml

Table 3. Characteristics of included participants

Characteristics/candidate variables		*Min*	*Lower quartile*	*Median*	*Upper quartile*	*Max.*	*Freq. (%) - if categorical*	*Missing values n (%)*
**Anthropometrics**								
1. Age at PHE (years)		16.01	17.80	19.69	23.56	39.59	-	0 (0)
2. Height (cm)		164.3	176.0	180.0	185.5	195.0	-	18 (5.68)
3. Weight (kg)		56.8	69.2	73.6	80.0	94.0	-	18 (5.68)
**Past medical history**								
Within 3 years prior to PHE, freq. of:								
4. foot/ankle injuries		0	0	1	2	7	-	0 (0)
5. Hip/groin injuries		0	0	0	1	5	-	0 (0)
6. Knee injuries		0	0	0	1	3	-	0 (0)
7. Shoulder injuries		0	0	0	0	2	-	0 (0)
8. lumbar spine injuries		0	0	0	0	3	-	0 (0)
9. iliopsoas IMIs		0	0	0	0	2	-	0 (0)
10. hip adductor IMIs		0	0	0	0	3	-	0 (0)
11.hamstring IMIs		0	0	0	1	6	-	0 (0)
12. quadriceps IMIs		0	0	0	0	3	-	0 (0)
13. calf IMIs		0	0	0	0	4	-	0 (0)
Within 3 years prior to PHE, most recent:								
14. foot/ankle injury	*Never*	-	-	-	-	-	143 (45.11)	0 (0)
	< 6 months	-	-	-	-	-	43 (13.56)	0 (0)
	6-12 months	-	-	-	-	-	34 (10.73)	0 (0)
	>12 months	-	-	-	-	-	97 (30.60)	0 (0)
15. Hip/groin injury	*Never*	-	-	-	-	-	217 (68.45)	0 (0)
	< 6 months	-	-	-	-	-	23 (7.26)	0 (0)
	6-12 months	-	-	-	-	-	23 (7.26)	0 (0)
	>12 months	-	-	-	-	-	54 (17.03)	0 (0)
16. Knee injury	*Never*	-	-	-	-	-	201 (63.41)	0 (0)
	< 6 months	-	-	-	-	-	15 (4.73)	0 (0)
	6-12 months	-	-	-	-	-	31 (9.78)	0 (0)
	>12 months	-	-	-	-	-	70 (22.08)	0 (0)
17. Shoulder injury	*Never*	-	-	-	-	-	297 (93.69)	0 (0)
	< 6 months	-	-	-	-	-	6 (1.89)	0 (0)
	6-12 months	-	-	-	-	-	4 (1.26)	0 (0)
	>12 months	-	-	-	-	-	10 (3.15)	0 (0)
18. lumbar spine injury	*Never*	-	-	-	-	-	264 (83.28)	0 (0)
	< 6 months	-	-	-	-	-	8 (2.52)	0 (0)
	6-12 months	-	-	-	-	-	9 (2.84)	0 (0)
	>12 months	-	-	-	-	-	36 (11.36)	0 (0)
19. iliopsoas IMI	*Never*	-	-	-	-	-	287 (90.54)	0 (0)
	< 6 months	-	-	-	-	-	2 (0.63)	0 (0)
	6-12 months	-	-	-	-	-	9 (2.84)	0 (0)
	>12 months	-	-	-	-	-	19 (5.99)	0 (0)
20. hip adductor IMI	*Never*	-	-	-	-	-	263 (82.92)	0 (0)
	< 6 months	-	-	-	-	-	18 (5.68)	0 (0)
	6-12 months	-	-	-	-	-	12 (3.79)	0 (0)
	>12 months	-	-	-	-	-	24 (7.57)	0 (0)
21.hamstring IMI	*Never*	-	-	-	-	-	231 (72.87)	0 (0)
	< 6 months	-	-	-	-	-	11 (3.47)	0 (0)
	6-12 months	-	-	-	-	-	30(9.46)	0 (0)
	>12 months	-	-	-	-	-	45 (14.20)	0 (0)
22. quadriceps IMI	*Never*	-	-	-	-	-	267 (84.23)	0 (0)
	< 6 months	-	-	-	-	-	7 (2.21)	0 (0)
	6-12 months	-	-	-	-	-	13 (4.10)	0 (0)
	>12 months	-	-	-	-	-	30 (9.46)	0 (0)
23. calf IMI	*Never*	-	-	-	-	-	283 (89.27)	0 (0)
	< 6 months	-	-	-	-	-	11 (3.47)	0 (0)
	6-12 months	-	-	-	-	-	6 (1.89)	0 (0)
	>12 months	-	-	-	-	-	17 (5.36)	0 (0)
**Musculoskeletal Examination**								
24. Mean PROM hip IR (deg.)		9.5	22.5	33.0	40.0	55.0	-	20 (6.31)
25. Mean PROM hip ER (deg.)		17.5	33.5	38.5	43.0	62.0	-	20 (6.31)
26. Mean hip flexor length (deg.)		-7.0	3.5	9.0	15.0	55.0	-	23 (7.26)
27. Mean hamstring/neural mobility length (deg.)		45.0	84.0	90.0	90.0	102.0	-	20 (6.31)
28. Mean calf muscle length (deg.)		9.5	25.0	30.0	36.0	57.5	-	20 (6.31)
**Strength/Power Tests**								
29: Max. leg extension power difference ($W/kg^{-0.67}$)		-11.94	-1.55	0.29	1.77	15.26	-	45 (14.20)
30: Mean of max. leg extension power ($W/kg^{-0.67}$)		23.01	42.12	46.52	51.95	78.69	-	45 (14.20)

For peer review only

31: Max. leg extension velocity difference ($m.s^{-1}$)	-0.34	-0.05	0.02	0.11	0.40	-	41 (12.93)
32: Mean of max. leg extension velocity ($m.s^{-1}$)	1.02	1.68	1.84	1.98	2.35	-	41 (12.93)
33: Max leg extension force difference ($N/kg^{-0.67}$)	-83.59	-6.18	1.17	4.40	55.41	-	45 (14.20)
34: Mean of max. leg extension force ($N/kg^{-0.67}$)	50.19	98.59	101.44	113.44	217.95	-	45 (14.20)
35: CMJ Force per kg of body mass	20.20	23.40	25.40	28.04	39.20	-	42 (13.25)
36: CMJ height (cm)	28.7	37.3	40.2	43.0	58.0	-	42 (13.25)

Key: BMI= body mass index; cat.= categorical; Cont.=continuous; cm = centimetres; CMJ=countermovement jump; deg. = degrees; dis./cont.= discrete treated as continuous; Freq= frequency; grp=group; ICC=intraclass correlation coefficient; IMI= indirect muscle injury; I-IMI= index indirect muscle injury; IQR= interquartile range; Kg=kilograms; kg/m^2 = kilograms/body height squared; max.=maximum; m.s.= metres per second; N= newtons (note: $N/kg^{-0.67}$ has a scaling factor to normalise force to body mass [43]); PHE= periodic health examination; PROM=passive range of movement; SLR= straight leg raise; W= watts (note: $W/kg^{-0.67}$ has a scaling factor to normalise force to body mass [43]); WBL=weight bearing lunge; - = not applicable. Note that for the Musculoskeletal Examination and Strength/Power factors, positive values indicate greater left limb values compared to right limb values; negative values indicate greater right limb values compared to left limb values.

For peer review only

Univariable analyses

Table 4 shows the results of the univariable analyses. The continuous variables of age (OR= 1.12 for a 1-year increase, 95% confidence interval (CI)=1.06 to 1.18, p<0.001), weight (OR=1.03 for a 1kg increase, 95% CI=1.00 to 1.07, p=0.03) and mean hip IR PROM (OR= 0.97 for a 1-degree increase, 95% CI = 0.95 to 0.99, p=0.01) showed a significant but modest association with I-IMIs. The narrow CIs indicated that these estimates were relatively precise. Linear associations were the best fit for all these continuous variables. Significant associations with larger OR estimates were observed for previous calf IMI frequency (OR=1.80, 95% CI = 1.09 to 2.97, p=0.02), hamstring IMI frequency (OR=1.56, 95% CI=1.17 to 2.09, p<0.001), and if the most recent hamstring IMI occurred more than 12 months but less than 3 years prior to PHE (OR= 2.95, 95% CI = 1.51 to 5.73, p<0.001). The wider CIs for these estimates indicated greater imprecision about the prognostic effect; this may because these candidates were either discrete or categorical, rather than continuous.

Despite relatively large ORs, weaker evidence of associations was observed for the frequency of previous shoulder injuries (OR=2.38, 95% CI = 0.98 to 5.75, p=0.05) and if the most recent calf IMI was less than 6 months prior to PHE (OR=3.78, 95% CI=0.98 to 14.56,

For peer review only - http://bmjopen.bmj.com/site/about/guidelines.xhtml

p=0.05). However, the very wide CIs indicated considerable uncertainty about the true OR. No other significant candidate factors were observed.

Multivariable analyses

Table 4 shows the results of the multivariable analyses, where the adjusted prognostic value was evaluated for all PHE-derived variables. After adjustment for height and weight, age remained significantly associated with increased odds of sustaining an I-IMI during a season (OR=1.12 for a 1-year increase, 95%CI = 1.05 to 1.18, p<0.001) and a linear association was the best fit for this variable. However, there was no evidence that height and weight were strong prognostic factors independent of age.

After adjustment for age, height and weight, if the most recent hamstring IMI was more than 12 months but less than 3 years prior to PHE, the significant association and wide CI also remained (OR= 2.24, 95% CI = 1.11 to 4.53, p=0.02). However, no other candidates demonstrated prognostic importance. For most variables, the magnitude of the adjusted prognostic association was also smaller than the unadjusted association and some confidence intervals were very wide.

For peer review only - http://bmjopen.bmj.com/site/about/guidelines.xhtml

Table 4: Univariable and multivariable logistic regression estimates for all candidate prognostic factors

	Univariable (unadjusted)				Multivariable (adjusted for age, height, weight)			
**Candidate PF & Type**	OR	95% CI	P	Best model fit	OR	95% CI	P	Best model fit
**Anthropometric (Adjustment Factors):**								
1: Age (years)	**1.12**	**1.06-1.18**	**<0.001**	**Linear**	**1.12**	**1.05 to 1.18**	**<0.001**	**Linear**
2: Height (cm)	1.03	0.99 to 1.07	0.13	-	1.02	0.97 to 1.07	0.46	-
3: Weight (kg)	**1.03**	**1.00 to 1.07**	**0.03**	**Linear**	1.00	0.95 to 1.04	0.91	-
**Within 3 years preceding PHE, frequency of:**								
4: foot/ankle injuries	1.04	0.87 to 1.24	0.68	-	1.04	**0.87 to 1.25**	0.65	-
5: hip/groin injuries	1.16	0.90 to 1.51	0.25	-	1.29	0.99 to 1.70	0.06	-
6: knee injuries	0.96	0.72 to 1.29	0.81	-	0.98	0.72 to 1.32	0.88	-
7: shoulder injuries	2.38	0.98 to 5.75	0.05	-	1.89	0.76 to 4.74	0.17	-
8: lumbar spine injuries	0.88	0.60 to 1.29	0.50	-	1.08	0.72 to 1.61	0.71	-
9: iliopsoas IMIs	0.73	0.38 to 1.43	0.37	-	0.86	0.43 to 1.71	0.67	-
10: hip adductor IMIs	1.38	0.92 to 2.09	0.12	-	1.18	0.76 to 1.84	0.46	-
11: hamstring IMIs	**1.56**	**1.17 to 2.09**	**<0.001**	**Linear**	1.35	1.00 to 1.83	0.05	-
12: quadriceps IMIs	1.08	0.67 to 1.73	0.75	-	1.05	0.65 to 1.71	0.84	-
13: calf IMIs	**1.80**	**1.09 to 2.97**	**0.02**	**Linear**	1.30	0.75 to 2.25	0.35	-
**Within 3 years preceding PHE, most recent:**								
14: foot/ankle injury (never)	ref	ref	ref	-	ref	ref	ref	-
14: foot/ankle injury (<6 months)	1.27	0.64 to 2.53	0.49	-	1.41	0.69 to 2.87	0.35	-
14: foot/ankle injury (6-12 months)	1.16	0.54 to 2.46	0.71	-	1.15	0.52 to 2.52	0.73	-
14: foot/ankle injury (>12 months)	1.27	0.75 to 2.13	0.37	-	1.22	0.71 to 2.09	0.47	-
15: hip/groin injury (never)	ref	ref	ref	-	ref	ref	ref	-
15: hip/groin injury (<6 months)	1.05	0.44 to 2.49	0.92	-	1.66	0.67 to 4.13	0.27	-
15: hip/groin injury (6-12 months)	0.59	0.23 to 1.50	0.27	-	0.65	0.25 to 1.69	0.38	-
15: hip/groin injury (>12months)	1.58	0.87 to 2.87	0.14	-	1.80	0.96 to 3.36	0.07	-
16: knee injury (never)	ref	ref	ref	-	ref	ref	ref	-
16: knee injury (<6 months)	1.15	0.40 to 3.28	0.80	-	1.12	0.37 to 3.34	0.84	-
16: knee injury (6-12 months)	1.23	0.58 to 2.62	0.60	-	1.24	0.57 to 2.70	0.60	-
16: knee injury (>12months)	0.93	0.53 to 1.61	0.79	-	0.99	0.56 to 1.75	0.96	-
17: shoulder injury (never)	ref	ref	ref	-	ref	ref	ref	-
17: shoulder injury (<6 months)	2.75	0.50 to 15.26	0.25	-	2.55	0.44 to 14.72	0.30	-
17: shoulder injury (6-12 months)	1.38	0.19 to 9.90	0.75	-	1.11	1.15 to 8.36	0.92	-
17: shoulder injury (>12months)	3.21	0.81 to 12.67	0.10	-	2.36	0.58 to 9.62	0.23	-
18: lumbar spine injury (never)	ref	ref	ref	-	ref	ref	ref	-
18: lumbar spine injury (<6 months)	1.24	0.30 to 5.05	0.77	-	2.10	0.50 to 8.82	0.31	-
18: lumbar spine injury (6-12 months)	0.62	0.15 to 2.53	0.50	-	0.75	0.18 to 3.14	0.69	-
18: lumbar spine injury (>12months)	0.70	0.34 to 1.44	0.33	-	0.97	0.46 to 2.06	0.94	-
19: iliopsoas IMI (never)	ref	ref	ref	-	ref	ref	ref	-
19: iliopsoas IMI (<6 months)	1.24	0.77 to 20.05	0.88	-	1.27	0.08 to 21.32	0.87	-
19: iliopsoas IMI (6-12 months)	0.62	0.15 to 2.53	0.51	-	0.83	0.20 to 3.43	0.80	-
19: iliopsoas IMI (>12months)	0.57	0.21 to 1.55	0.27	-	0.66	0.24 to 1.82	0.42	-
20: hip adductor IMI (never)	ref	ref	ref	-	ref	ref	ref	-
20: hip adductor IMI (<6 months)	1.37	0.53 to 3.56	0.52	-	1.29	0.48 to 3.47	0.61	
20: hip adductor IMI (6-12 months)	1.37	0.43 to 4.36	0.60	-	1.16	0.35 to 3.81	0.81	-
20: hip adductor IMI (>12months)	1.37	0.59 to 3.16	0.46	-	1.14	0.47 to 2.79	0.77	-
21: hamstring IMI (never)	ref	ref	ref	-	ref	ref	ref	-
21: hamstring IMI (<6 months)	2.84	0.81 to 9.99	0.10	-	2.07	0.57 to 7.56	0.27	-
21: hamstring IMI (6-12 months)	1.42	0.66 to 3.06	0.37	-	1.22	0.56 to 2.70	0.62	-
21: hamstring IMI (>12months)	**2.95**	**1.51 to 5.73**	**<0.001**	**Linear**	**2.24**	**1.11 to 4.53**	**0.02**	**Linear**
22: quadriceps IMI (never)	ref	ref	ref	-	ref	ref	ref	-
22: quadriceps IMI (<6 months)	1.74	0.38 to 7.91	0.48	-	1.56	0.33 to 7.36	0.58	-
22: quadriceps IMI (6-12 months)	0.58	0.17 to 1.93	0.37	-	0.59	0.17 to 2.04	0.41	-
22: quadriceps IMI (>12months)	1.14	0.53 o 2.43	0.74	-	1.07	0.49 to 2.35	0.86	-
23: calf IMI (never)	ref	ref	ref	-	ref	ref	ref	-
23: calf IMI (<6 months)	3.78	0.98 to 14.56	0.05	-	3.11	0.78 to 12.46	0.11	-
23: calf IMI (6-12 months)	7.09	0.82 to 61.51	0.08	-	3.80	0.41 to 35.41	0.24	-
23: calf IMI (>12months)	1.26	0.47 to 3.36	0.64	-	0.73	0.24 to 2.15	0.56	-
**Musculoskeletal:**								
24. Mean PROM hip IR (deg.)	**0.97**	**0.95 to 0.99**	**0.01**	**Linear**	0.98	0.95 to 1.00	0.06	-
25. Mean PROM hip ER (deg.)	0.97	0.95 to 1.00	0.09	-	0.99	0.96 to 1.02	0.53	-
26. Mean hip flexor length (deg.)	1.01	0.98 to 1.04	0.46	-	1.01	0.99 to 1.04	0.32	-

For peer review only

27. Mean hamstring/neural mobility length (deg.)	0.99	0.96 to 1.02	0.53	-	0.98	0.95 to 1.02	0.33	-
28. Mean calf muscle length (deg.)	1.00	0.97 to 1.02	0.77	-	1.00	0.97 to 1.02	0.79	-
**Strength/Power:**								
29: Max. leg extension power difference (W/kg$^{-0.67}$)	0.99	0.92 to 1.07	0.84	-	0.99	0.91 to 1.06	0.71	-
30: Mean of max. leg extension power (W/kg$^{-0.67}$)	1.02	0.99 to 1.05	0.24	-	1.01	0.97 to 1.04	0.73	-
31: Max. leg extension velocity difference (m.s^{-1})	2.15	0.31 to 14.88	0.44	-	2.83	0.38 to 21.31	0.31	-
32: Mean of max. leg extension velocity (m.s^{-1})	1.96	0.68 to 5.64	0.21	-	1.48	0.49 to 4.47	0.49	-
33: Max leg extension force difference (N/kg$^{-0.67}$)	0.99	0.98 to 1.02	0.76	-	1.00	0.98 to 1.02	0.65	-
34: Mean of max. leg extension force (N/kg$^{-0.67}$)	1.00	0.99 to 1.01	1.00	-	1.00	0.99 to 1.01	0.72	-
35: CMJ Force per kg of body mass (N/kg)	0.99	0.93 to 1.06	0.78	-	0.99	0.92 to 1.07	0.80	-
36: CMJ height (cm)	1.03	0.98 to 1.08	0.27	-	0.72	0.96 to 1.07	0.63	-

Key: BMI= body mass index; cm = centimetres; CI=confidence interval; CMJ=countermovement jump; deg. = degrees; Freq= frequency; IMI= indirect muscle injury; I-IMI=index indirect muscle injury; Kg=kilograms; kg/m^2 = kilograms/body height (metres) squared; *max.=maximum; m.s.= metres per second; N= newtons (note: N/kg$^{-0.67}$ has a scaling factor to normalise force to body mass[43]); OR=odds ratio; PF= prognostic factor; PHE= periodic health examination; PROM=passive range of movement; ref=reference category; SLR= straight leg raise; W= watts (note: W/kg$^{-0.67}$ has a scaling factor to normalise force to body mass[43]); WBL=weight bearing lunge; - = not applicable.*

Note: *ORs are expressed per one-unit increase for all continuous factors, and according to category for all categorical factors; Factors in* ***bold*** *indicate significance at the 0.05 level.*

For peer review only

Complete case and sensitivity analysis

The results of all complete case and sensitivity analyses are presented in Supplementary Files 5-7. Supplementary File 8 shows a forest plot of the estimates obtained for all statistically significant candidate prognostic factors across all primary and sensitivity univariable analyses. Supplementary File 9 shows a forest plot of the estimates obtained for all statistically significant candidate prognostic factors across all multivariable analyses.

For both univariable and multivariable analyses, the prognostic associations were very similar for the complete case and imputation analyses. Sensitivity analyses (that is, excluding players who were loaned or transferred) led to slightly stronger prognostic associations for some factors (for example, the frequency and timing of previous hamstring injuries), although the CIs were wider.

For peer review only - http://bmjopen.bmj.com/site/about/guidelines.xhtml

DISCUSSION

This exploratory study has examined linear and non-linear prognostic associations between PHE-derived variables and I-IMIs in elite football players, using routinely collected data from a 5-season period.

While the univariable analyses suggested 6 candidate factors are associated with I-IMIs, such analyses are limited as they only provide baseline association measures prior to adjustment for other important prognostic factors.[29] However, after adjustment for age, height and weight in the multivariable analyses, we found that most PHE derived variables did not add any prognostic value over and above age. The exception was that if a hamstring IMI was sustained by a player more than 12 months (but less than 3 years) prior to PHE, their odds of sustaining a lower extremity I-IMI significantly increased 2.2-fold, which has not been previously reported. Although not directly comparable, earlier studies have also shown that a history of a previous hamstring IMI is specifically associated with an increased hazard of future hamstring IMIs in elite players.[11 20 22] Nevertheless, the uncertainty in our estimates (demonstrated by wide 95% confidence intervals) and differences observed during the sensitivity analyses mean that this variable only has provisional prognostic value and needs to be established in further confirmatory studies.

Indeed, age was the only variable that could be considered as an important prognostic factor, which is easily obtained even without conducting PHE; after allowing for height and weight, age retained a highly significant, modest and precise association that was robust to both imputation and player transfers. For illustration, our estimates suggest that for every 1-year increase in age, the odds of sustaining an I-IMI during a season would increase by approximately 12%. As an example (to put into the context of absolute risk), for two players

For peer review only - http://bmjopen.bmj.com/site/about/guidelines.xhtml

who were the same height and weight but aged 5 years apart, if the younger-aged player had a risk of 0.44 (which was overall outcome prevalence in our study), then the older player would have a risk of 0.58.

Our findings confirm those of our multivariable prognostic model study, where age was retained in a model to determine individualised I-IMI risk over a season in elite players (OR = 1.10, 95% CI = 1.03 to 1.17).[17] Other studies have shown that as a multivariable prognostic factor, age is associated specifically with increased hamstring IMI risk (OR range 1.40-1.78),[11 18 23] where the reported estimates were larger than those observed in our study. These differences may be due to chance or partly because we merged all lower extremity I-IMI outcomes rather than utilising IMI subgroups, which may have diluted the strength of our observed associations. However, although our approach was less clinically meaningful, merging I-IMI outcomes was essential in order to maximise the statistical power of our study.

Importantly, while multiple exploratory studies have confirmed the prognostic importance of age,[11 17 18 23] it is not a causal factor for future IMI occurrence. Rather, it is likely to be a proxy marker for another potential causal mechanism, although to establish the true causal role of a prognostic factor, associations should be demonstrated during additional confirmatory and explanatory studies.[50] Even taking this into account, injury prevention strategies have been traditionally developed to modify supposed causal factors,[6] but because age is non-modifiable it could not be used to inform this process in a clinical setting. Instead, age should currently only be considered clinically useful to explain differences in risk between players in a team, or included as an important factor in future prognostic model development studies.

For peer review only - http://bmjopen.bmj.com/site/about/guidelines.xhtml

Using data from PHE tests that measure modifiable physical and performance characteristics has been previously questioned for injury prediction purposes.[2] Our results fully support this view, because none of the modifiable musculoskeletal (clinical examination) or strength and power tests evaluated showed any statistically significant associations with I-IMIs. This absence of strong associations mean that such tests have poor discriminatory ability, usually because of overlap in test scores that occur in individuals who sustain a future injury and those who do not.[2] Furthermore, after measurement at a solitary timepoint (i.e. pre-season), it is likely that the prognostic value of modifiable factors is time-varying[51] as a consequence of physical and physiological adaptations that occur from training exposure and other injuries.[52]

Overall, our findings suggest that at present, PHE cannot be considered a useful source of prognostic factors for IMI risk prediction and injury prevention practice in elite football players. Instead, PHE should only be deemed clinically useful for screening of pathology that could pose a risk to safe participation and for rehabilitation or performance monitoring purposes. However, because of our study's exploratory nature (with many estimates having very wide confidence intervals), the shortcomings of the current evidence base and the paucity of known prognostic factors in elite football,[11] there is a clear need for further investigation in this area to improve our understanding of the prognostic value of PHE in elite football and other sports.

Limitations and future research

Our study is unique in that we have investigated non-linear associations as per methodological guidelines.[9 53] However, in our analyses linear associations were selected over non-linear associations. For practical reasons, our imputation model did not assume non-linear associations and therefore may have reduced our ability to detect genuine non-linear

For peer review only - http://bmjopen.bmj.com/site/about/guidelines.xhtml

relationships in the subsequent analyses. However, this is not a concern for age, as there were no missing values for this factor and is unlikely to be a material concern for all other factors as missing data was always less than 15%.

A competing risks analysis was not conducted, which meant that individuals who sustained injury types other than lower extremity IMIs were still considered at risk, even though this may have affected their training and match exposure and hence risk of sustaining an I-IMI event. We also only measured candidate factors at one timepoint each season, which means that dynamic associations were not investigated. We also assumed that participant-seasons were independent. Future studies could account for competing risks, utilise repeated measurements over time and incorporate between-season correlations into analyses. However, the complexity such analyses would also require a significantly larger volume of data. This could be achieved through data sharing initiatives and individual participant data meta-analysis, which would also increase the power to detect genuine prognostic associations and non-linear relationships.

CONCLUSION

We have evaluated prognostic associations between PHE-derived candidate variables and lower extremity I-IMIs in elite football players using data that were routinely collected over 5 seasons. We found no clear associations for nearly all PHE variables, although if a player sustained a hamstring IMI greater than 12 months (but less than 3 years) prior to PHE, then this had potential prognostic value over and above the prognostic value of age. Indeed, age was the only variable to be confirmed as a clear prognostic factor in both univariable and multivariable analyses. However, this is easily measured without then need to conduct PHE, and although it has limited application in clinical practice, it should be included as an

For peer review only - http://bmjopen.bmj.com/site/about/guidelines.xhtml

important factor in any future prognostic model development studies. Our findings suggest that overall, PHE cannot be currently considered as a useful source of prognostic factors for I-IMI risk prediction and injury prevention practice in elite football players. Instead, it should only be utilised for screening of pathology, as well as rehabilitation and performance monitoring. Further research is required to confirm the prognostic value of PHE for IMI risk prediction and to identify novel prognostic factors that could improve development of prognostic models in football and other sports.

ACKNOWLEDGEMENTS

The authors would like to thank all staff within the Medical and Sports Science Department at Manchester United for their continuing help and support with this manuscript and thank all players for their participation (without whom this study would not be possible). The authors also thank Versus Arthritis for their support: Versus Arthritis grant number 21755.

CONTRIBUTORS

TH was responsible for the conceptualisation of the project, study design, database construction, data extraction and cleaning, protocol development and protocol writing. TH conducted the data analysis, interpretation and wrote the main manuscript. RR provided statistical guidance and assisted with development of the study design, analysis, and edited manuscript drafts. MC assisted with the study conceptualisation and design, protocol development, clinical interpretation and editing the manuscript drafts. JS provided guidance with the study design, development of the analysis and protocol, interpretation of the analysis, as well as editing the study manuscripts. All authors read and approved this final manuscript.

For peer review only

CONFLICT OF INTEREST

None declared.

FUNDING

The lead researcher (TH) is receiving sponsorship from Manchester United Football Club to complete a postgraduate PhD study programme. This work was also supported by Versus Arthritis: grant number 21755.

ETHICS AND DATA USE

Informed consent was not required as data were captured from the mandatory PHE completed through the participants' employment. The data usage was approved by the football club and the Research Ethics Service at the University of Manchester.

DATA SHARING STATEMENT

An anonymised summary of the dataset that was analysed during this study may be available from the corresponding author on reasonable request.

PATIENT CONSENT FOR PUBLICATION

Not required.

For peer review only

References:

1. McCall A, Carling C, Davison M, et al. Injury risk factors, screening tests and preventative strategies: a systematic review of the evidence that underpins the perceptions and practices of 44 football (soccer) teams from various premier leagues. *Br J Sports Med* 2015;49(9):583-89.

2. Bahr R. Why screening tests to predict injury do not work-and probably never will...: a critical review. *Br J Sports Med* 2016;50(13):776-80. doi: 10.1136/bjsports-2016-096256.

3. Ljungqvist A, Jenoure PJ, Engebretsen AH, et al. The International Olympic Committee (IOC) Consensus Statement on Periodic Health Evaluation of Elite Athletes, March 2009. *Clin J Sport Med* 2009;19(5):347-60.

4. Hughes T, Sergeant JC, van der Windt DA, et al. Periodic Health Examination and Injury Prediction in Professional Football (Soccer): Theoretically, the Prognosis is Good. *Sports Med* 2018;48(11):2443-48. doi: 10.1007/s40279-018-0928-y.

5. Drew MK, Cook J, Finch CF. Sports-related workload and injury risk: simply knowing the risks will not prevent injuries. *Br J Sports Med* 2016;50:1306-08. doi: 10.1136/bjsports-2015-095871.

6. Jacobsson J, Timpka T. Classification of Prevention in Sports Medicine and Epidemiology. *Sports Med* 2015;45(11):1483-7. doi: 10.1007/s40279-015-0368-x.

7. Localio AR. Beyond the Usual Prediction Accuracy Metrics: Reporting Results for Clinical Decision Making. *Ann Intern Med* 2012;157(4):294-96.

8. Riley RD, Hayden JA, Steyerberg EW, et al. Prognosis Research Strategy (PROGRESS) 2: prognostic factor research. *PLoS Med* 2013;10(2):e1001380. doi: https://doi.org/10.1371/journal.pmed.1001380.

9. Riley RD, van der Windt DA, Croft P, et al. Prognosis Research in Healthcare: Concepts, Methods and Impact. Oxford: Oxford University Press 2019.

10. Steyerberg EW, Moons KG, van der Windt DA, et al. Prognosis Research Strategy (PROGRESS) 3: prognostic model research. *PLoS Med* 2013;10(2):e1001381. doi: https://doi.org/10.1371/journal.pmed.1001381.

11. Hughes T, Sergeant JC, Parkes M, et al. Prognostic factors for specific lower extremity and spinal musculoskeletal injuries identified through medical screening and training load monitoring in professional football (soccer): a systematic review. *BMJ Open Sporst Ex Med* 2017;3(1):1-18. doi: 10.1136/bmjsem-2017- 000263.

12. Ekstrand J, Hagglund M, Walden M. Epidemiology of muscle injuries in professional football (soccer). *Am J Sports Med* 2011;39(6):1226-32. doi: 10.1177/0363546510395879.

For peer review only - http://bmjopen.bmj.com/site/about/guidelines.xhtml

13. Falese L, Della Valle P, Federico B. Epidemiology of football (soccer) injuries in the 2012/2013 and 2013/2014 seasons of the Italian Serie A. *Res Sports Med* 2016;24(4):426-32. doi: 10.1080/15438627.2016.1239105.

14. Larruskain J, Lekue JA, Diaz N, et al. A comparison of injuries in elite male and female football players: A five-season prospective study. *Scand J Med Sci Sports* 2018;28(1):237-45. doi: 10.1111/sms.12860.

15. Leventer L, Eek F, Hofstetter S, et al. Injury Patterns among Elite Football Players: A Media-based Analysis over 6 Seasons with Emphasis on Playing Position. *Int J Sports Med* 2016;37(11):898-908. doi: 10.1055/s-0042-108201.

16. Hawkins RD, Fuller CW. A prospective epidemiological study of injuries in four English professional football clubs. *Br J Sports Med* 1999;33(3):196-203.

17. Hughes T, Riley RD, Callaghan MJ, et al. The Value of Preseason Screening for Injury Prediction: The Development and Internal Validation of a Multivariable Prognostic Model to Predict Indirect Muscle Injury Risk in Elite Football (Soccer) Players. *Sports Medicine - Open* 2020;6(22) doi: https://doi.org/10.1186/s40798-020-00249-8.

18. Arnason A, Sigurdsson SB, Gudmundsson A, et al. Risk factors for injuries in football. *Am J Sports Med* 2004;32(1 Suppl):5S-16S.

19. Fousekis K, Tsepis E, Poulmedis P, et al. Intrinsic risk factors of non-contact quadriceps and hamstring strains in soccer: a prospective study of 100 professional players. *Br J Sports Med* 2011;45(9):709-14.

20. Hägglund M, Waldén M, Ekstrand J. Risk Factors for Lower Extremity Muscle Injury in Professional Soccer: The UEFA Injury Study. *Am J Sports Med* 2013;41(2):327-35.

21. Timmins RG, Bourne MN, Shield AJ, et al. Short biceps femoris fascicles and eccentric knee flexor weakness increase the risk of hamstring injury in elite football (soccer): a prospective cohort study. *Br J Sports Med* 2016;50(24):1524-35. doi: 10.1136/bjsports-2015-095362.

22. Hägglund M, Waldén M, Ekstrand J. Previous injury as a risk factor for injury in elite football: a prospective study over two consecutive seasons. *Br J Sports Med* 2006;40(9):767-72.

23. Henderson G, Barnes CA, Portas MD. Factors associated with increased propensity for hamstring injury in English Premier League soccer players. *J Sci Med Sport* 2010;13(4):397-402. doi: 10.1016/j.jsams.2009.08.003.

24. van Dyk N, Bahr R, Whiteley R, et al. Hamstring and Quadriceps Isokinetic Strength Deficits Are Weak Risk Factors for Hamstring Strain Injuries: A 4-Year Cohort Study. *Am J Sports Med* 2016;44(7):1789-95. doi: 10.1177/0363546516632526.

For peer review only - http://bmjopen.bmj.com/site/about/guidelines.xhtml

25. Moons KG, Altman DG, Reitsma JB, et al. Transparent Reporting of a multivariable prediction model for Individual Prognosis or Diagnosis (TRIPOD): explanation and elaboration. *Ann Intern Med* 2015;162(1):W1-73. doi: 10.7326/M14-0698.
26. Royston P, Altman DG, Sauerbrei W. Dichotomizing continuous predictors in multiple regression: a bad idea. *Stat Med* 2006;25(1):127-41. doi: 10.1002/sim.2331.

27. Altman DG, Royston P. The cost of dichotomising continuous variables. *Br Med J* 2006;332:1080.

28. Hughes T, Riley R, Sergeant J, et al. A study protocol for the development and internal validation of a multivariable prognostic model to determine lower extremity muscle injury risk in elite football (soccer) players, with further exploration of prognostic factors. *Diagn Progn Res* 2019;3(19) doi: 10.1186/s41512-019-0063-8.

29. Sauerbrei W, Taube SE, McShane LM, et al. Reporting Recommendations for Tumor Marker Prognostic Studies (REMARK): An Abridged Explanation and Elaboration. *J Natl Cancer Inst* 2018;110(8):803-11. doi: 10.1093/jnci/djy088.

30. Altman DG, Lyman GH. Methodological challenges in the evaluation of prognostic factors in breast cancer. *Breast Cancer Res Treat* 1998;52:289-303.

31. Fuller CW, Ekstrand J, Junge A, et al. Consensus statement on injury definitions and data collection procedures in studies of football (soccer) injuries. *Br J Sports Med* 2006;40(3):193-201. doi: 10.1136/bjsm.2005.025270.

32. Mueller-Wohlfahrt HW, Haensel L, Mithoefer K, et al. Terminology and classification of muscle injuries in sport: the Munich consensus statement. *Br J Sports Med* 2013;47(6):342-50. doi: 10.1136/bjsports-2012-091448.

33. Ekstrand J, Askling C, Magnusson H, et al. Return to play after thigh muscle injury in elite football players: implementation and validation of the Munich muscle injury classification. *Br J Sports Med* 2013;47(12):769-74. doi: 10.1136/bjsports-2012-092092.

34. Hsieh FY, Block DA, Larsen MD. A simple method of sample size calculation fro linear and logistic regression. *Stat Med* 1998;17(14):1623-34.

35. Coppieters M, Stappaerts K, Janssens K, et al. Reliability of detecting 'onset of pain' and 'submaximal pain' during neural provocation testing of the upper quadrant. *Physiother Res Int* 2002;7(4):146-56.

36. Roach S, San Juan JG, Suprak DN, et al. Concurrent validity of digital inclinometer and universal goniometer assessing passive hip mobility in healthy subjects. *The Int J of Sports Phys Ther* 2013;8(5):680-88.

37. Clapis PA, Davis SM, Davis RO. Reliability of inclinometer and goniometric measurements of hip extension flexibility using the modified Thomas test. *Phys Theory Pract* 2008;24(2):135-41.

For peer review only

38. Gabbe BJ, Bennell KL, Wajswelner H, et al. Reliability of common lower extremity musculoskeletal screening tests. *Phys Ther Sport* 2004;5(2):90-97. doi: 10.1016/s1466-853x(04)00022-7.

39. Williams CM, Caserta AJ, Haines TP. The TiltMeter app is a novel and accurate measurement tool for the weight bearing lunge test. *J Sci Med Sport* 2013;16(5):392-95. doi: 10.1016/j.jsams.2013.02.001.

40. Munteanu SE, Strawhorn AB, Landorf KB, et al. A weightbearing technique for the measurement of ankle joint dorsiflexion with the knee extended is reliable. *J Sci Med Sport* 2009;12(1):54-59. doi: 10.1016/j.jsams.2007.06.009.

41. Redden J, Stokes K, Williams S. Establishing the Reliability and Limits of Meaningful Change of Lower Limb Strength and Power Measures during Seated Leg Press in Elite Soccer Players. *J Sports Sci Med* 2018;17:539-46.

42. Slinde F, Suber C, Suber L, et al. Test–Retest Reliability Of Three Different Countermovement Jumping Tests. *J Strength Cond Res* 2008;22(2):640-43.

43. Jaric S. Muscle Strength Testing. Use of Normalisation for Body Size. *Sports Med* 2002;32(10):615-31.

44. Lunt M. nscore Manchester University of Manchester; 2007 [Available from: http://personalpages.manchester.ac.uk/staff/mark.lunt.

45. Sterne JAC, White IR, Carlin J, et al. Multiple imputation for missing data in epidemiological and clinical research: potential and pitfalls. *Br Med J* 2009;338:b2393. doi: DOI: 10.1136/bmj.b2393.

46. Marshall A, Altman DG, Holder RL, et al. Combining estimates of interest in prognostic modelling studies after multiple imputation: current practice and guidelines. *BMC Med Res Methodol* 2009;9:57. doi: 10.1186/1471-2288-9-57.

47. Bland M. An Introduction to Medical Statistics. 4th ed. New York: Oxford University Press 2015.

48. Royston P, Ambler G, Sauerbrei W. The Use of Fractional Polynomials to Model Continuous Risk Variables in Epidemiology. *Int J Epidemiol* 1999;28:964-74.

49. Morris TP, White IR, Carpenter JR, et al. Combining fractional polynomial model building with multiple imputation. *Stat Med* 2015;34(25):3298-317. doi: 10.1002/sim.6553.

50. Hayden JA, Cote P, Steenstra LA, et al. Identifying phases of investigations helps planning, appraising and applying the results of explanatory prognosis studies. *J Clin Epidemiol* 2008;61:552-60.

For peer review only

51. Bansal A, Heagerty PJ. A comparison of landmark methods and time-dependent ROC methods to evaluate the time-varying performance of prognostic markers for survival outcomes. *Diagn Progn Res* 2019;3:14. doi: 10.1186/s41512-019-0057-6.

52. Meeuwisse W, Tyreman H, Hagel B, et al. A Dynamic Model of Etiology in Sport Injury: The Recursive Nature of Risk and Causation. *Clin J Sports Med* 2007;17(3):215-19.

53. Riley RD, Sauerbrei W, Altman DG. Prognostic markers in cancer: the evolution of evidence from single studies to meta-analysis, and beyond. *Br J Cancer* 2009;100(8):1219-29. doi: 10.1038/sj.bjc.6604999.

For peer review only

CAPTIONS FOR FIGURE 1:

Figure 1: Participant flow chart

Key: I-IMI=index indirect muscle injury; n=number of participants

For peer review only - http://bmjopen.bmj.com/site/about/guidelines.xhtml

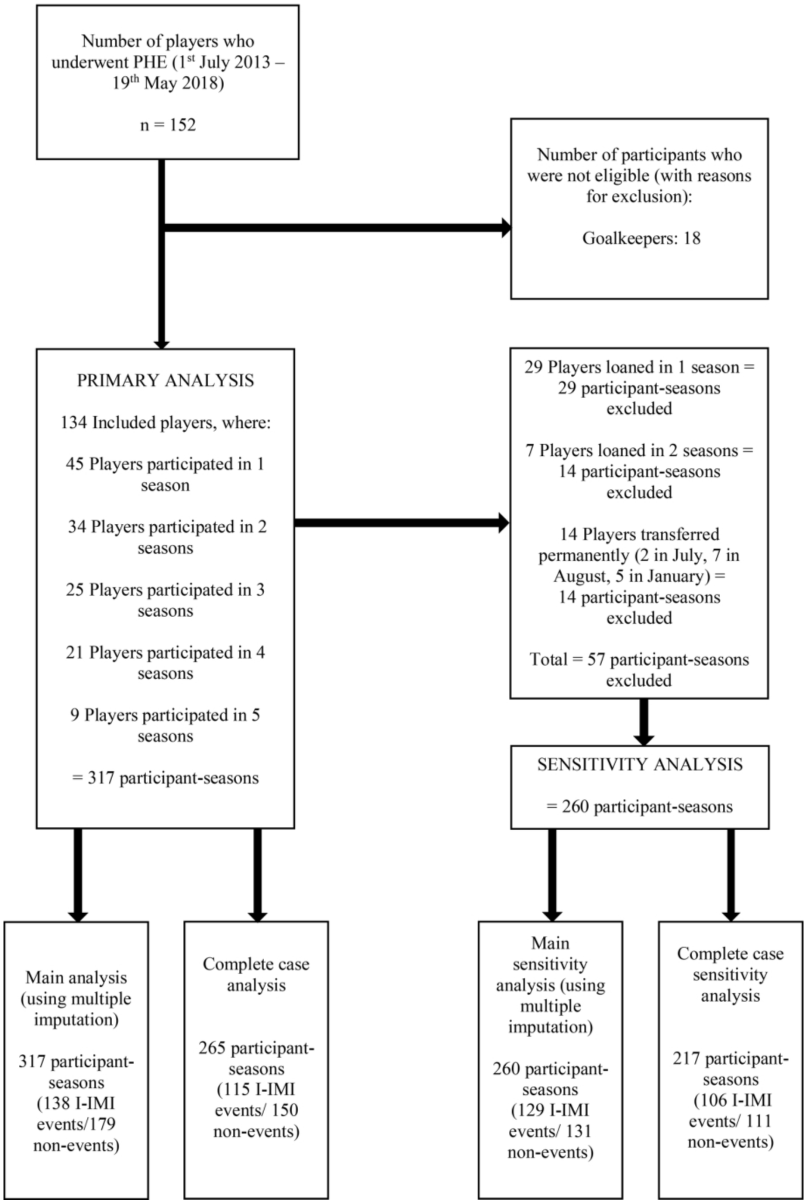

Figure 1: Participant flow chart

Key: I-IMI=index indirect muscle injury; n=number of participants

133x199mm (300 x 300 DPI)

Does preseason screening provide a source of potential prognostic factors for indirect muscle injuries in elite football (soccer) players? An exploratory analysis using routinely-collected periodic health examination data

Hughes, T., Riley, R.D., Callaghan, M.J. and Sergeant, J.C. (2021)

Supplementary file 1: Sample size calculation

For peer review only

PASS 2019, v19.0.2 13/11/2019 14:40:18 1

Tests for the Odds Ratio in Logistic Regression with One Normal X and Other Xs (Wald Test)

Numeric Results

Power	N	P0	P1	Odds Ratio	R Squared	Alpha	Beta
0.79904	314	0.430	0.542	1.570	0.500	0.050	0.20096

References

Hsieh, F.Y., Block, D.A., and Larsen, M.D. 1998. 'A Simple Method of Sample Size Calculation for Linear and
Logistic Regression', Statistics in Medicine, Volume 17, pages 1623-1634.

Report Definitions

Power is the probability of rejecting a false null hypothesis. It should be close to one.
N is the size of the sample drawn from the population.
P0 is the response probability at the mean of X.
P1 is the response probability when X is increased to one standard deviation above the mean.
Odds Ratio is the odds ratio when P1 is on top. That is, it is [P1/(1-P1)]/[P0/(1-P0)].
R-Squared is the R2 achieved when X is regressed on the other independent variables in the regression.
Alpha is the probability of rejecting a true null hypothesis.
Beta is the probability of accepting a false null hypothesis.

Summary Statements

A logistic regression of a binary response variable (Y) on a continuous, normally distributed variable (X) with a sample size of 314 observations achieves 80% power at a 0.050 significance level to detect a change in Prob(Y=1) from the value of 0.430 at the mean of X to 0.542 when X is increased to one standard deviation above the mean. This change corresponds to an odds ratio of 1.570. An adjustment was made since a multiple regression of the independent variable of interest on the other independent variables in the logistic regression obtained an R-Squared of 0.500.

Dropout-Inflated Sample Size

Dropout Rate	Sample Size N	Dropout-Inflated Enrollment Sample Size N'	Expected Number of Dropouts D
20%	314	393	79

Definitions

Dropout Rate (DR) is the percentage of subjects (or items) that are expected to be lost at random during the
course of the study and for whom no response data will be collected (i.e. will be treated as "missing").
N is the evaluable sample size at which power is computed. If N subjects are evaluated out of the N' subjects
that are enrolled in the study, the design will achieve the stated power.
N' is the total number of subjects that should be enrolled in the study in order to end up with N

For peer review only

evaluable
subjects, based on the assumed dropout rate. After solving for N, N' is calculated by inflating N using the
formula N' = N / (1 - DR), with N' always rounded up. (See Julious, S.A. (2010) pages 52-53, or Chow, S.C.,
Shao, J., Wang, H., and Lokhnygina, Y. (2018) pages 32-33.)
D is the expected number of dropouts. D = N' - N.

For peer review only

Tests for the Odds Ratio in Logistic Regression with One Normal X and Other Xs (Wald Test)

Procedure Input Settings

Autosave Inactive

Design Tab

Solve For:	Sample Size
Alternative Hypothesis:	Two-Sided
Power:	0.80
Alpha:	0.05
P0 (Baseline Probability that Y=1):	0.43
Use P1 or Odds Ratio:	Odds Ratio
Odds Ratio (Odds1/Odds0):	1.57
R-Squared of X1 with Other X's:	0.5

For peer review only

Does preseason screening provide a source of potential prognostic factors for indirect muscle injuries in elite football (soccer) players? An exploratory analysis using routinely-collected periodic health examination data

Hughes, T., Riley, R.D., Callaghan, M.J. and Sergeant, J.C. (2021)

Supplementary file 2: Candidate prognostic factors that were excluded

For peer review only

Type of prognostic factor	Candidate Prognostic Factor	Composite variable created	Measurement unit	Measurement method	Data type	Reason for elimination
Anthropometric	Body fat	No	Percentage	Skin calipers	Continuous	Missing data > 15%
Musculoskeletal test	Quadriceps muscle length difference*	Yes	Degrees	Ely's test using digital inclinometer	Continuous	Intra-rater ICC = 0.69[1] Inter-rater ICC = 0.66. [1]
	Mean quadriceps muscle length**	Yes	Degrees	Ely's test using goniometer	Continuous	Intra-rater ICC = 0.69[1] Inter-rater ICC = 0.66. [1]
	Toe touch in standing	No	Centimetres	Fingertips to floor distance	Continuous	Missing data > 15%
	Sacroiliac joint kinematic function	No	Subjective score	Gillets test	Categorical	Missing data > 15%
Functional movement/balance tests	Y Balance Test – anterior translation difference*	Yes	Centimetres	Y Balance Test	Continuous	Missing data >15%
	Y Balance Test – Mean anterior translation**	Yes	Centimetres	Y Balance Test	Continuous	Missing data >15%
	Y Balance Test – posteromedial translation difference*	Yes	Centimetres	Y Balance Test	Continuous	Missing data >15%
	Y Balance Test – Mean posteromedial translation**	Yes	Centimetres	Y Balance Test	Continuous	Missing data >15%
	Y Balance Test –posterolateral translation difference*	Yes	Centimetres	Y Balance Test	Continuous	Missing data >15%
	Y Balance Test – Mean posterolateral translation**	Yes	Centimetres	Y Balance Test	Continuous	Missing data >15%
	R relative tibial angles	No	Degrees	SLS measurement with Dorsavi Viperform IMU	Continuous	Within-session ICCs=0.27-0.75 Between-session ICCs = 0.55-0.77[2]
	L relative tibial angles (left leg)	No	Degrees	SLS measurement with Dorsavi Viperform IMU	Continuous	Within-session ICCs= 0.27-0.75 Between-session ICCs = 0.55-0.77[2]
Strength/power tests	Upper body peak power difference*	Yes	Normalised watts per kilo ($W/kg^{-0.67}$)	Double horizontal press using a Keiser Chest Press Air 350 machine	Continuous	Missing data > 15%
	Mean upper body peak power**	Yes	Normalised watts per kilo ($W/kg^{-0.67}$)	Double horizontal press using a Keiser Chest Press Air 350 machine	Continuous	Missing data > 15%

*Key: PHE=periodic health examination; WBL=weight bearing lunge; CMJ=countermovement jump; PROM=passive range of movement; ICC=intraclass correlation coefficient; SLR= straight leg raise; SLS = single leg squat; BMI= body mass index; f=force; W= watts; (note that $W/kg^{-0.67}$ has a scaling factor to normalise power to body mass) Kg=kilos; IMU= inertial measurement units; m = mass; Note: composite factors are identified in the table with * denoting between limb differences and **denoting combined mean values of both limbs.*

For peer review only

References:

1. Peeler J, Anderson JE. Reliability of the Ely's test for assessing rectus femoris muscle flexibility and joint range of motion. *J Orth Res* 2008;26(6):793-9.

2. Hughes T, Jones RK, Starbuck C, et al. Are tibial angles measured with inertial sensors useful surrogates for frontal plane projection angles measured using 2-dimensional video analysis during single leg squat tasks? A reliability and agreement study in elite football (soccer) players. *J Electromyogr Kinesiol* 2019;44:21-30. doi: 10.1016/j.jelekin.2018.11.005

Does preseason screening provide a source of potential prognostic factors for indirect muscle injuries in elite football (soccer) players? An exploratory analysis using routinely-collected periodic health examination data

Hughes, T., Riley, R.D., Callaghan, M.J. and Sergeant, J.C. (2021)

Supplementary file 3: Anthropometric parameters and all included candidate prognostic factor characteristics for participants included in the sensitivity analyses

For peer review only

Characteristic/candidate prognostic factors		*Min*	*Lower quartile*	*Median*	*Upper quartile*	*Max.*	*Freq. (%) - if categorical*	*Missing values n (%)*
**Anthropometrics**								
1. Age at PHE (years)		16.01	17.64	19.20	24.30	39.59	-	0 (0)
2. Height (cm)		164.30	176.10	180.00	185.50	195.00	-	15 (5.77)
3. Weight (kg)		56.80	68.09	73.10	80.00	94.00	-	16 (6.15)
**Past medical history**								
Within 3 years prior to PHE, freq. of:								
4. foot/ankle injuries		0.00	0.00	1.00	2.00	7.00	-	0 (0)
5. Hip/groin injuries		0.00	0.00	0.00	1.00	5.00	-	
6. Knee injuries		0.00	0.00	0.00	1.00	3.00	-	0 (0)
7. Shoulder injuries		0.00	0.00	0.00	0.00	2.00	-	0 (0)
8. lumbar spine injuries		0.00	0.00	0.00	0.00	3.00	-	0 (0)
9. iliopsoas IMIs		0.00	0.00	0.00	0.00	2.00	-	0 (0)
10. hip adductor IMIs		0.00	0.00	0.00	0.00	3.00	-	0 (0)
11.hamstring IMIs		0.00	0.00	0.00	1.00	5.00	-	0 (0)
12. quadriceps IMIs		0.00	0.00	0.00	0.00	3.00	-	0 (0)
13. calf IMIs		0.00	0.00	0.00	0.00	4.00	-	0 (0)
**Within 3 years prior to PHE, most recent:**								
14. foot/ankle injury	*Never*	-	-	-	-	-	120 (46.15)	0 (0)
	< 6 months	-	-	-	-	-	37 (14.23)	0 (0)
	6-12 months	-	-	-	-	-	29 (11.15)	0 (0)
	>12 months	-	-	-	-	-	74 (28.46)	0 (0)
15. Hip/groin injury	*Never*	-	-	-	-	-	176 (67.69)	0 (0)
	< 6 months	-	-	-	-	-	21 (8.08)	0 (0)
	6-12 months	-	-	-	-	-	20 (7.69)	0 (0)
	>12 months	-	-	-	-	-	43 (16.54)	0 (0)
16. Knee injury	*Never*	-	-	-	-	-	165 (63.46)	0 (0)
	< 6 months	-	-	-	-	-	15 (5.77)	0 (0)
	6-12 months	-	-	-	-	-	24 (9.23)	0 (0)
	>12 months	-	-	-	-	-	56 (21.54)	0 (0)
17. Shoulder injury	*Never*	-	-	-	-	-	240 (92.31)	0 (0)
	< 6 months	-	-	-	-	-	6 (2.31)	0 (0)
	6-12 months	-	-	-	-	-	4 (1.54)	0 (0)
	>12 months	-	-	-	-	-	10 (3.85)	0 (0)
18. lumbar spine injury	*Never*	-	-	-	-	-	210 (80.77)	0 (0)
	< 6 months	-	-	-	-	-	8 (3.08)	0 (0)
	6-12 months	-	-	-	-	-	7 (2.69)	0 (0)
	>12 months	-	-	-	-	-	35 (13.46)	0 (0)
19. iliopsoas IMI	*Never*	-	-	-	-	-	237 (91.15)	0 (0)
	< 6 months	-	-	-	-	-	2 (0.77)	0 (0)
	6-12 months	-	-	-	-	-	7 (2.69)	0 (0)
	>12 months	-	-	-	-	-	14 (5.38)	0 (0)
20. hip adductor IMI	*Never*	-	-	-	-	-	214 (82.31)	0 (0)
	< 6 months	-	-	-	-	-	17 (6.54)	0 (0)
	6-12 months	-	-	-	-	-	10 (3.85)	0 (0)
	>12 months	-	-	-	-	-	19 (7.31)	0 (0)
21.hamstring muscle IMI	*Never*	-	-	-	-	-	193 (74.23)	0 (0)
	< 6 months	-	-	-	-	-	8 (3.08)	0 (0)
	6-12 months	-	-	-	-	-	23 (8.85)	0 (0)
	>12 months	-	-	-	-	-	36 (13.85)	0 (0)
22. quadriceps IMI	*Never*	-	-	-	-	-	165 (63.46)	0 (0)
	< 6 months	-	-	-	-	-	5 (1.92)	0 (0)
	6-12 months	-	-	-	-	-	12 (4.62)	0 (0)
	>12 months	-	-	-	-	-	24 (9.23)	0 (0)
23. calf IMI	*Never*	-	-	-	-	-	230 (88.46)	0 (0)
	< 6 months	-	-	-	-	-	10 (3.85)	0 (0)
	6-12 months	-	-	-	-	-	5 (1.92)	0 (0)
	>12 months	-	-	-	-	-	15 (5.77)	0 (0)
**Musculoskeletal Examination**								
24. Mean PROM hip IR (deg.)		9.50	22.00	32.50	40.00	55.00	-	15 (5.77)
25. Mean PROM hip ER (deg.)		17.50	33.70	39.00	43.50	62.00	-	15 (5.77)
26. Mean hip flexor length (deg.)		-7.00	3.00	8.68	15.00	55.00	-	18 (6.92)
27. Mean hamstring/neural mobility length (deg.)		45.00	83.00	90.00	90.00	102.00	-	15 (5.77)
28. Mean calf muscle length (deg.)		9.50	25.65	30.50	37.00	57.50	-	15 (5.77)
**Strength/Power Tests**								
29: Max. leg extension power difference ($W/kg^{-0.67}$)		-11.94	-1.47	0.43	1.91	15.26	-	39 (15.00)
30: Mean of max. leg extension power ($W/kg^{-0.67}$)		23.01	41.67	46.40	51.10	78.70	-	39 (15.00)
31: Max. leg extension velocity difference ($m.s^{-1}$)		-0.34	-0.05	0.02	0.11	0.40	-	35 (13.46)
32: Mean of max. leg extension velocity ($m.s^{-1}$)		1.02	1.68	1.84	1.98	2.35	-	35 (13.46)
33: Max leg extension force difference ($N/kg^{-0.67}$)		-83.59	-5.74	-1.07	4.39	55.41	-	39 (15.00)
34: Mean of max. leg extension force ($N/kg^{-0.67}$)		50.19	90.85	101.14	112.02	217.96	-	39 (15.00)

For peer review only

For peer review only - http://bmjopen.bmj.com/site/about/guidelines.xhtml

35: CMJ Force per kg of body mass	20.50	23.30	25.18	27.90	39.20	-	35 (13.46)
36: CMJ height (cm)	28.70	37.20	39.90	43.10	58.00	-	35 (13.46)

Key: PHE= periodic health examination; I-IMI=index indirect muscle injury; IMI= indirect muscle injury; min = minimum; max = maximum; n = observations; Freq= frequency; WBL=weight bearing lunge; CMJ=countermovement jump; PROM=passive range of movement; deg. = degrees; SLR= straight leg raise; BMI= body mass index; kg/m^2 = kilograms/body height (metres) squared; *cm = centimetres; Kg=kilograms; Cont.=continuous; dis./cont.= discrete treated as continuous; cat.= categorical. Note that for the Musculoskeletal Examination and Strength/Power factors, positive values indicate greater left limb values compared to right limb values; negative values indicate greater right limb*

For peer review only

Does preseason screening provide a source of potential prognostic factors for indirect muscle injuries in elite football (soccer) players? An exploratory analysis using routinely-collected periodic health examination data

Hughes, T., Riley, R.D., Callaghan, M.J. and Sergeant, J.C. (2021)

Supplementary file 4: Graph to show the distribution of observed and imputed values for continuous variables

For peer review only

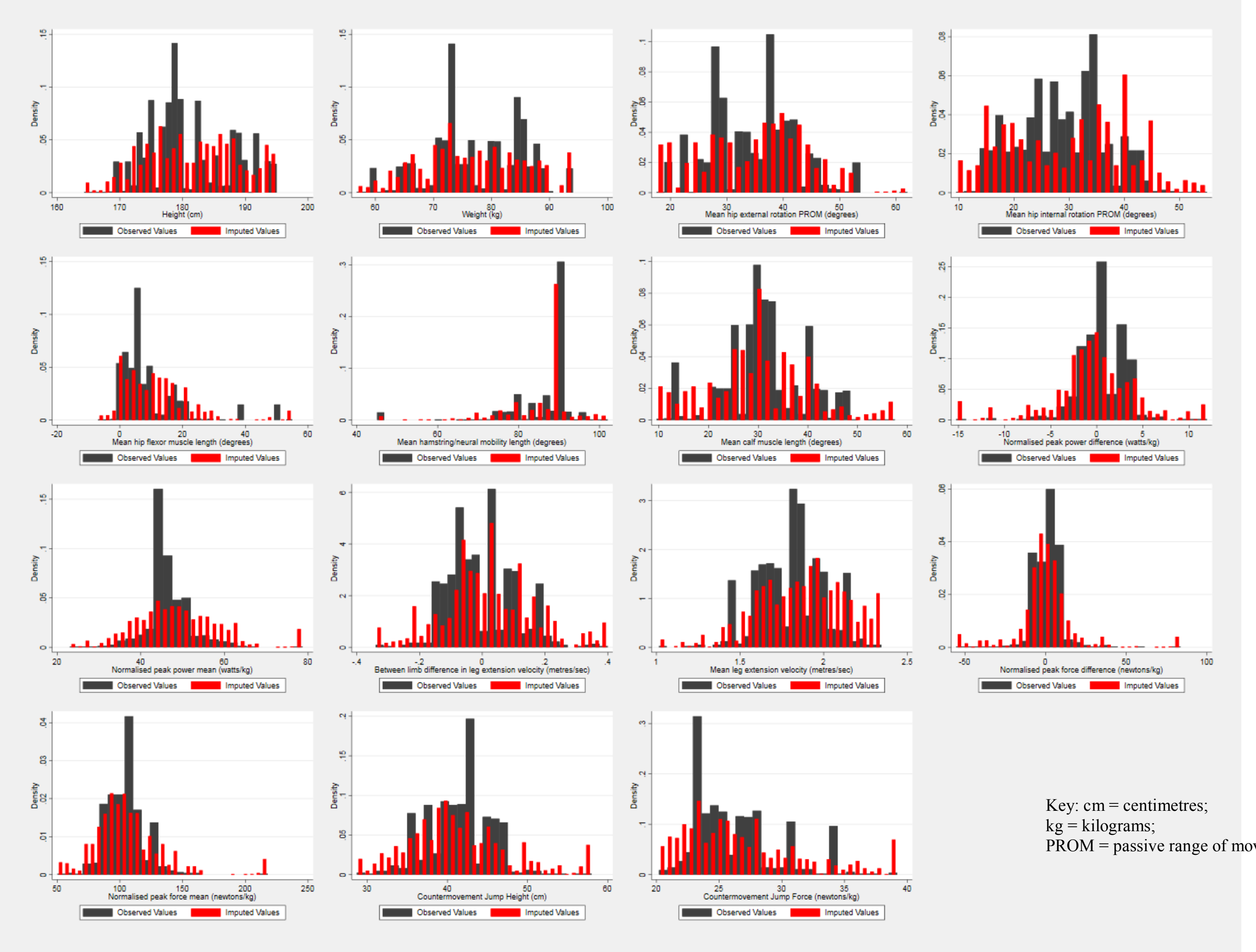

Key: cm = centimetres;
kg = kilograms;
PROM = passive range of movement

Does preseason screening provide a source of potential prognostic factors for indirect muscle injuries in elite football (soccer) players? An exploratory analysis using routinely-collected periodic health examination data

Hughes, T., Riley, R.D., Callaghan, M.J. and Sergeant, J.C. (2021)

Supplementary file 5: Results of univariable and multivariable analyses – Primary complete case analysis

For peer review only

	Univariable (unadjusted)				Multivariable (adjusted for age, height, weight)			
**Candidate PF & Type**	OR	95% CI	P	Best model fit	OR	95% CI	P	Best model fit
**Anthropometric (Adjustment Factors):**								
1: Age (years)	**1.12**	**1.06 to 1.18**	**<0.001**	**Linear**	**1.11**	**1.04 to 1.18**	**<0.001**	**Linear**
2: Height (cm)	1.03	0.99 to 1.06	0.18	-	1.02	0.97 to 1.07	0.50	-
3: Weight (kg)	1.03	0.99 to 1.06	0.07	-	0.99	0.95 to 1.04	0.81	-
**Within 3 years preceding PHE, frequency of:**								
4: foot/ankle injuries	1.04	0.87 to 1.23	0.68	-	1.04	0.86 to 1.25	0.70	-
5: hip/groin injuries	1.16	0.90 to 1.51	0.25	-	1.29	0.98 to 1.70	0.07	-
6: knee injuries	0.96	0.72 to 1.29	0.81	-	1.00	0.74 to 1.35	0.98	-
7: shoulder injuries	2.38	0.98 to 5.75	0.05	-	1.77	0.68 to 4.59	0.24	-
8: lumbar spine injuries	0.88	0.60 to 1.29	0.50	-	1.03	0.68 to 1.55	0.89	-
9: iliopsoas IMIs	0.73	0.38 to 1.43	0.37	-	0.88	0.44 to 1.76	0.72	-
10: hip adductor IMIs	1.38	0.92 to 2.09	0.12	-	1.22	0.78 to 1.90	0.39	-
11: hamstring IMIs	**1.56**	**1.17 to 2.09**	**<0.001**	**Linear**	1.30	0.96 to 1.77	0.09	-
12: quadriceps IMIs	1.08	0.67 to 1.73	0.75	-	1.00	0.61 to 1.63	0.99	-
13: calf IMIs	**1.80**	**1.09 to 2.97**	**0.02**	**Linear**	1.30	0.74 to 2.29	0.35	-
**Within 3 years preceding PHE, most recent:**								
14: foot/ankle injury (never)	ref	ref	ref	-	ref	ref	ref	-
14: foot/ankle injury (<6 months)	1.27	0.64 to 2.53	0.49	-	1.36	0.66 to 2.79	0.40	-
14: foot/ankle injury (6-12 months)	1.16	0.54 to 2.46	0.71	-	1.09	0.49 to 2.45	0.83	-
14: foot/ankle injury (>12 months)	1.27	0.75 to 2.13	0.37	-	1.05	0.60 to 1.84	0.86	-
15: hip/groin injury (never)	ref	ref	ref	-	ref	ref	ref	-
15: hip/groin injury (<6 months)	1.05	0.44 to 2.49	0.92	-	1.45	0.52 to 4.00	0.48	-
15: hip/groin injury (6-12 months)	0.59	0.23 to 1.50	0.27	-	1.17	0.35 to 3.84	0.80	-
15: hip/groin injury (>12months)	1.58	0.86 to 2.87	0.14	-	1.14	0.45 to 2.88	0.78	-
16: knee injury (never)	ref	ref	ref	-	ref	ref	ref	-
16: knee injury (<6 months)	1.15	0.40 to 3.28	0.80	-	1.11	0.33 to 3.72	0.87	-
16: knee injury (6-12 months)	1.23	0.58 to 2.62	0.60	-	1.24	0.57 to 2.72	0.59	-
16: knee injury (>12months)	0.93	0.53 to 1.60	0.79	-	1.10	0.61 to 1.98	0.76	-
17: shoulder injury (never)	ref	ref	ref	-	ref	ref	ref	-
17: shoulder injury (<6 months)	2.75	0.50 to 15.26	0.25	-	2.07	0.33 to 12.90	0.44	-
17: shoulder injury (6-12 months)	1.38	0.19 to 9.90	0.75	-	1.12	0.15 to 8.37	0.91	-
17: shoulder injury (>12months)	3.21	0.81 to 12.66	0.10	-	2.38	0.59 to 9.70	0.23	-
18: lumbar spine injury (never)	ref	ref	ref	-	ref	ref	ref	-
18: lumbar spine injury (<6 months)	1.24	0.30 to 5.05	0.77	-	2.66	0.57 to 12.51	0.22	-
18: lumbar spine injury (6-12 months)	0.62	0.15 to 2.52	0.50	-	0.72	0.17 to 3.03	0.66	-
18: lumbar spine injury (>12months)	0.70	0.34 to 1.44	0.33	-	0.90	0.42 to 1.94	0.79	-
19: iliopsoas IMI (never)	ref	ref	ref	-	ref	ref	ref	-
19: iliopsoas IMI (<6 months)	1.24	0.08 to 20.05	0.88	-	1.29	0.08 to 21.58	0.86	-
19: iliopsoas IMI (6-12 months)	0.62	0.15 to 2.53	0.51	-	0.80	0.19 to 3.33	0.76	-
19: iliopsoas IMI (>12months)	0.57	0.21 to 1.60	0.27	-	0.70	0.25 to 1.96	0.50	-
20: hip adductor IMI (never)	ref	ref	ref	-	ref	ref	ref	-
20: hip adductor IMI (<6 months)	1.37	0.53 to 3.56	0.52	-	1.45	0.52 to 4.00	0.48	-
20: hip adductor IMI (6-12 months)	1.37	0.43 to 4.36	0.60	-	1.17	0.35 to 3.84	0.80	-
20: hip adductor IMI (>12months)	1.37	0.59 to 3.16	0.46	-	1.14	0.45 to 2.88	0.78	-
21: hamstring IMI (never)	ref	ref	ref	-	ref	ref	ref	-
21: hamstring IMI (<6 months)	2.84	0.81 to 9.99	0.10	-	1.44	0.36 to 5.77	0.60	-
21: hamstring IMI (6-12 months)	1.42	0.66 to 3.06	0.37	-	1.25	0.55 to 2.86	0.60	-
21: hamstring IMI (>12months)	**2.95**	**1.51 to 5.73**	**<0.001**	**Linear**	2.10	1.01 to 4.34	0.05	-
22: quadriceps IMI (never)	ref	ref	ref	-	ref	ref	ref	-
22: quadriceps IMI (<6 months)	1.74	0.38 to 7.91	0.48	-	1.52	0.32 to 7.20	0.60	-
22: quadriceps IMI (6-12 months)	0.58	0.17 to 1.93	0.37	-	0.72	0.21 to 2.50	0.60	-
22: quadriceps IMI (>12months)	1.14	0.53 to 2.42	0.74	-	0.85	0.37 to 1.94	0.69	-
23: calf IMI (never)	ref	ref	ref	-	ref	ref	ref	-
23: calf IMI (<6 months)	3.78	0.98 to 14.56	0.05	-	2.80	0.68 to 11.47	0.15	-
23: calf IMI (6-12 months)	7.09	0.82 to 61.51	0.08	-	3.36	0.35 to 32.71	0.30	-
23: calf IMI (>12months)	1.26	0.56 to 0.89	0.64	-	0.87	0.29 to 2.62	0.80	-
**Musculoskeletal:**								
24. Mean PROM hip IR (deg.)	**0.97**	**0.95 to 0.99**	**0.01**	**Linear**	**0.97**	**0.95 to 1.00**	**0.04**	**Linear**
25. Mean PROM hip ER (deg.)	0.97	0.94 to 1.00	0.08	-	0.98	0.95 to 1.01	0.26	-
26. Mean hip flexor length (deg.)	1.01	0.98 to 1.04	0.50	-	1.01	0.98 to 1.04	0.50	-

For peer review only

27. Mean hamstring/neural mobility length (deg.)	0.99	0.96 to 1.02	0.53	-	0.98	0.94 to 1.01	0.21	-
28. Mean calf muscle length (deg.)	1.00	0.97 to 1.02	0.73	-	0.99	0.97 to 1.02	0.64	-
**Strength/Power:**								
29: Max. leg extension power difference ($W/kg^{-0.67}$)	1.00	0.93 to 1.07	0.92	-	0.99	0.92 to 1.07	0.78	-
30: Mean of max. leg extension power ($W/kg^{-0.67}$)	1.02	0.99 to 1.05	0.30	-	1.01	0.97 to 1.04	0.76	-
31: Max. leg extension velocity difference ($m.s^{-1}$)	2.14	0.30 to 15.00	0.45	-	2.61	0.34 to 19.87	0.36	-
32: Mean of max. leg extension velocity ($m.s^{-1}$)	1.86	0.63 to 5.53	0.26	-	1.44	0.46 to 4.53	0.53	-
33: Max leg extension force difference ($N/kg^{-0.67}$)	1.00	0.98 to 1.02	0.66	-	1.00	0.98 to 1.02	0.67	-
34: Mean of max. leg extension force ($N/kg^{-0.67}$)	1.00	0.99 to 1.01	0.96	-	1.00	0.98 to 1.01	0.69	-
35: CMJ Force per kg of body mass (N/kg)	0.99	0.92 to 1.06	0.73	-	0.99	0.92 to 1.06	0.82	-
36: CMJ height (cm)	1.03	0.97 to 1.07	0.34	-	1.01	0.96 to 1.07	0.59	-

Key: PHE= periodic health examination; PF= prognostic factor; OR=odds ratio; CI=confidence interval; ref=reference category; I-IMI=index indirect muscle injury; IMI= indirect muscle injury; Freq= frequency; WBL=weight bearing lunge; CMJ=countermovement jump; PROM=passive range of movement; deg. = degrees; SLR= straight leg raise; BMI= body mass index; kg/m^2 = kilograms/body height (metres) squared; *cm = centimetres; Kg=kilograms; W= watts (note: $W/kg^{-0.67}$ has a scaling factor to normalise force to body mass[42]); N= newtons (note: $N/kg^{-0.67}$ has a scaling factor to normalise force to body mass);max.=maximum; m.s.= metres per second; cm = centimetres; Kg=kilograms; - = not applicable.*
Note: *ORs are expressed per one-unit increase for all continuous factors, and according to category for all categorical factors; Factors in* ***bold*** *indicate significance at the 0.05 level.*

For peer review only

Does preseason screening provide a source of potential prognostic factors for indirect muscle injuries in elite football (soccer) players? An exploratory analysis using routinely-collected periodic health examination data

Hughes, T., Riley, R.D., Callaghan, M.J. and Sergeant, J.C. (2021)

Supplementary file 6: Results of univariable and multivariable analyses – Sensitivity analysis using imputed data

For peer review only

	Univariable (unadjusted)				Multivariable (adjusted for age, height, weight)			
Candidate PF & Type	OR	95% CI	P	Best model fit	OR	95% CI	P	Best model fit
**Anthropometric (Adjustment Factors):**								
1: Age (years)	**1.15**	1.08 to 1.22	**<0.001**	**Linear**	**1.14**	**1.06 to 1.22**	**<0.001**	**Linear**
2: Height (cm)	1.02	0.98 to 1.06	0.25	-	0.99	0.94 to 1.05	0.85	-
3: Weight (kg)	**1.05**	**1.02 to 1.08**	**0.01**	**Linear**	1.02	0.97 to 1.07	0.49	-
**Within 3 years preceding PHE, frequency of:**								
4: foot/ankle injuries	1.03	0.86 to 1.25	0.73	-	1.04	0.85 to 1.27	0.70	-
5: hip/groin injuries	1.09	0.83 to 1.43	0.54	-	1.23	0.92 to 1.64	0.16	-
6: knee injuries	0.91	0.67 to 1.23	0.54	-	0.92	0.67 to 1.26	0.59	-
7: shoulder injuries	1.91	0.80 to 4.59	0.15	-	1.34	0.53 to 3.39	0.54	-
8: lumbar spine injuries	0.78	0.53 to 1.16	0.22	-	0.99	0.66 to1.50	0.98	-
9: iliopsoas IMIs	0.74	0.36 to 1.50	0.40	-	0.86	0.41 to 1.81	0.70	-
10: hip adductor IMIs	1.46	0.93 to 2.30	0.10	-	1.14	0.70 to 1.87	0.60	-
11: hamstring IMIs	**2.39**	**1.55 to 3.69**	**<0.001**	**Linear**	**1.87**	**1.19 to 2.96**	**0.01**	**Linear**
12: quadriceps IMIs	1.12	0.67 to 1.86	0.66	-	1.09	0.64 to 1.87	0.75	-
13: calf IMIs	**2.17**	**1.13 to 4.16**	**0.02**	**Linear**	1.47	0.70 to 3.08	0.31	-
**Within 3 years preceding PHE, most recent:**								
14: foot/ankle injury (never)	ref	ref	ref	-	ref	ref	ref	-
14: foot/ankle injury (<6 months)	1.25	0.59 to 2.61	0.56	-	1.52	0.70 to 3.31	0.29	-
14: foot/ankle injury (6-12 months)	1.27	0.57 to 2.85	0.57	-	1.30	0.55 to 3.09	0.55	-
14: foot/ankle injury (>12 months)	1.39	0.78 to 2.49	0.27	-	1.26	0.67 to 2.34	0.47	-
15: hip/groin injury (never)	ref	ref	ref	-	ref	ref	ref	-
15: hip/groin injury (<6 months)	0.77	0.31 to 1.91	0.57	-	1.36	0.52 to 3.52	0.53	-
15: hip/groin injury (6-12 months)	0.55	0.21 to 1.45	0.23	-	0.57	0.20 to 1.58	0.28	-
15: hip/groin injury (>12months)	1.56	0.79 to 3.09	0.20	-	1.87	0.92 to 3.81	0.09	-
16: knee injury (never)	ref	ref	ref	-	ref	ref	ref	-
16: knee injury (<6 months)	0.89	0.31 to 2.55	0.82	-	0.87	0.28 to 2.67	0.81	-
16: knee injury (6-12 months)	1.20	0.51 to 2.82	0.68	-	1.15	0.47 to 2.83	0.76	-
16: knee injury (>12months)	0.94	0.51 to 1.72	0.85	-	1.01	0.54 to 1.91	0.97	-
17: shoulder injury (never)	ref	ref	ref	-	ref	ref	ref	-
17: shoulder injury (<6 months)	2.14	0.38 to 11.89	0.39	-	1.83	0.31 to 11.00	0.51	-
17: shoulder injury (6-12 months)	1.07	0.15 to 7.71	0.95	-	0.80	0.10 to 6.32	0.83	-
17: shoulder injury (>12months)	2.49	0.63 to 9.87	0.19	-	1.58	0.37 to 6.62	0.53	-
18: lumbar spine injury (never)	ref	ref	ref	-	ref	ref	ref	-
18: lumbar spine injury (<6 months)	0.93	0.23 to 3.80	0.92	-	1.70	0.40 to 7.18	0.47	-
18: lumbar spine injury (6-12 months)	0.69	0.15 to 3.18	0.64	-	0.86	0.18 to 4.18	0.85	-
18: lumbar spine injury (>12months)	0.55	0.26 to 1.14	0.11	-	0.80	0.37 to 1.73	0.57	-
19: iliopsoas IMI (never)	ref	ref	ref	-	ref	ref	ref	-
19: iliopsoas IMI (<6 months)	0.98	0.06 to 15.77	0.99	-	1.01	0.06 to 17.67	0.99	-
19: iliopsoas IMI (6-12 months)	0.39	0.07 to 2.05	0.27	-	0.54	0.10 to 2.92	0.48	-
19: iliopsoas IMI (>12months)	0.73	0.25 to 2.17	0.57	-	0.81	0.26 to 2.50	0.71	-
20: hip adductor IMI (never)	ref	ref	ref	-	ref	ref	ref	-
20: hip adductor IMI (<6 months)	1.24	0.46 to 3.32	0.68	-	1.00	0.35 to 2.83	0.99	-
20: hip adductor IMI (6-12 months)	1.65	0.45 to 6.00	0.45	-	1.24	0.32 to 4.84	0.75	-
20: hip adductor IMI (>12months)	1.88	0.71 to 4.96	0.20	-	1.36	0.47 to 3.90	0.57	-
21: hamstring IMI (never)	ref	ref	ref	-	ref	ref	ref	-
21: hamstring IMI (<6 months)	4.06	0.80 to 20.64	0.09	-	2.64	0.49 to 14.29	0.26	-
21: hamstring IMI (6-12 months)	1.76	0.74 to 4.21	0.20	-	1.42	0.58 to 3.52	0.44	-
21: hamstring IMI (>12months)	**4.74**	**2.05 to 10.93**	**<0.001**	**Linear**	**3.03**	**1.25 to 7.36**	**0.01**	**Linear**
22: quadriceps IMI (never)	**ref**	ref	**ref**	-	**ref**	**ref**	**ref**	**-**
22: quadriceps IMI (<6 months)	4.11	0.45 to 37.37	0.21	-	4.09	0.42 to 39.53	0.22	-
22: quadriceps IMI (6-12 months)	0.51	0.15 to 1.76	0.29	-	0.51	0.14 to 1.85	0.31	-
22: quadriceps IMI (>12months)	1.21	0.52 to 2.83	0.65	-	1.13	0.47 to 2.77	0.77	-
23: calf IMI (never)	ref	ref	ref	-	ref	ref	ref	-
23: calf IMI (<6 months)	4.44	0.92 to 21.36	0.06	-	3.62	0.71 to 18.47	0.12	-
23: calf IMI (>6 months)*	1.66	0.66 to 4.23	0.28	-	0.79	0.27 to 2.33	0.67	-
**Musculoskeletal:**								
24. Mean PROM hip IR (deg.)	**0.97**	**0.94 to 0.99**	**0.01**	**Linear**	0.98	0.95 to 1.01	0.12	-
25. Mean PROM hip ER (deg.)	**0.96**	**0.93 to 1.00**	**0.03**	**Linear**	0.99	0.95 to 1.02	0.46	-
26. Mean hip flexor length (deg.)	1.02	0.99 to 1.05	0.15	-	1.03	0.99 to 1.07	0.07	-
27. Mean hamstring/neural mobility length (deg.)	0.99	0.95 to 1.02	0.44	-	0.97	0.93 to 1.01	0.15	-
28. Mean calf muscle length (deg.)	0.99	0.96 to 1.02	0.45	-	0.99	0.96 to 1.02	0.34	-

For peer review only

**Strength/Power:**								
29: Max. leg extension power difference (W/kg$^{-0.67}$)	0.98	0.91 to 1.06	0.58	-	0.97	0.90 to 1.06	0.48	-
30: Mean of max. leg extension power (W/kg$^{-0.67}$)	1.03	0.99 to 1.06	0.14	-	1.00	0.97 to 1.04	0.77	-
31: Max. leg extension velocity difference (m.s^{-1})	2.28	0.28 to 18.29	0.44	-	3.24	0.37 to 28.60	0.29	-
32: Mean of max. leg extension velocity (m.s^{-1})	2.44	0.77 to 7.72	0.13	-	1.65	0.46 to 5.80	0.44	-
33: Max leg extension force difference (N/kg$^{-0.67}$)	0.99	0.97 to 1.01	0.50	-	0.99	0.98 to 1.01	0.44	-
34: Mean of max. leg extension force (N/kg$^{-0.67}$)	1.00	0.99 to 1.01	0.94	-	0.99	0.98 to 1.02	0.63	-
35: CMJ Force per kg of body mass (N/kg)	0.99	0.92 to 1.06	0.80	-	0.98	0.90 to 1.06	0.60	-
36: CMJ height (cm)	1.03	0.98 to 1.09	0.25	-	1.00	0.95 to 1.07	0.85	-

Key: PHE= periodic health examination; PF= prognostic factor; OR=odds ratio; CI=confidence interval; ref=reference category; I-IMI=index indirect muscle injury; IMI= indirect muscle injury; Freq= frequency; WBL=weight bearing lunge; CMJ=countermovement jump; PROM=passive range of movement; deg. = degrees; SLR= straight leg raise; BMI= body mass index; kg/m^2 = kilograms/body height (metres) squared; *cm = centimetres; Kg=kilograms; W= watts (note: W/kg$^{-0.67}$ has a scaling factor to normalise force to body mass[42]); N= newtons (note: N/kg$^{-0.67}$ has a scaling factor to normalise force to body mass);max.=maximum; m.s.= metres per second; cm = centimetres; Kg=kilograms; - = not applicable; * indicates merged category (using calf IMI (6-12 months) and calf IMI (>12months). This was required due to the occurrence of perfect predictions in the Calf (6-12 months) category as a consequence of reduced sample size.* ***Note:*** *ORs are expressed per one-unit increase for all continuous factors, and according to category for all categorical factors; Factors in* ***bold*** *indicate significance at the 0.05 level.*

For peer review only

Does preseason screening provide a source of potential prognostic factors for indirect muscle injuries in elite football (soccer) players? An exploratory analysis using routinely-collected periodic health examination data

Hughes, T., Riley, R.D., Callaghan, M.J. and Sergeant, J.C. (2021)

Supplementary file 7: Results of univariable and multivariable analyses – Sensitivity analysis using complete case data

For peer review only

	Univariable (unadjusted)				Multivariable (adjusted for age, height, weight)			
**Candidate PF & Type**	OR	95% CI	P	Best model fit	OR	95% CI	P	Best model fit
**Anthropometric (Adjustment Factors):**								
1: Age (years)	**1.15**	1.08 to 1.22	**<0.00**	**Linear**	**1.14**	**1.64 to 1.22**	**<0.00**	**Linear**
2: Height (cm)	1.02	0.98 to 1.05	0.36	-	0.99	0.94 to 1.05	0.73	-
3: Weight (kg)	**1.04**	**1.01 to 1.08**	**0.01**	**Linear**	1.01	0.96 to 1.06	0.60	-
**Within 3 years preceding PHE, frequency of:**								
4: foot/ankle injuries	1.03	0.86 to 1.25	0.73	-	1.05	0.85 to 1.30	0.63	-
5: hip/groin injuries	1.09	0.83 to 1.43	0.54	-	1.23	0.92 to 1.65	0.16	-
6: knee injuries	0.91	0.67 to 1.23	0.54	-	0.93	0.67 to 1.28	0.65	-
7: shoulder injuries	1.91	0.80 to 4.59	0.15	-	1.23	0.46 to 3.24	0.69	-
8: lumbar spine injuries	0.78	0.53 to 1.16	0.22	-	0.96	0.63 to 1.46	0.84	-
9: iliopsoas IMIs	0.74	0.36 to 1.50	0.40	-	0.86	0.41 to 1.79	0.68	-
10: hip adductor IMIs	1.46	0.93 to 2.30	0.10	-	1.18	0.71 to 1.95	0.53	-
11: hamstring IMIs	**2.39**	**1.60 to 3.69**	**<0.00**	**Linear**	**1.72**	**1.09 to 2.73**	**0.02**	**Linear**
12: quadriceps IMIs	1.12	0.67 to 1.86	0.66	-	1.05	0.61 to 1.82	0.90	-
13: calf IMIs	**2.17**	**1.13 to 4.16**	**0.02**	**Linear**	1.52	0.69 to 3.33	0.30	-
**Within 3 years preceding PHE, most recent:**								
14: foot/ankle injury (never)	ref	ref	ref	-	ref	ref	ref	-
14: foot/ankle injury (<6 months)	1.24	0.46 to 3.32	0.67	-	1.52	0.69 to 3.34	0.30	-
14: foot/ankle injury (6-12 months)	1.65	0.46 to 6.00	0.45	-	1.32	0.54 to 3.21	0.54	-
14: foot/ankle injury (>12 months)	1.90	0.71 to 4.97	0.20	-	1.12	0.58 to 2.14	0.74	-
15: hip/groin injury (never)	ref	ref	ref	-	ref	ref	ref	-
15: hip/groin injury (<6 months)	0.77	0.31 to 1.91	0.57	-	1.30	0.50 to 3.38	0.60	-
15: hip/groin injury (6-12 months)	0.55	0.31 to 1.91	0.23	-	0.72	0.25 to 2.05	0.54	-
15: hip/groin injury (>12months)	1.56	0.79 to 3.09	0.20	-	1.79	0.87 to 3.69	0.11	-
16: knee injury (never)	ref	ref	ref	-	ref	ref	ref	-
16: knee injury (<6 months)	0.88	0.31 to 2.55	0.82	-	0.92	0.27 to 3.12	0.89	-
16: knee injury (6-12 months)	1.20	0.51 to 2.82	0.68	-	1.14	0.46 to 2.82	0.77	-
16: knee injury (>12months)	0.94	0.51 to 1.72	0.85	-	1.10	0.57 to 2.12	0.77	-
17: shoulder injury (never)	ref	ref	ref	-	ref	ref	ref	-
17: shoulder injury (<6 months)	2.14	0.38 to 11.89	0.38	-	1.54	0.24 to 9.91	0.65	-
17: shoulder injury (6-12 months)	1.07	0.15 to 7.71	0.94	-	0.80	0.10 to 6.27	0.83	-
17: shoulder injury (>12months)	2.49	0.63 to 9.87	0.19	-	1.55	0.37 to 6.52	0.548	-
18: lumbar spine injury (never)	ref	ref	ref	-	ref	ref	ref	-
18: lumbar spine injury (<6 months)	0.93	0.23 to 3.80	0.92	-	2.20	0.46 to 10.40	0.32	-
18: lumbar spine injury (6-12 months)	0.69	0.15 to 3.18	0.91	-	0.84	0.17 to 4.06	0.83	-
18: lumbar spine injury (>12months)	0.55	0.26 to 1.14	0.11	-	0.75	0.34 to 1.66	0.48	-
19: iliopsoas IMI (never)	ref	ref	ref	-	ref	ref	ref	-
19: iliopsoas IMI (<6 months)	1.18	0.07 to 19.09	0.91	-	1.04	0.06 to 18.44	0.98	-
19: iliopsoas IMI (6-12 months)	0.47	0.09 to 2.48	0.38	-	0.53	0.10 to 2.85	0.46	-
19: iliopsoas IMI (>12months)	0.89	0.30 to 2.63	0.83	-	0.80	0.26 to 2.46	0.70	-
20: hip adductor IMI (never)	ref	ref	ref	-	ref	ref	Ref	-
20: hip adductor IMI (<6 months)	1.49	0.55 to 4.00	0.43	-	1.12	0.38 to 3.30	0.84	-
20: hip adductor IMI (6-12 months)	1.59	0.47 to 5.36	0.46	-	1.23	0.32 to 4.76	0.69	-
20: hip adductor IMI (>12months)	1.76	0.71 to 4.36	0.22	-	1.43	0.47 to 4.35	0.531	-
21: hamstring IMI (never)	ref	ref	ref	-	ref	ref	ref	-
21: hamstring IMI (<6 months)	4.06	0.80 to 20.63	0.09	-	1.64	0.27 to 9.99	0.59	-
21: hamstring IMI (6-12 months)	1.76	0.80 to 20.63	0.09	-	1.25	0.50 to 3.13	0.64	-
21: hamstring IMI (>12months)	**4.73**	**2.05 to 10.93**	**<0.00**	**Linear**	**2.94**	**1.15 to 7.53**	**0.03**	**Linear**
22: quadriceps IMI (never)	ref	ref	ref	-	ref	ref	ref	-
22: quadriceps IMI (<6 months)	4.11	0.45 to 37.37	0.21	-	4.03	0.41 to 39.22	0.23	-
22: quadriceps IMI (6-12 months)	0.51	0.15 to 1.76	0.29	-	0.66	0.18 to 2.39	0.52	-
22: quadriceps IMI (>12months)	1.21	0.52 to 2.83	0.65	-	0.89	0.35 to 2.28	0.81	-
23: calf IMI (never)	ref	ref	ref	-	ref	ref	ref	-
23: calf IMI (<6 months)	4.44	0.92 to 21.36	0.06	-	3.33	0.64 to 17.30	0.15	-
23: calf IMI (>6 months)*	1.66	0.66 to 4.23	0.28	-	0.88	0.29 to 2.76	0.84	-
**Musculoskeletal:**								
24. Mean PROM hip IR (deg.)	**0.97**	**0.94 to 0.99**	**0.01**	**Linear**	0.98	0.95 to 1.00	0.10	-
25. Mean PROM hip ER (deg.)	**0.96**	**0.93 to 0.99**	**0.02**	**Linear**	0.98	0.94 to 1.02	0.26	-
26. Mean hip flexor length (deg.)	1.02	0.99 to 1.05	0.21	-	1.03	0.99 to 1.06	0.13	-
27. Mean hamstring/neural mobility length (deg.)	0.98	0.95 to 1.02	0.40	-	0.96	0.92 to 1.00	0.06	-

For peer review only

28. Mean calf muscle length (deg.)	0.99	0.96 to 1.02	0.38	-	0.98	0.95 to 1.01	0.21	-
**Strength/Power:**								
29: Max. leg extension power difference (W/kg$^{-0.67}$)	0.98	0.90 to 1.06	0.63	-	0.97	0.89 to 1.06	0.59	-
30: Mean of max. leg extension power (W/kg$^{-0.67}$)	1.03	0.99 to 1.06	0.16	-	1.00	0.97 to 1.05	0.81	-
31: Max. leg extension velocity difference (m.s^{-1})	2.64	0.31 to 22.58	0.38	-	3.87	0.39 to 38.79	0.25	-
32: Mean of max. leg extension velocity (m.s^{-1})	2.49	0.74 to 8.32	0.14	-	1.60	0.42 to 6.09	0.49	-
33: Max leg extension force difference (N/kg$^{-0.67}$)	0.99	0.97 to 1.01	0.32	-	0.99	0.97 to 1.01	0.33	-
34: Mean of max. leg extension force (N/kg$^{-0.67}$)	1.00	0.99 to 1.01	0.98	-	1.00	0.98 to 1.01	0.59	-
35: CMJ Force per kg of body mass (N/kg)	0.98	0.91 to 1.05	0.58	-	0.97	0.89 to 1.05	0.47	-
36: CMJ height (cm)	1.03	0.97 to 1.09	0.35	-	1.00	0.95 to 1.07	0.86	-

Key: PHE= periodic health examination; PF= prognostic factor; OR=odds ratio; CI=confidence interval; ref=reference category; I-IMI=index indirect muscle injury; IMI= indirect muscle injury; Freq= frequency; WBL=weight bearing lunge; CMJ=countermovement jump; PROM=passive range of movement; deg. = degrees; SLR= straight leg raise; BMI= body mass index; kg/m^2 = kilograms/body height (metres) squared; *cm = centimetres; Kg=kilograms; W= watts (note: W/kg$^{-0.67}$ has a scaling factor to normalise force to body mass[42]); N= newtons (note: N/kg$^{-0.67}$ has a scaling factor to normalise force to body mass);max.=maximum; m.s.= metres per second; cm = centimetres; Kg=kilograms; - = not applicable; * indicates merged category (using calf IMI (6-12 months) and calf IMI (>12months). This was required due to the occurrence of perfect predictions in the Calf (6-12 months) category as a consequence of reduced sample size.* ***Note:*** *ORs are expressed per one-unit increase for all continuous factors, and according to category for all categorical factors; Factors in* ***bold*** *indicate significance at the 0.05 level.*

For peer review only

Does preseason screening provide a source of potential prognostic factors for indirect muscle injuries in elite football (soccer) players? An exploratory analysis using routinely-collected periodic health examination data

Hughes, T., Riley, R.D., Callaghan, M.J. and Sergeant, J.C. (2021)

Supplementary File 8: Comparison of candidate prognostic factors with statistically significant associations across the primary and sensitivity univariable analyses (using imputed and complete case datasets).

For peer review only

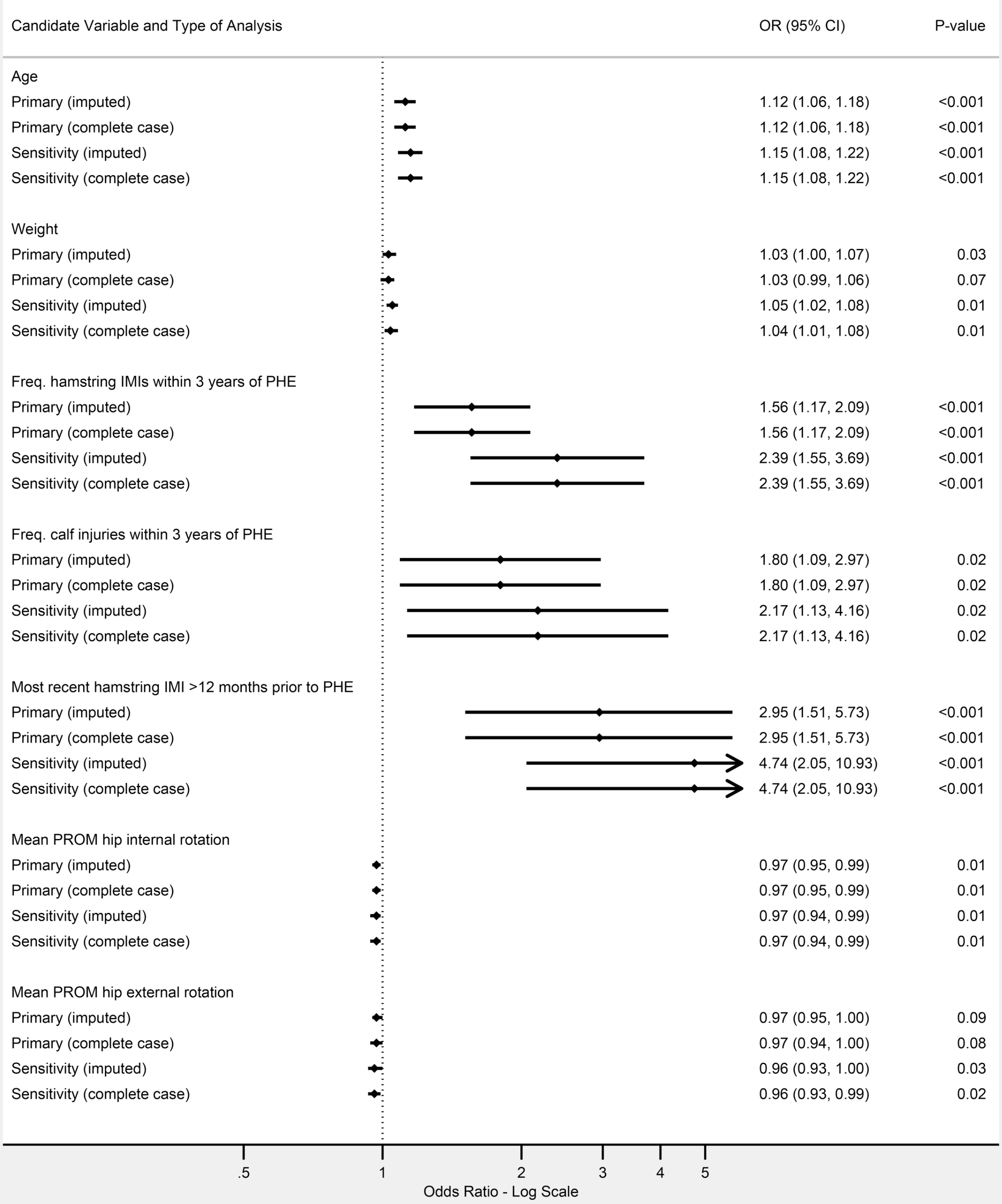

Key: OR = odds ratio; freq. = frequency; IMI = indirect muscle injury; PHE = periodic health examination; PROM = passive range of movement.

For peer review only

Does preseason screening provide a source of potential prognostic factors for indirect muscle injuries in elite football (soccer) players? An exploratory analysis using routinely-collected periodic health examination data

Hughes, T., Riley, R.D., Callaghan, M.J. and Sergeant, J.C. (2021)

Supplementary File 9: Comparison of candidate prognostic factors with statistically significant associations across the primary and sensitivity multivariable analyses (using imputed and complete case datasets).

For peer review only

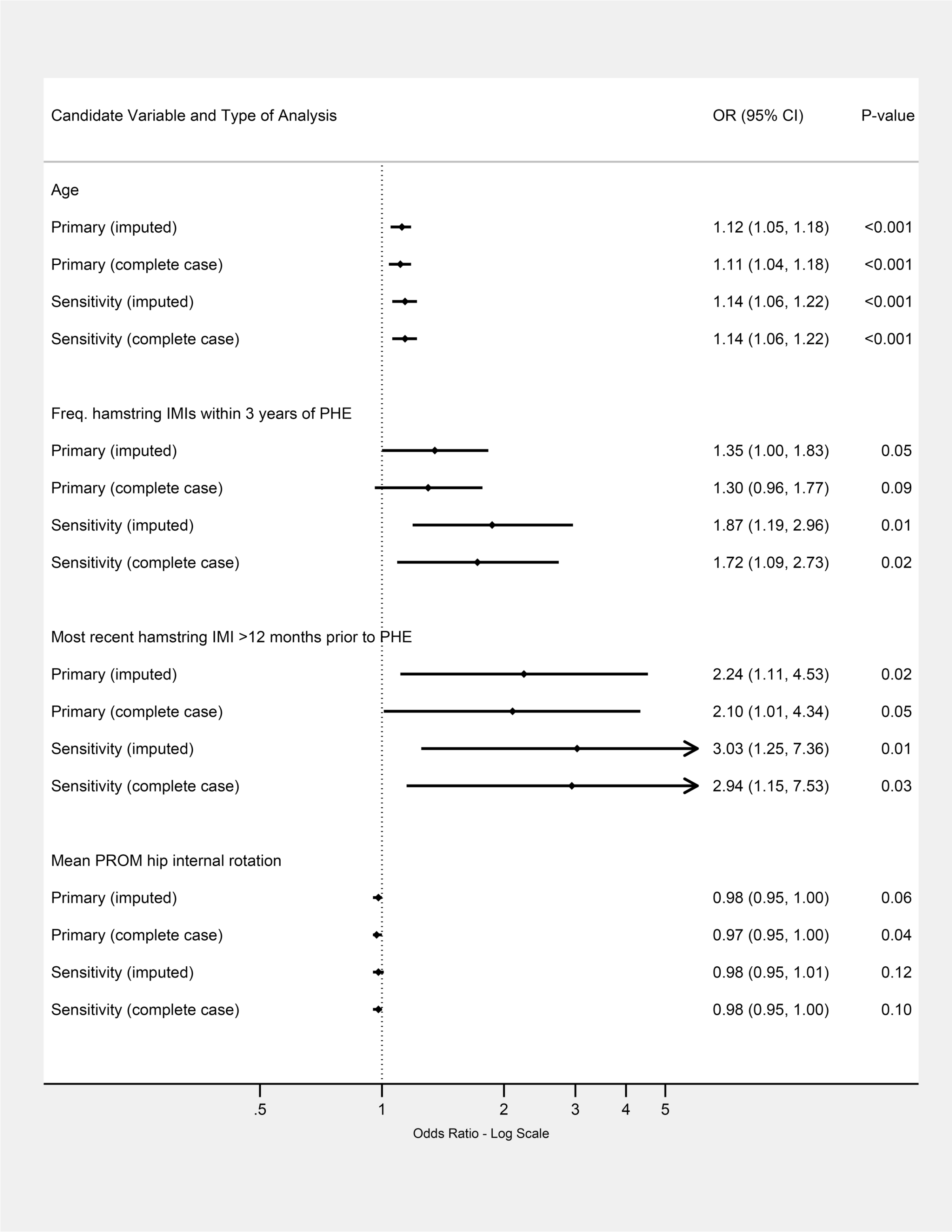

Key: IMI = indirect muscle injury; OR = odds ratio; freq. = frequency; PHE = periodic health examination; PROM = passive range of movement.

Note: the factor of age was adjusted for height and weight. All other factors were adjusted for age, height and weight.

The RECORD statement – checklist of items, extended from the STROBE statement, that should be reported in observational studies using routinely collected health data.

	Item No.	STROBE items	Location in manuscript where items are reported	RECORD items	Location in manuscript where items are reported
**Title and abstract**					
	1	(a) Indicate the study's design with a commonly used term in the title or the abstract (b) Provide in the abstract an informative and balanced summary of what was done and what was found	Page 2	RECORD 1.1: The type of data used should be specified in the title or abstract. When possible, the name of the databases used should be included.<br><br>RECORD 1.2: If applicable, the geographic region and timeframe within which the study took place should be reported in the title or abstract.<br><br>RECORD 1.3: If linkage between databases was conducted for the study, this should be clearly stated in the title or abstract.	Page 2<br><br>Page 2<br><br>n/a
**Introduction**					
Background rationale	2	Explain the scientific background and rationale for the investigation being reported	Pages 4-5		Pages 4-5
Objectives	3	State specific objectives, including any prespecified hypotheses	Page 5		Page 5
**Methods**					

For peer review only

Study Design	4	Present key elements of study design early in the paper	Page 5		Page 5
Setting	5	Describe the setting, locations, and relevant dates, including periods of recruitment, exposure, follow-up, and data collection	Page 6		Page 6

Participants	6	*(a) Cohort study*- Give the eligibility criteria, and the sources and methods of selection of participants. Describe methods of follow-up *Case-control study*- Give the eligibility criteria, and the sources and methods of case ascertainment and control selection. Give the rationale for the choice of cases and controls *Cross-sectional study*- Give the eligibility criteria, and the sources and methods of selection of participants<br><br>*(b) Cohort study*- For matched studies, give matching criteria and number of exposed and unexposed	Page 7<br><br>N/a<br><br>N/a	RECORD 6.1: The methods of study population selection (such as codes or algorithms used to identify subjects) should be listed in detail. If this is not possible, an explanation should be provided.<br><br>RECORD 6.2: Any validation studies of the codes or algorithms used to select the population should be referenced. If validation was conducted for this study and not published elsewhere, detailed methods and results should be provided.<br><br>RECORD 6.3: If the study involved linkage of databases, consider use of a flow diagram or other graphical display to demonstrate the data linkage process, including the number of individuals with linked data at each stage.	Page 7<br><br>N/a<br><br>N/a

For peer review only

		Case-control study- For matched studies, give matching criteria and the number of controls per case	N/a		
Variables	7	Clearly define all outcomes, exposures, predictors, potential confounders, and effect modifiers. Give diagnostic criteria, if applicable.	Pages 6-9	RECORD 7.1: A complete list of codes and algorithms used to classify exposures, outcomes, confounders, and effect modifiers should be provided. If these cannot be reported, an explanation should be provided.	Pages 6-9

For peer review only

Data sources/ measurement	8	For each variable of interest, give sources of data and details of methods of assessment (measurement). Describe comparability of assessment methods if there is more than one group	Page 6		Page 6

Bias	9	Describe any efforts to address potential sources of bias	Pages 6-9		Page 6-9
Study size	10	Explain how the study size was arrived at	Pages 7 -8		Pages 7-8
Quantitative variables	11	Explain how quantitative variables were handled in the analyses. If applicable, describe which groupings were chosen, and why	Pages 10-12		Pages 10-12

For peer review only

Statistical methods	12	(a) Describe all statistical methods, including those used to control for confounding<br>(b) Describe any methods used to examine subgroups and interactions<br>(c) Explain how missing data were addressed<br>(d) *Cohort study*- If applicable, explain how loss to follow-up was addressed<br>*Case-control study*- If applicable, explain how matching of cases and controls was addressed<br>*Cross-sectional study*- If applicable, describe analytical methods taking account of sampling strategy<br>(e) Describe any sensitivity analyses	Pages 10-12<br>Pages 10-12<br>Pages 10-12<br>Pages 10-12<br>Pages 10-12		Pages 10-12<br>Pages 10-12<br>Pages 10-12<br>Pages 10-12<br>Pages 10-12
Data access and cleaning methods		..		RECORD 12.1: Authors should describe the extent to which the investigators had access to the database population used to create the study population.	Pages 5-6

				RECORD 12.2: Authors should provide information on the data cleaning methods used in the study.	Page 6

Linkage		..	N/a	RECORD 12.3: State whether the study included person-level, institutional-level, or other data linkage across two or more databases. The methods of linkage and methods of linkage quality evaluation should be provided.	N/a
**Results**					
Participants	13	(a) Report the numbers of individuals at each stage of the study (*e.g.*, numbers potentially eligible, examined for eligibility, confirmed eligible, included in the study, completing follow-up, and analysed)<br>(b) Give reasons for non-participation at each stage.<br>(c) Consider use of a flow diagram		RECORD 13.1: Describe in detail the selection of the persons included in the study (*i.e.,* study population selection) including filtering based on data quality, data availability and linkage. The selection of included persons can be described in the text and/or by means of the study flow diagram.	Page 6
Descriptive data	14	(a) Give characteristics of study participants (*e.g.*, demographic, clinical, social) and information on exposures and potential confounders<br>(b) Indicate the number of participants with missing data for each variable of interest (c) *Cohort study*- summarise follow-up time (*e.g.*, average and total amount)	Pages 13-14<br><br>Figure 1		Pages 13-14<br><br>Figure 1

For peer review only

Outcome data	15	*Cohort study*- Report numbers of outcome events or summary measures over time<br>*Case-control study*- Report numbers in each exposure	Figure 1 and Pages 12-13		Figure 1 and Pages 12-13
		category, or summary measures of exposure<br>*Cross-sectional study*- Report numbers of outcome events or summary measures			
Main results	16	(a) Give unadjusted estimates and, if applicable, confounder- adjusted estimates and their precision (e.g., 95% confidence interval). Make clear which confounders were adjusted for and why they were included<br>(b) Report category boundaries when continuous variables were categorized<br>(c) If relevant, consider translating estimates of relative risk into absolute risk for a meaningful time period	Page 17		Page 17
Other analyses	17	Report other analyses done—e.g., analyses of subgroups and interactions, and sensitivity analyses	Pages 18 and Supplementary files 5-9		Pages 18 and Supplementary files 5-9

For peer review only

**Discussion**					
Key results	18	Summarise key results with reference to study objectives	Pages 19-20		Pages 19-20
Limitations	19	Discuss limitations of the study, taking into account sources of potential bias or imprecision. Discuss both direction and magnitude of any potential bias	Page 22	RECORD 19.1: Discuss the implications of using data that were not created or collected to answer the specific research question(s). Include discussion of misclassification bias, unmeasured confounding, missing data, and changing eligibility over time, as they pertain to the study being reported.	Page 22
Interpretation	20	Give a cautious overall interpretation of results considering objectives, limitations, multiplicity of analyses, results from similar studies, and other relevant evidence	Page 23-25		Page 23-25
Generalisability	21	Discuss the generalisability (external validity) of the study results	Page 21		Page 21
**Other Information**					
Funding	22	Give the source of funding and the role of the funders for the present study and, if applicable, for the original study on which the present	Page 24		Page 24

For peer review only

		article is based			
Accessibility of protocol, raw data, and programming code		..	Page 24	RECORD 22.1: Authors should provide information on how to access any supplemental information such as the study protocol, raw data, or programming code.	Page 24

*Reference: Benchimol EI, Smeeth L, Guttmann A, Harron K, Moher D, Petersen I, Sørensen HT, von Elm E, Langan SM, the RECORD Working Committee. The REporting of studies Conducted using Observational Routinely-collected health Data (RECORD) Statement. *PLoS Medicine* 2015; in press.

*Checklist is protected under Creative Commons Attribution (CC BY) license.

For peer review only

The REMARK checklist

	Item to be reported	Page no.
**INTRODUCTION**		
1	State the marker examined, the study objectives, and any pre-specified hypotheses.	3-4
**MATERIALS AND METHODS**		
Patients		
2	Describe the characteristics (e.g., disease stage or co-morbidities) of the study patients, including their source and inclusion and exclusion criteria.	5-6
3	Describe treatments received and how chosen (e.g., randomized or rule-based).	n/a
Specimen/Prognostic Factor characteristics		
4	Describe type of biological material/prognostic factors used (including control samples) and methods of preservation and storage.	7-8
Assay methods/Prognostic factor measurement		
5	Specify the assay method used and provide (or reference) a detailed protocol, including specific reagents or kits used, quality control procedures, reproducibility assessments, quantitation methods, and scoring and reporting protocols. Specify whether and how assays were performed blinded to the study endpoint.	8
Study design		
6	State the method of case selection, including whether prospective or retrospective and whether stratification or matching (e.g., by stage of disease or age) was used. Specify the time period from which cases were taken, the end of the follow-up period, and the median follow-up time.	5
7	Precisely define all clinical endpoints examined.	5
8	List all candidate variables initially examined or considered for inclusion in models.	8
9	Give rationale for sample size; if the study was designed to detect a specified effect size, give the target power and effect size.	6
Statistical analysis methods		
10	Specify all statistical methods, including details of any variable selection procedures and other model-building issues, how model assumptions were verified, and how missing data were handled.	9-11
11	Clarify how marker values were handled in the analyses; if relevant, describe methods used for cutpoint determination.	9-11
**RESULTS**		
Data		
12	Describe the flow of patients through the study, including the number of patients included in each stage of the analysis (a diagram may be helpful) and reasons for dropout. Specifically, both overall and for each subgroup extensively examined report the numbers of patients and the number of events.	13
13	Report distributions of basic demographic characteristics (at least age and sex), standard (disease-specific) prognostic variables, and tumor marker, including numbers of missing values.	15
Analysis and presentation		
14	Show the relation of the marker to standard prognostic variables.	18
15	Present univariable analyses showing the relation between the marker and outcome, with the estimated effect (e.g., hazard ratio and survival probability). Preferably provide similar analyses for all other variables being analyzed. For the effect of a tumor marker on a time-to-event outcome, a Kaplan-Meier plot is recommended.	16-20
16	For key multivariable analyses, report estimated effects (e.g., hazard ratio) with confidence intervals for the marker and, at least for the final model, all other variables in the model.	17-21
17	Among reported results, provide estimated effects with confidence intervals from an analysis in which the marker and standard prognostic variables are included, regardless of their statistical significance.	16-21
18	If done, report results of further investigations, such as checking assumptions, sensitivity analyses, and internal validation.	19-22, Supp files 5-7)
**DISCUSSION**		
19	Interpret the results in the context of the pre-specified hypotheses and other relevant studies; include a discussion of limitations of the study.	22-26

Source: McShane LM, Altman DG, Sauerbrei W, Taube SE, Gion M, Clark GM: Reporting recommendations for tumor marker prognostic studies (REMARK). *J Natl Cancer Inst* 2005; 97: 1180-1184.

For peer review only - http://bmjopen.bmj.com/site/about/guidelines.xhtml

The REMARK checklist

20	Discuss implications for future research and clinical value.	

Source: McShane LM, Altman DG, Sauerbrei W, Taube SE, Gion M, Clark GM: Reporting recommendations for tumor marker prognostic studies (REMARK). *J Natl Cancer Inst* 2005; 97: 1180-1184.

For peer review only

BMJ Open

Can prognostic factors for indirect muscle injuries in elite football (soccer) players be identified using data from preseason screening? An exploratory analysis using routinely-collected periodic health examination records.

Journal:	*BMJ Open*
Manuscript ID	bmjopen-2021-052772.R1
Article Type:	Original research
Date Submitted by the Author:	03-Feb-2022
Complete List of Authors:	Hughes, Tom; Manchester United Football Club; University of Manchester<br>Riley, Richard; Keele University<br>Callaghan, Michael J.; Manchester United Football Club; Manchester Metropolitan University<br>Sergeant, Jamie; University of Manchester, Arthritis Research UK Centre for Epidemiology; Manchester Academic Health Science Centre, Centre for Biostatistics
**Primary Subject Heading**:	Sports and exercise medicine
Secondary Subject Heading:	Sports and exercise medicine
Keywords:	SPORTS MEDICINE, REHABILITATION MEDICINE, Musculoskeletal disorders < ORTHOPAEDIC & TRAUMA SURGERY

SCHOLARONE™
Manuscripts

I, the Submitting Author has the right to grant and does grant on behalf of all authors of the Work (as defined in the below author licence), an exclusive licence and/or a non-exclusive licence for contributions from authors who are: i) UK Crown employees; ii) where BMJ has agreed a CC-BY licence shall apply, and/or iii) in accordance with the terms applicable for US Federal Government officers or employees acting as part of their official duties; on a worldwide, perpetual, irrevocable, royalty-free basis to BMJ Publishing Group Ltd ("BMJ") its licensees and where the relevant Journal is co-owned by BMJ to the co-owners of the Journal, to publish the Work in this journal and any other BMJ products and to exploit all rights, as set out in our licence.

The Submitting Author accepts and understands that any supply made under these terms is made by BMJ to the Submitting Author unless you are acting as an employee on behalf of your employer or a postgraduate student of an affiliated institution which is paying any applicable article publishing charge ("APC") for Open Access articles. Where the Submitting Author wishes to make the Work available on an Open Access basis (and intends to pay the relevant APC), the terms of reuse of such Open Access shall be governed by a Creative Commons licence – details of these licences and which Creative Commons licence will apply to this Work are set out in our licence referred to above.

Other than as permitted in any relevant BMJ Author's Self Archiving Policies, I confirm this Work has not been accepted for publication elsewhere, is not being considered for publication elsewhere and does not duplicate material already published. I confirm all authors consent to publication of this Work and authorise the granting of this licence.

For peer review only

Can prognostic factors for indirect muscle injuries in elite football (soccer) players be identified using data from preseason screening? An exploratory analysis using routinely-collected periodic health examination records.

Tom Hughes, PhD,[1,2,3]
Professor Richard D. Riley, PhD,[4]
Professor Michael J. Callaghan, PhD, *[1,2,3]
Dr. Jamie C. Sergeant, PhD. * [2 5]

[1] Manchester United Football Club, AON Training Complex, Birch Road, Off Isherwood Road, Carrington, Manchester. UK.

[2] Centre for Epidemiology Versus Arthritis, Centre for Musculoskeletal Research, Manchester Academic Health Science Centre, University of Manchester, Manchester, UK.

[3] Department of Health Professions, Manchester Metropolitan University, Brooks Building, Bonsall Street, Manchester. UK.

[4] Centre for Prognosis Research, School of Medicine, Keele University, Staffordshire, UK.

[5] Centre for Biostatistics, University of Manchester, Manchester Academic Health Science Centre, Manchester, UK.

***JCS and MJC contributed equally and are joint last authors**

Corresponding author: Tom Hughes

Email: tom.hughes.physio@manutd.co.uk
Twitter: @dt_hughes
ORCID ID: orcid.org/0000-0003-2266-6615

Correspondence address: Manchester United Football Club, AON Training Complex, Birch Road, Off Isherwood Road, Carrington, Manchester. UK. M31 4BH.
Tel: 0161 868 8754

WORD COUNT: 3989 (excluding title, abstract, tables, figures and references)

For peer review only

ABSTRACT

Background: In elite football, periodic health examination (PHE) may be useful for injury risk prediction.

Objective: To explore whether PHE-derived variables are prognostic factors for indirect muscle injuries (IMIs) in elite players.

Design: Retrospective cohort study

Setting: An English Premier League football club.

Participants: 134 outfield elite male players, over 5 seasons (1st July 2013 to 19th May 2018).

Outcome and analysis: The outcome was any time-loss, lower extremity index indirect muscle injury (I-IMI). Prognostic associations were estimated using odds ratios (ORs) and corresponding statistical significance for 36 variables, derived from univariable and multivariable logistic regression models. Missing data were handled using multiple imputation. Non-linear associations were explored using fractional polynomials.

Results: During 317 participant-seasons, 138 I-IMIs were recorded. Univariable associations were determined for previous calf IMI frequency (OR=1.80, 95% confidence interval (CI) = 1.09 to 2.97), hamstring IMI frequency (OR=1.56, 95% CI=1.17 to 2.09), if the most recent hamstring IMI occurred > 12 months but < 3 years prior to PHE (OR= 2.95, 95% CI = 1.51 to 5.73) and age (OR =1.12 per 1-year increase, 95% CI = 1.06 to 1.18). Multivariable analyses showed that if a player's most recent previous hamstring IMI was >12 months but <3 years prior to PHE (OR= 2.24, 95% CI = 1.11 to 4.53), this was the only variable with added prognostic value over and above age, which was a confirmed prognostic factor (OR=1.12 per 1-year increase, 95%CI = 1.05 to 1.18). Allowing non-linear associations conferred no advantage over linear associations.

Conclusion: PHE has limited use for injury risk prediction. Most variables did not add prognostic value over and above age, other than if a player experienced a hamstring IMI >12 months but < 3 years prior to PHE. However, the precision of this prognostic association should be confirmed in future.

Trial registration number = NCT03782389

WORD COUNT = 300

For peer review only

For peer review only - http://bmjopen.bmj.com/site/about/guidelines.xhtml

STRENGTHS AND LIMITATIONS OF THIS STUDY

- This study investigated a wide selection of PHE-derived variables and their association with index indirect muscle injuries in a cohort of elite football players, over and above standard anthropometric variables of age, height and weight.
- This is the first known study in elite football to explore any non-linear associations between injury outcomes and variables measured on a continuous scale, using a fractional polynomial approach.
- High-quality cohort data were used, with variables reliably measured in pre-season and any subsequent injury outcomes confirmed using a validated muscle injury classification system.
- Some participants had missing data for some factors; a multiple imputation approach was used to help address this, under a missing at random assumption.
- Candidate factors were only measured at one timepoint each season, which means that dynamic associations were not investigated.

For peer review only

For peer review only - http://bmjopen.bmj.com/site/about/guidelines.xhtml

BACKGROUND

Periodic health examination (PHE), or screening, is a well-established clinical evaluation strategy in elite football.[1] Typically during PHE, players undertake various medical, musculoskeletal, functional and performance tests[2] during preseason and in-season periods.[1] PHE allows opportunities for general health surveillance, identification of salient pathology[3] and monitoring of rehabilitation or performance.[4] In addition, although PHE cannot establish specific causal factors for injuries,[4] it is perceived to be useful for the prediction of future injury risk in athletes,[2, 4] which could prove especially valuable for injury types that are problematic in terms of incidence and severity. Indirect (non-contact) muscle injuries (IMIs) are an obvious example, because they account for between 30.3% to 47.9% of all injuries observed in elite football[5-9] and each IMI typically results in 14.4 [5]to 15 days lost to training and competition.[8]

To be able to predict the risk of future health events, prognostic factors are required.[10] In the context of football, prognostic factors could be any PHE derived variables, characteristics or measurements (e.g. medical history, leg strength or range of motion tests) that are associated with increased injury risk through causal or non-causal pathways.[4] There is clinical value in gaining a deeper understanding of prognostic factors associated with injuries such as IMIs. Specifically, prognostic factors can help practitioners understand the differences in risk (outcome event probability) between players, and therefore explain why some players may have a better or worse prognosis than others.[4] Furthermore, prognostic factors that have an established causal role in injury occurrence can inform the selection of injury mitigation strategies, relevant for subgroups of players who share such characteristics.[4] Finally, causal prognostic factors can also be used to develop innovative intervention approaches aimed at mitigating risk.[10]

For peer review only - http://bmjopen.bmj.com/site/about/guidelines.xhtml

Despite these benefits, the predictive power of single prognostic factors are limited.[11, 12] However, if several prognostic factors are used in combination within a multivariable prognostic model, it may be possible to produce useful individualised risk estimates[10, 11] that can be used to communicate risks to practitioners and coaches.[13] Additionally, if developed using prognostic factors which have a causal role in injury risk, prognostic models could also be used to assist practitioners in selecting an array of specific risk reduction interventions that are bespoke to the prognostic factor profile of individual players[4].

Because the predictive function of PHE remains unsubstantiated[3, 14] and given that indirect (non-contact) muscle injuries (IMIs) are the most significant problem observed in elite football,[5-9] a related multivariable prognostic model was recently developed to predict individualised lower extremity IMI risk in elite players using PHE data.[13] However, sample size limitations meant that only 10 candidate prognostic factors could be considered in the model and these were selected using data quality assessment, clinical reasoning, or on the basis of a related systematic review.[14] The performance of the model was modest and it was concluded that implementing it in practice would not be beneficial.[13]

Furthermore, several methodological limitations of the current evidence have been previously highlighted, which specifically included inadequate reporting of outcomes, prognostic factor measurement and reliability.[14] Additionally, while most studies performed appropriate statistical analyses, continuous prognostic factor measurements were often categorised[15-18] and non-linear associations were not investigated,[15-21] which does not conform to current methodological recommendations.[22-24]

For peer review only - http://bmjopen.bmj.com/site/about/guidelines.xhtml

To further the development of IMI prognostic models and improve understanding of how differences in IMI risk may occur between individuals, there is a clear need to ascertain the existence of other robust and novel prognostic factors.[13] Therefore, this study used routinely collected data from a 5-season period to explore; 1) prognostic associations between PHE-derived data and IMI outcomes in elite footballers, using a broader dataset than had been considered in the development the previous prognostic model[13] and; 2) the prognostic value of these PHE-derived data over and above standard anthropometric data, including age (which has previously confirmed prognostic value[13, 14]), height and weight. Both linear and non-linear associations were also explored which, as far as is known, has not been conducted previously.

METHODS

The methodology has been described in a published protocol[25] so will only be briefly outlined. This study was registered on ClinicalTrials.gov (NCT03782389) and was reported according to the Reporting Recommendations for Marker Prognostic Studies (REMARK).[26] Given the number of PHE-related variables examined, our study should be viewed as exploratory, but we emphasise that this an important phase in prognostic factor research.[12, 27]

Data Sources

This study was of retrospective cohort design. Eligible participants were identified from a population of male elite footballers, aged 16-40 years old at an English Premier League football club. A database was created by the principal investigator (TH) using routinely collected injury records and preseason PHE data over 5 seasons (1st July 2013 to 19th May 2018). This process included checks for accuracy, duplicate or missing entries. Participants

For peer review only - http://bmjopen.bmj.com/site/about/guidelines.xhtml

completed a mandatory PHE during the first week of each season (which started on 1st July), and were followed up to the last first team game of the season.

The PHE process typically included: 1) anthropometric measurements; 2) a review of medical and previous injury history; 3) musculoskeletal examination tests; 4) functional movement and balance tests; 5) strength and power tests. A description of all of the included test procedures is presented in Supplementary File 1. The PHE test order was self-selected by each player and a standardised warm up was not implemented, although players could undertake their own warm up procedures if they wished. Each component of PHE was standardised according to a written protocol and was examined by physiotherapists, sports scientists or club medical doctors. The same examiners performed the same test every season to eliminate inter-tester variability. No examiner attrition occurred throughout the data collection period. If a participant was injured at the scheduled time of PHE, a risk assessment was completed by medical staff and participants only completed tests that were deemed appropriate and safe for the participant's condition; examiners were therefore not blinded to injury status.

Eligibility criteria

During any season, participants were eligible if they: 1) were not a goalkeeper; 2) participated in PHE for the relevant season. Participants were excluded if they were not under contract to the Club at the time of PHE.

For peer review only

Ethics and Data Use

Informed consent was not required as data were captured from the mandatory PHE completed through the participants' employment. The data usage was approved by the Club and the Research Ethics Service at the University of Manchester.

Patient and public involvement

Participants and members of the public were not involved in the study design.

For peer review only

Outcome

The outcome was any time-loss, index lower extremity IMI (I-IMI) sustained by a participant during match play or training, which affected any lower abdominal, hip, thigh, calf or foot muscle groups and prohibited future match or training participation.[28] I-IMIs were confirmed and graded by a club doctor or physiotherapist according to the previously validated Munich Consensus Statement for the Classification of Muscle Injuries in Sport,[29, 30] during routine assessments undertaken within 24h of injury occurrence. The medical professionals were not blinded to PHE data at diagnosis.

Sample size

Our sample size of 317 participant-seasons (with 138 I-IMI events) had 80% power to detect an adjusted odds ratio (OR) of at least 1.6 for a 1 standard deviation increase in a variable of interest, conservatively assuming a correlation of 0.5 with the adjustment variables of age, height and weight (see Supplementary File 2 for the sample size calculation).[31]

For peer review only - http://bmjopen.bmj.com/site/about/guidelines.xhtml

PHE-derived Candidate Variables

To aid clarity in this study, the term ‘prognostic factor’ is reserved for factors found to have a prognostic association with an I-IMI outcome (i.e. with statistical evidence established during the analyses), whereas the term ‘candidate variables’ relates to all factors for which the association with I-IMI outcome was investigated during the analyses.

As described in the study protocol, the dataset contained 60 variables[25] that were eligible for analysis unless there were >15% missing observations or if reliability (where applicable) was reported as fair to poor (that is, Intraclass Correlation Coefficient (ICC) < 0.70).[25, 32] If any variables did not meet these eligibility criteria, they were excluded (Supplementary File 3). Collinearity between eligible variables was assessed with a scatterplot matrix; this was evident when tests were used to measure right and left limbs independently.[25] In these circumstances, composite variables were created for between-limb differences and the mean of the test measurements for both limbs, as described in the study protocol.[25]

Of the remaining eligible variables, 10 were used in a previous study to develop a multivariable prognostic model for I-IMI prediction (represented by 12 parameters).[13] With the exception of age at PHE (which was used for adjustment purposes in this study), these candidates were therefore excluded.[25] The final number of candidate variables included for exploratory analysis was 36. Table 1 summarises all included variables with their measurement units and data type, as well as the measurement methods, their reliability and validity.

For peer review only - http://bmjopen.bmj.com/site/about/guidelines.xhtml

Table 1: List of candidate variables with a summary of the units of measurement, methods and reliability of measurement and data type.

Candidate Variable Type	*Name of candidate variable*	*Candidate variable Identification number*	*Measurement unit*	*Measurement Method*	*Reliability (if applicable/available)*	*Validity (if applicable/available)*	*Data type*
**Anthropometric**	Age	1	Years	Medical records	-	-	Cont.
	Height	2	Centimetres (cm)	Standing height measure	-	-	Cont.
	Weight	3	kilograms (kg)	Digital scales	-	-	Cont.
Medical history	*Within 3 years prior to PHE, the frequency of:* Foot/ankle injuries	4	Count	Medical records	-	-	Dis./cont.
	Hip/groin injuries	5	Count	Medical records	-	-	Dis./cont.
	Knee injuries	6	Count	Medical records	-	-	Dis./cont.
	Shoulder injuries	7	Count	Medical records	-	-	Dis./cont.
	Lumbar spine injuries	8	Count	Medical records	-	-	Dis./cont.
	Iliopsoas injuries	9	Count	Medical records	-	-	Dis./cont.
	Hip adductor IMIs	10	Count	Medical records	-	-	Dis./cont.
	Hamstring IMIs	11	Count	Medical records	-	-	Dis./cont.
	Quadriceps IMIs	12	Count	Medical records	-	-	Dis./cont.
	Calf IMIs	13	Count	Medical records	-	-	Dis./cont.
	Within 3 years prior to PHE, the most recent Foot/ankle injury	14	Never, < 6 months, 6-12 months, > 12 months.	Medical records	-	-	Cat.
	Hip/groin injury	15	Never, < 6 months, 6-12 months, > 12 months.	Medical records	-	-	Cat.
	Knee injury	16	Never, < 6 months, 6-12 months, > 12 months.	Medical records	-	-	Cat.
	Shoulder injury	17	Never, < 6 months, 6-12 months, > 12 months.	Medical records	-	-	Cat.
	Lumbar spine injury	18	Never, < 6 months, 6-12 months, > 12 months.	Medical records	-	-	Cat.
	Iliopsoas injury	19	Never, < 6 months, 6-12 months, > 12 months.	Medical records	-	-	Cat.
	Hip adductor IMI	20	Never, < 6 months, 6-12 months, > 12 months.	Medical records	-	-	Cat.
	Hamstring IMI	21	Never, < 6 months, 6-12 months, > 12 months.	Medical records	-	-	Cat.
	Quadriceps IMI	22	Never, < 6 months, 6-12 months, > 12 months.	Medical records	-	-	Cat.
	Calf IMI	23	Never, < 6 months, 6-12 months, > 12 months.	Medical records	-	-	Cat.
Musculoskeletal	Mean PROM hip internal rotation (IR)	24	Degrees	Digital inclinometer + ROM	Intra-rater ICC= 0.90[33]	-	Cont.
	Mean PROM hip external rotation (ER)	25	Degrees	Digital inclinometer + ROM	Intra-rater ICC= 0.90[33]	-	Cont.
	Mean hip flexor length	26	Degrees	Digital inclinometer + Thomas Test	Inter-rater ICC = 0.89[34]	Concurrent validity with handheld goniometer (r=0.86-0.92)[34] and IMC (r=0.49-0.53)[35]	Cont.
	Mean hamstring length/neural mobility	27	Degrees	Digital inclinometer + SLR	Intra-rater ICC = 0.95-0.98[36] Inter-rater ICC = 0.80-0.97[36]	Construct validity with handheld inclinometer (r=0.98-0.99)[37]	Cont.
	Mean calf muscle length	28	Degrees	Digital inclinometer + WBL	Inter-rater ICC = 0.80- 0.95[38, 39] Intra-rater ICC = 0.88[39]	Concurrent validity of inclinometer with 2D motion analysis (r range=0.71-0.76)[40]	Cont.
Strength/power	Max. leg extension power difference	29	Normalised watts per kilo ($W/kg^{-0.67}$)	Double leg press using Keiser Air 300 machine	Test-retest ICC = 0.886[41]	Concurrent validity with mounted force plate (r=0.952)[42]	Cont.
	Mean of max. leg extension power	30	Normalised watts per kilo ($W/kg^{-0.67}$)	Double leg press using Keiser Air 300 machine	Test-retest ICC = 0.886[41]	Concurrent validity with mounted force plate (r=0.977)[42]	Cont.
	Max. leg extension velocity difference	31	Peak velocity ($m.s^{-1}$)	Double leg press using Keiser Air 300 machine	Test-retest ICC = 0.792[41]	Concurrent validity with mounted force plate (r=0.999)[42]	Cont.
	Mean of max. leg extension velocity	32	Peak velocity ($m.s^{-1}$)	Double leg press using Keiser Air 300 machine	Test-retest ICC = 0.792[41]	Concurrent validity with mounted force plate (r=0.999) [42]	Cont.
	Max leg extension force difference	33	Normalised peak force ($N/Kg^{-0.67}$)	Double leg press using Keiser Air 300 machine	Test-retest ICC = 0.914[41]	Concurrent validity with mounted force plate (r=0.994)[42]	Cont.
	Mean of max. leg extension force	34	Normalised peak force ($N/Kg^{-0.67}$)	Double leg press using Keiser Air 300 machine	Test-retest ICC = 0.914[41]	Concurrent validity with mounted force plate (r=0.994)[42]	Cont.
	CMJ Force per kg of body mass	35	Force per kg (N/kg)	Countermovement jump (CMJ) + force plate	Test-retest ICC = 0.80-0.88[43]	Concurrent validity with other force plate (r≥0.99)[44]	Cont.
	CMJ height	36	Centimetres (cm)	Countermovement jump (CMJ) + force plate	Test-retest ICC = 0.80-0.88[43]	Concurrent validity with other force plate (r≥0.99)[44]	Cont.

For peer review only

For peer review only - http://bmjopen.bmj.com/site/about/guidelines.xhtml

Key: BMI= body mass index; cat.= categorical; Cont.=continuous; cm = centimetres; CMJ=countermovement jump; deg. = degrees; dis./cont.= discrete treated as continuous; grp=group; ICC=intraclass correlation coefficient; I-IMI= index indirect muscle injury; IMC= inertial motion capture; Kg=kilograms; kg/m^2 = kilograms/body height squared; max.=maximum; m.s.= metres per second; N= newtons (note: N/kg$^{-0.67}$ has a scaling factor to normalise force to body mass [45]); PROM=passive range of movement; SLR= straight leg raise; W= watts (note: W/kg$^{-0.67}$ has a scaling factor to normalise force to body mass [45]); WBL=weight bearing lunge.

For peer review only

Statistical analysis

Data handling – outcome measures

Each participant-season was treated as independent. If an I-IMI occurred, the participant's outcome was determined for that season and they were no longer considered at risk. In these circumstances, participants were included for further analysis at the start of the consecutive season, if still eligible. Any upper limb IMI, trunk IMI or non-IMI injuries were ignored and participants were still considered at risk.

Eligible participants who were loaned to another club throughout that season, but had not sustained an I-IMI prior to the loan were still considered at risk. I-IMIs that occurred whilst on loan were included for analysis. Permanently transferred participants (who had not sustained an I-IMI prior to the transfer), were recorded as not having an I-IMI during the relevant season and exited the cohort at the season end.

Data Handling – Missing data

Missing values were assumed to be missing at random (i.e. missingness could be predicted conditional on other known variables).[25] The continuous parameters generally demonstrated non-normal distributions, so were transformed using normal scores[46] to approximate normality before imputation, and back-transformed following imputation.[47] Multivariate normal multiple imputation was performed, using a model that included all candidate variables and I-IMI outcomes. Fifty imputed datasets were created in Stata 15.1 (StataCorp LLC, Texas, USA) using the '*mi impute*' command.

For peer review only - http://bmjopen.bmj.com/site/about/guidelines.xhtml

Univariable and multivariable analyses

All data were analysed in the form that they were recorded. In particular, variables that were recorded as continuous were kept continuous and not categorised, to avoid a loss of prognostic information.[22] Univariable logistic regression models were used to estimate the unadjusted linear associations between I-IMIs and each candidate variable. Multivariable logistic regression models were also used to estimate the linear association between I-IMIs and each variable, after adjustment for age (which has confirmed prognostic importance[13, 14]), height and weight (which were both considered as potential confounders for I-IMIs and PHE-derived candidates). All parameter estimates were averaged across all imputed datasets using Rubin's Rules[48] and were computed using the '*mim*' module in Stata 15.1. Statistical significance thresholds were used to indicate the strength of exploratory evidence against null associations, where p-values of : 1) <0.05 indicated strong evidence and the factor was considered significant; 2) 0.05 to 0.10 indicated weak evidence and; 3) >0.10 indicated little or no evidence.[49] Prognostic importance was also considered by checking the magnitude of prognostic effects encompassed by the width of 95% confidence intervals.

For all variables, non-linear associations with the outcome were also explored using fractional polynomials for the univariable and multivariable models; the fit of first and second order fractional polynomial models were evaluated against the fit of the standard logistic regression models.[50] The parameter estimates were combined across all imputed datasets[51] using Rubins Rules,[48] with the automated '*mfpmi*' algorithm in Stata 15.1, using a significance threshold set at p< 0.05. All analyses are summarised in Table 2.

For peer review only - http://bmjopen.bmj.com/site/about/guidelines.xhtml

Table 2: Summary of all statistical analyses performed

Statistical analyses of I-IMI outcomes					
**Analysis performed**	**Participant-seasons**	**Number of I-IMIs**	**Variables considered**	**Adjusted for:**	**Results**
Primary analysis, imputed data					
A1: Univariable Logistic Regression/FPs	317	138	Individual models for I-IMI + each variable (1-36)	None	Table 4
A2: Multivariable Logistic Regression/FPs	317	138	Individual models for I-IMI + each variable (4-36)	Variables 1-3 (age, height, weight)	Table 4
Primary analysis, complete case data					
B1: Univariable Logistic Regression/FPs	265	115	Individual models for I-IMI + each variable (1-36)	None	SF 6
B2: Multivariable Logistic Regression/FPs	265	115	Individual models for I-IMI + each variable (4-36)	Variables 1-3 (age, height, weight)	SF 6
Sensitivity analysis, imputed data					
C1: Univariable Logistic Regression/FPs	260	129	Individual models for I-IMI + each variable (1-36)	None	SF 7
C2: Multivariable Logistic Regression/FPs	260	129	Individual models for I-IMI + each variable (4-36)	Variables 1-3 (age, height, weight)	SF 7
Sensitivity analysis, complete case data					
D1: Univariable Logistic Regression/FPs	217	106	Individual models for I-IMI + each variable (1-36)	None	SF 8
D2: Multivariable Logistic Regression/FPs	217	106	Individual models for I-IMI + each variable (4-36)	Variables 1-3 (age, height, weight)	SF 8

Key: FP= fractional polynomials; I-IMI= index indirect muscle injury; SF= supplementary file

Primary and sensitivity analyses

To determine the effect of imputation and player transfers on variable associations, the analyses were repeated: 1) as complete cases analyses; and 2) as sensitivity analyses excluding participant-seasons for participants who were loaned or transferred (performed as both multiple imputation and complete case analyses). All primary complete case and sensitivity analyses are also summarised in Table 2.

RESULTS

Participants

During the five seasons, 134 participants were included, contributing 317 participant-seasons and 138 IMIs in the primary analysis (Figure 1). Three players were classified as injured at the time of PHE (which affected three participant-seasons). This meant they were unavailable for selection for training or matches at that time. However, these players had commenced football specific, field-based rehabilitation so also had similar exposure to training activities

For peer review only

to uninjured players and were therefore included in the cohort because it was reasonable to assume that they could also be considered at risk of an I-IMI event.

For the sensitivity analyses (excluding loans and transfers), 260 independent participant-seasons with 129 IMIs were included; 36 participants were transferred on loan, while 14 participants were permanently transferred during a season, which excluded 57 participant-seasons (Figure 1).

INSERT FIGURE 1 HERE

Table 3 summarises the participant characteristics and candidate variable values for participants included in the primary analyses. All values were similar to those included in the sensitivity analyses (Supplementary File 4).

Missing data and multiple imputation

Data were complete for age and all past medical history variables (Table 3). For all other candidates, the proportion of missing data ranged from 5.68% (for height and weight) to 14.20% (for the mean and between limb differences of maximal leg extension power and force) (Table 3). For all continuous variables, the distribution of imputed values approximated the observed values (Supplementary File 5), therefore confirming their plausibility.

For peer review only

Table 3. Characteristics of included participants

Characteristics/candidate variables		*Min*	*Lower quartile*	*Median*	*Upper quartile*	*Max.*	*Freq. (%) - if categorical*	*Missing values n (%)*
**Anthropometrics**								
1. Age at PHE (years)		16.01	17.80	19.69	23.56	39.59	-	0 (0)
2. Height (cm)		164.3	176.0	180.0	185.5	195.0	-	18 (5.68)
3. Weight (kg)		56.8	69.2	73.6	80.0	94.0	-	18 (5.68)
**Past medical history**								
Within 3 years prior to PHE, freq. of:								
4. foot/ankle injuries		0	0	1	2	7	-	0 (0)
5. Hip/groin injuries		0	0	0	1	5	-	0 (0)
6. Knee injuries		0	0	0	1	3	-	0 (0)
7. Shoulder injuries		0	0	0	0	2	-	0 (0)
8. lumbar spine injuries		0	0	0	0	3	-	0 (0)
9. iliopsoas IMIs		0	0	0	0	2	-	0 (0)
10. hip adductor IMIs		0	0	0	0	3	-	0 (0)
11.hamstring IMIs		0	0	0	1	6	-	0 (0)
12. quadriceps IMIs		0	0	0	0	3	-	0 (0)
13. calf IMIs		0	0	0	0	4	-	0 (0)
Within 3 years prior to PHE, most recent:								
14. foot/ankle injury	*Never*	-	-	-	-	-	143 (45.11)	0 (0)
	< 6 months	-	-	-	-	-	43 (13.56)	0 (0)
	6-12 months	-	-	-	-	-	34 (10.73)	0 (0)
	>12 months	-	-	-	-	-	97 (30.60)	0 (0)
15. Hip/groin injury	*Never*	-	-	-	-	-	217 (68.45)	0 (0)
	< 6 months	-	-	-	-	-	23 (7.26)	0 (0)
	6-12 months	-	-	-	-	-	23 (7.26)	0 (0)
	>12 months	-	-	-	-	-	54 (17.03)	0 (0)
16. Knee injury	*Never*	-	-	-	-	-	201 (63.41)	0 (0)
	< 6 months	-	-	-	-	-	15 (4.73)	0 (0)
	6-12 months	-	-	-	-	-	31 (9.78)	0 (0)
	>12 months	-	-	-	-	-	70 (22.08)	0 (0)
17. Shoulder injury	*Never*	-	-	-	-	-	297 (93.69)	0 (0)
	< 6 months	-	-	-	-	-	6 (1.89)	0 (0)
	6-12 months	-	-	-	-	-	4 (1.26)	0 (0)
	>12 months	-	-	-	-	-	10 (3.15)	0 (0)
18. lumbar spine injury	*Never*	-	-	-	-	-	264 (83.28)	0 (0)
	< 6 months	-	-	-	-	-	8 (2.52)	0 (0)
	6-12 months	-	-	-	-	-	9 (2.84)	0 (0)
	>12 months	-	-	-	-	-	36 (11.36)	0 (0)
19. iliopsoas IMI	*Never*	-	-	-	-	-	287 (90.54)	0 (0)
	< 6 months	-	-	-	-	-	2 (0.63)	0 (0)
	6-12 months	-	-	-	-	-	9 (2.84)	0 (0)
	>12 months	-	-	-	-	-	19 (5.99)	0 (0)
20. hip adductor IMI	*Never*	-	-	-	-	-	263 (82.92)	0 (0)
	< 6 months	-	-	-	-	-	18 (5.68)	0 (0)
	6-12 months	-	-	-	-	-	12 (3.79)	0 (0)
	>12 months	-	-	-	-	-	24 (7.57)	0 (0)
21.hamstring IMI	*Never*	-	-	-	-	-	231 (72.87)	0 (0)
	< 6 months	-	-	-	-	-	11 (3.47)	0 (0)
	6-12 months	-	-	-	-	-	30(9.46)	0 (0)
	>12 months	-	-	-	-	-	45 (14.20)	0 (0)
22. quadriceps IMI	*Never*	-	-	-	-	-	267 (84.23)	0 (0)
	< 6 months	-	-	-	-	-	7 (2.21)	0 (0)
	6-12 months	-	-	-	-	-	13 (4.10)	0 (0)
	>12 months	-	-	-	-	-	30 (9.46)	0 (0)
23. calf IMI	*Never*	-	-	-	-	-	283 (89.27)	0 (0)
	< 6 months	-	-	-	-	-	11 (3.47)	0 (0)
	6-12 months	-	-	-	-	-	6 (1.89)	0 (0)
	>12 months	-	-	-	-	-	17 (5.36)	0 (0)
**Musculoskeletal Examination**								
24. Mean PROM hip IR (deg.)		9.5	22.5	33.0	40.0	55.0	-	20 (6.31)
25. Mean PROM hip ER (deg.)		17.5	33.5	38.5	43.0	62.0	-	20 (6.31)
26. Mean hip flexor length (deg.)		-7.0	3.5	9.0	15.0	55.0	-	23 (7.26)
27. Mean hamstring/neural mobility length (deg.)		45.0	84.0	90.0	90.0	102.0	-	20 (6.31)
28. Mean calf muscle length (deg.)		9.5	25.0	30.0	36.0	57.5	-	20 (6.31)
**Strength/Power Tests**								
29: Max. leg extension power difference ($W/kg^{-0.67}$)		-11.94	-1.55	0.29	1.77	15.26	-	45 (14.20)
30: Mean of max. leg extension power ($W/kg^{-0.67}$)		23.01	42.12	46.52	51.95	78.69	-	45 (14.20)

For peer review only - http://bmjopen.bmj.com/site/about/guidelines.xhtml

31: Max. leg extension velocity difference (m.s -1)	-0.34	-0.05	0.02	0.11	0.40	-	41 (12.93)
32: Mean of max. leg extension velocity (m.s -1)	1.02	1.68	1.84	1.98	2.35	-	41 (12.93)
33: Max leg extension force difference (N/kg-0.67)	-83.59	-6.18	1.17	4.40	55.41	-	45 (14.20)
34: Mean of max. leg extension force (N/kg-0.67)	50.19	98.59	101.44	113.44	217.95	-	45 (14.20)
35: CMJ Force per kg of body mass	20.20	23.40	25.40	28.04	39.20	-	42 (13.25)
36: CMJ height (cm)	28.7	37.3	40.2	43.0	58.0	-	42 (13.25)

Key: BMI= body mass index; cat.= categorical; Cont.=continuous; cm = centimetres; CMJ=countermovement jump; deg. = degrees; dis./cont.= discrete treated as continuous; Freq= frequency; grp=group; ICC=intraclass correlation coefficient; IMI= indirect muscle injury; I-IMI= index indirect muscle injury; IQR= interquartile range; Kg=kilograms; kg/m² = kilograms/body height squared; max.=maximum; m.s.= metres per second; N= newtons (note: N/kg^-0.67 has a scaling factor to normalise force to body mass [45]); PHE= periodic health examination; PROM=passive range of movement; SLR= straight leg raise; W= watts (note: W/kg^-0.67 has a scaling factor to normalise force to body mass [45]); WBL=weight bearing lunge; - = not applicable. Note that for the Musculoskeletal Examination and Strength/Power factors, positive values indicate greater left limb values compared to right limb values; negative values indicate greater right limb values compared to left limb values.

Univariable analyses

Table 4 shows the results of the univariable analyses. The continuous variables of age (OR= 1.12 for a 1-year increase, 95% confidence interval (CI)=1.06 to 1.18, p<0.001), weight (OR=1.03 for a 1kg increase, 95% CI=1.00 to 1.07, p=0.03) and mean hip IR PROM (OR= 0.97 for a 1-degree increase, 95% CI = 0.95 to 0.99, p=0.01) showed a significant but modest association with I-IMIs. The narrow CIs indicated that these estimates were relatively precise. Linear associations were the best fit for all these continuous variables. Significant associations with larger OR estimates were observed for previous calf IMI frequency (OR=1.80, 95% CI = 1.09 to 2.97, p=0.02), hamstring IMI frequency (OR=1.56, 95% CI=1.17 to 2.09, p<0.001), and if the most recent hamstring IMI occurred more than 12 months but less than 3 years prior to PHE (OR= 2.95, 95% CI = 1.51 to 5.73, p<0.001). The wider CIs for these estimates indicated greater imprecision about the prognostic effect; this may because these candidates were either discrete or categorical, rather than continuous.

Despite relatively large ORs, weaker evidence of associations was observed for the frequency of previous shoulder injuries (OR=2.38, 95% CI = 0.98 to 5.75, p=0.05) and if the most recent calf IMI was less than 6 months prior to PHE (OR=3.78, 95% CI=0.98 to 14.56,

For peer review only - http://bmjopen.bmj.com/site/about/guidelines.xhtml

p=0.05). However, the very wide CIs indicated considerable uncertainty about the true OR. No other significant candidate factors were observed.

Multivariable analyses

Table 4 shows the results of the multivariable analyses, where the adjusted prognostic value was evaluated for all PHE-derived variables. After adjustment for height and weight, age remained significantly associated with increased odds of sustaining an I-IMI during a season (OR=1.12 for a 1-year increase, 95%CI = 1.05 to 1.18, $p<0.001$) and a linear association was the best fit for this variable. However, there was no evidence that height and weight were strong prognostic factors independent of age.

After adjustment for age, height and weight, if the most recent hamstring IMI was more than 12 months but less than 3 years prior to PHE, the significant association and wide CI also remained (OR= 2.24, 95% CI = 1.11 to 4.53, p=0.02). However, no other candidates demonstrated prognostic importance. For most variables, the magnitude of the adjusted prognostic association was also smaller than the unadjusted association and some confidence intervals were very wide.

For peer review only - http://bmjopen.bmj.com/site/about/guidelines.xhtml

Table 4: Univariable and multivariable logistic regression estimates for all candidate prognostic factors

	Univariable (unadjusted)				Multivariable (adjusted for age, height, weight)			
**Candidate PF & Type**	OR	95% CI	P	Best model fit	OR	95% CI	P	Best model fit
**Anthropometric (Adjustment Factors):**								
1: Age (years)	**1.12**	**1.06-1.18**	**<0.001**	**Linear**	**1.12**	**1.05 to 1.18**	**<0.001**	**Linear**
2: Height (cm)	1.03	0.99 to 1.07	0.13	-	1.02	0.97 to 1.07	0.46	-
3: Weight (kg)	**1.03**	**1.00 to 1.07**	**0.03**	**Linear**	1.00	0.95 to 1.04	0.91	-
**Within 3 years preceding PHE, frequency of:**								
4: foot/ankle injuries	1.04	0.87 to 1.24	0.68	-	1.04	**0.87 to 1.25**	0.65	-
5: hip/groin injuries	1.16	0.90 to 1.51	0.25	-	1.29	0.99 to 1.70	0.06	-
6: knee injuries	0.96	0.72 to 1.29	0.81	-	0.98	0.72 to 1.32	0.88	-
7: shoulder injuries	2.38	0.98 to 5.75	0.05	-	1.89	0.76 to 4.74	0.17	-
8: lumbar spine injuries	0.88	0.60 to 1.29	0.50	-	1.08	0.72 to 1.61	0.71	-
9: iliopsoas IMIs	0.73	0.38 to 1.43	0.37	-	0.86	0.43 to 1.71	0.67	-
10: hip adductor IMIs	1.38	0.92 to 2.09	0.12	-	1.18	0.76 to 1.84	0.46	-
11: hamstring IMIs	**1.56**	**1.17 to 2.09**	**<0.001**	**Linear**	1.35	1.00 to 1.83	0.05	-
12: quadriceps IMIs	1.08	0.67 to 1.73	0.75	-	1.05	0.65 to 1.71	0.84	-
13: calf IMIs	**1.80**	**1.09 to 2.97**	**0.02**	**Linear**	1.30	0.75 to 2.25	0.35	-
**Within 3 years preceding PHE, most recent:**								
14: foot/ankle injury (never)	ref	ref	ref	-	ref	ref	ref	-
14: foot/ankle injury (<6 months)	1.27	0.64 to 2.53	0.49	-	1.41	0.69 to 2.87	0.35	-
14: foot/ankle injury (6-12 months)	1.16	0.54 to 2.46	0.71	-	1.15	0.52 to 2.52	0.73	-
14: foot/ankle injury (>12 months)	1.27	0.75 to 2.13	0.37	-	1.22	0.71 to 2.09	0.47	-
15: hip/groin injury (never)	ref	ref	ref	-	ref	ref	ref	-
15: hip/groin injury (<6 months)	1.05	0.44 to 2.49	0.92	-	1.66	0.67 to 4.13	0.27	-
15: hip/groin injury (6-12 months)	0.59	0.23 to 1.50	0.27	-	0.65	0.25 to 1.69	0.38	-
15: hip/groin injury (>12months)	1.58	0.87 to 2.87	0.14	-	1.80	0.96 to 3.36	0.07	-
16: knee injury (never)	ref	ref	ref	-	ref	ref	ref	-
16: knee injury (<6 months)	1.15	0.40 to 3.28	0.80	-	1.12	0.37 to 3.34	0.84	-
16: knee injury (6-12 months)	1.23	0.58 to 2.62	0.60	-	1.24	0.57 to 2.70	0.60	-
16: knee injury (>12months)	0.93	0.53 to 1.61	0.79	-	0.99	0.56 to 1.75	0.96	-
17: shoulder injury (never)	ref	ref	ref	-	ref	ref	ref	-
17: shoulder injury (<6 months)	2.75	0.50 to 15.26	0.25	-	2.55	0.44 to 14.72	0.30	-
17: shoulder injury (6-12 months)	1.38	0.19 to 9.90	0.75	-	1.11	1.15 to 8.36	0.92	-
17: shoulder injury (>12months)	3.21	0.81 to 12.67	0.10	-	2.36	0.58 to 9.62	0.23	-
18: lumbar spine injury (never)	ref	ref	ref	-	ref	ref	ref	-
18: lumbar spine injury (<6 months)	1.24	0.30 to 5.05	0.77	-	2.10	0.50 to 8.82	0.31	-
18: lumbar spine injury (6-12 months)	0.62	0.15 to 2.53	0.50	-	0.75	0.18 to 3.14	0.69	-
18: lumbar spine injury (>12months)	0.70	0.34 to 1.44	0.33	-	0.97	0.46 to 2.06	0.94	-
19: iliopsoas IMI (never)	ref	ref	ref	-	ref	ref	ref	-
19: iliopsoas IMI (<6 months)	1.24	0.77 to 20.05	0.88	-	1.27	0.08 to 21.32	0.87	-
19: iliopsoas IMI (6-12 months)	0.62	0.15 to 2.53	0.51	-	0.83	0.20 to 3.43	0.80	-
19: iliopsoas IMI (>12months)	0.57	0.21 to 1.55	0.27	-	0.66	0.24 to 1.82	0.42	-
20: hip adductor IMI (never)	ref	ref	ref	-	ref	ref	ref	-
20: hip adductor IMI (<6 months)	1.37	0.53 to 3.56	0.52	-	1.29	0.48 to 3.47	0.61	
20: hip adductor IMI (6-12 months)	1.37	0.43 to 4.36	0.60	-	1.16	0.35 to 3.81	0.81	-
20: hip adductor IMI (>12months)	1.37	0.59 to 3.16	0.46	-	1.14	0.47 to 2.79	0.77	-
21: hamstring IMI (never)	ref	ref	ref	-	ref	ref	ref	-
21: hamstring IMI (<6 months)	2.84	0.81 to 9.99	0.10	-	2.07	0.57 to 7.56	0.27	-
21: hamstring IMI (6-12 months)	1.42	0.66 to 3.06	0.37	-	1.22	0.56 to 2.70	0.62	-
21: hamstring IMI (>12months)	**2.95**	**1.51 to 5.73**	**<0.001**	**Linear**	**2.24**	**1.11 to 4.53**	**0.02**	**Linear**
22: quadriceps IMI (never)	ref	ref	ref	-	ref	ref	ref	-
22: quadriceps IMI (<6 months)	1.74	0.38 to 7.91	0.48	-	1.56	0.33 to 7.36	0.58	-
22: quadriceps IMI (6-12 months)	0.58	0.17 to 1.93	0.37	-	0.59	0.17 to 2.04	0.41	-
22: quadriceps IMI (>12months)	1.14	0.53 o 2.43	0.74	-	1.07	0.49 to 2.35	0.86	-
23: calf IMI (never)	ref	ref	ref	-	ref	ref	ref	-
23: calf IMI (<6 months)	3.78	0.98 to 14.56	0.05	-	3.11	0.78 to 12.46	0.11	-
23: calf IMI (6-12 months)	7.09	0.82 to 61.51	0.08	-	3.80	0.41 to 35.41	0.24	-
23: calf IMI (>12months)	1.26	0.47 to 3.36	0.64	-	0.73	0.24 to 2.15	0.56	-
**Musculoskeletal:**								
24. Mean PROM hip IR (deg.)	**0.97**	**0.95 to 0.99**	**0.01**	**Linear**	0.98	0.95 to 1.00	0.06	-
25. Mean PROM hip ER (deg.)	0.97	0.95 to 1.00	0.09	-	0.99	0.96 to 1.02	0.53	-
26. Mean hip flexor length (deg.)	1.01	0.98 to 1.04	0.46	-	1.01	0.99 to 1.04	0.32	-

For peer review only

27. Mean hamstring/neural mobility length (deg.)	0.99	0.96 to 1.02	0.53	-	0.98	0.95 to 1.02	0.33	-
28. Mean calf muscle length (deg.)	1.00	0.97 to 1.02	0.77	-	1.00	0.97 to 1.02	0.79	-
**Strength/Power:**								
29: Max. leg extension power difference (W/kg$^{-0.67}$)	0.99	0.92 to 1.07	0.84	-	0.99	0.91 to 1.06	0.71	-
30: Mean of max. leg extension power (W/kg$^{-0.67}$)	1.02	0.99 to 1.05	0.24	-	1.01	0.97 to 1.04	0.73	-
31: Max. leg extension velocity difference (m.s^{-1})	2.15	0.31 to 14.88	0.44	-	2.83	0.38 to 21.31	0.31	-
32: Mean of max. leg extension velocity (m.s^{-1})	1.96	0.68 to 5.64	0.21	-	1.48	0.49 to 4.47	0.49	-
33: Max leg extension force difference (N/kg$^{-0.67}$)	0.99	0.98 to 1.02	0.76	-	1.00	0.98 to 1.02	0.65	-
34: Mean of max. leg extension force (N/kg$^{-0.67}$)	1.00	0.99 to 1.01	1.00	-	1.00	0.99 to 1.01	0.72	-
35: CMJ Force per kg of body mass (N/kg)	0.99	0.93 to 1.06	0.78	-	0.99	0.92 to 1.07	0.80	-
36: CMJ height (cm)	1.03	0.98 to 1.08	0.27	-	0.72	0.96 to 1.07	0.63	-

Key: BMI= body mass index; cm = centimetres; CI=confidence interval; CMJ=countermovement jump; deg. = degrees; Freq= frequency; IMI= indirect muscle injury; I-IMI=index indirect muscle injury; Kg=kilograms; kg/m^2 = kilograms/body height (metres) squared; *max.=maximum; m.s.= metres per second; N= newtons (note: N/kg$^{-0.67}$ has a scaling factor to normalise force to body mass[45]); OR=odds ratio; PF= prognostic factor; PHE= periodic health examination; PROM=passive range of movement; ref=reference category; SLR= straight leg raise; W= watts (note: W/kg$^{-0.67}$ has a scaling factor to normalise force to body mass[45]); WBL=weight bearing lunge; - = not applicable.*

Note: *ORs are expressed per one-unit increase for all continuous factors, and according to category for all categorical factors; Factors in* ***bold*** *indicate significance at the 0.05 level.*

For peer review only

Complete case and sensitivity analysis

The results of all complete case and sensitivity analyses are presented in Supplementary Files 6-8. Supplementary File 9 shows a forest plot of the estimates obtained for all statistically significant candidate prognostic factors across all primary and sensitivity univariable analyses. Supplementary File 10 shows a forest plot of the estimates obtained for all statistically significant candidate prognostic factors across all multivariable analyses.

For both univariable and multivariable analyses, the prognostic associations were very similar for the complete case and imputation analyses. Sensitivity analyses (that is, excluding players who were loaned or transferred) led to slightly stronger prognostic associations for some factors (for example, the frequency and timing of previous hamstring injuries), although the CIs were wider.

For peer review only - http://bmjopen.bmj.com/site/about/guidelines.xhtml

DISCUSSION

This exploratory study has examined linear and non-linear prognostic associations between PHE-derived variables and I-IMIs in elite football players, using routinely collected data from a 5-season period.

While the univariable analyses suggested 6 candidate factors are associated with I-IMIs, such analyses are limited as they only provide baseline association measures prior to adjustment for other important prognostic factors.[26] However, after adjustment in the multivariable analyses, the evidence indicates most PHE derived variables did not add any prognostic value over and above age. The only exception was that if a hamstring IMI was sustained by a player more than 12 months (but less than 3 years) prior to PHE, their odds of sustaining a lower extremity I-IMI significantly increased 2.2-fold, which has not been previously reported. Although not directly comparable, earlier studies have also shown that a history of a previous hamstring IMI is specifically associated with an increased hazard of future hamstring IMIs in elite players.[14, 17, 19] Nevertheless, the uncertainty in our estimates (demonstrated by wide 95% confidence intervals) and differences observed during the sensitivity analyses mean that this variable only has provisional prognostic value and needs to be established in further confirmatory studies.

Indeed, age was the only variable that could be considered as an important prognostic factor, which is easily obtained even without conducting PHE. For illustration, our estimates suggest that for every 1-year increase in age, the odds of sustaining an I-IMI during a season would increase by approximately 12%. As an example, to put into the context of absolute risk, for two players who were the same height and weight but aged 5 years apart, if the younger-aged

For peer review only - http://bmjopen.bmj.com/site/about/guidelines.xhtml

player had a risk of 0.44 (which was overall outcome prevalence in our study), then the older player would have a risk of 0.58.

The findings of this present study confirm those of a previous study that developed and validated a multivariable prognostic model to predict general lower extremity IMI risk in elite football players using PHE data, where age was considered an important prognostic factor (OR = 1.10, 95% CI = 1.03 to 1.17).[13] Other studies have shown that age is a multivariable prognostic factor specifically associated with increased hamstring IMI risk (OR range 1.40-1.78), [14, 15, 20] although the reported estimates were larger than those observed in our study. These differences may be due to chance or partly because we merged all lower extremity I-IMI outcomes rather than utilising IMI subgroups, which may have diluted the strength of our observed associations. However, although our approach was less clinically meaningful, merging I-IMI outcomes was essential in order to maximise the statistical power of our study.

Importantly, while its prognostic importance has been confirmed in multiple studies[13-15, 20] age is not a causal factor for future IMI occurrence. Rather, it is likely to be a proxy marker for another potential causal mechanism. Taking this and the non-modifiable nature of age into account, this factor could not be used clinically to inform specific injury mitigation interventions, so should only be considered useful to explain differences in risk between players in a team, or included in future prognostic model development studies.

Using data from PHE tests that measure modifiable physical and performance characteristics has been previously questioned for injury prediction purposes.[2] Our results fully support this view, because none of the modifiable musculoskeletal (clinical examination) or strength

For peer review only - http://bmjopen.bmj.com/site/about/guidelines.xhtml

and power tests evaluated showed any statistically significant associations with I-IMIs. This absence of strong associations mean that such tests have poor discriminatory ability, usually because of overlap in test scores that occur in individuals who sustain a future injury and those who do not.[2] Furthermore, after measurement at a solitary timepoint (i.e. pre-season), it is likely that the prognostic value of modifiable factors is time-varying[52] as a consequence of physical and physiological adaptations that occur from training exposure and other injuries.[53]

Overall, when considering the findings of this study and the related previous prognostic model development and validation study,[13] the majority of PHE derived candidate prognostic factors cannot be considered useful for IMI risk prediction and injury prevention practice in elite football players. However, because of this study's exploratory nature (with many estimates having very wide confidence intervals), the shortcomings of the current evidence base and the paucity of known prognostic factors in elite football,[14] there is a clear need for further investigation in this area to improve our understanding of the prognostic value of PHE in elite football and other sports.

Limitations and future research

This study is unique in that we have investigated non-linear associations as per methodological guidelines.[12, 54] However, in the analyses, non-linear associations were not found to be superior to linear associations. For practical reasons, our imputation model did not assume non-linear associations and therefore may have reduced the ability to detect genuine non-linear relationships in the subsequent analyses. However, this is not a concern for age, as there were no missing values for this factor and is unlikely to be a material concern for all other factors as missing data was always less than 15%.

For peer review only - http://bmjopen.bmj.com/site/about/guidelines.xhtml

A competing risks analysis was not conducted, which meant that individuals who sustained injury types other than lower extremity IMIs were still considered at risk, even though this may have affected their training and match exposure and hence risk of sustaining an I-IMI event. Candidate factors were only measured at one timepoint each season, which means that dynamic associations were not investigated. We also assumed that participant-seasons were independent. Future studies could account for competing risks, utilise repeated measurements over time (i.e. using intermittent PHE, conducted at various stages throughout the season) and incorporate between-season correlations into analyses. However, the complexity such analyses would also require a significantly larger volume of data. This could be achieved through data sharing initiatives and individual participant data meta-analysis, which would also increase the power to detect genuine prognostic associations and non-linear relationships.

Finally, it is acknowledged that the PHE data used in this study and the related prognostic model development study was restricted to 60 candidate prognostic factors overall, from a limited selection of PHE procedures.[13, 25] Further studies should investigate a wider selection of PHE tests, including (but not limited to) other musculoskeletal, biomechanical, imaging and other in vivo diagnostic tests for example, providing that the quality of data is robust through evaluation of reliability and validity.

CONCLUSION

This study has evaluated prognostic associations between PHE-derived candidate variables and lower extremity I-IMIs in elite football players using data that were routinely collected over 5 seasons. No clear associations were found for nearly all PHE variables, although if a

For peer review only - http://bmjopen.bmj.com/site/about/guidelines.xhtml

player sustained a hamstring IMI greater than 12 months (but less than 3 years) prior to PHE, then this had potential prognostic value over and above the prognostic value of age. Indeed, age was the only variable to be confirmed as a clear prognostic factor in both univariable and multivariable analyses. However, this is easily measured without the need to conduct PHE, and although it has limited application in clinical practice, it should be included as an important factor in any future prognostic model development studies. Overall, the PHE processes used in this study and the related prognostic model development and validation study cannot be currently considered as a useful source of prognostic factors for I-IMI risk prediction and injury prevention practice in elite football players. Instead, they should only be considered potentially useful for screening of pathology, as well as rehabilitation and performance monitoring. Further research is required to confirm the prognostic value of PHE for IMI risk prediction and to identify novel prognostic factors that could improve development of prognostic models in football and other sports.

ACKNOWLEDGEMENTS

The authors would like to thank all staff within the Medical and Sports Science Department at Manchester United for their continuing help and support with this manuscript and thank all players for their participation (without whom this study would not be possible). The authors also thank Versus Arthritis for their support: Versus Arthritis grant number 21755.

CONTRIBUTORS

TH was responsible for the conceptualisation of the project, study design, database construction, data extraction and cleaning, protocol development and protocol writing. TH conducted the data analysis, interpretation and wrote the main manuscript. RR provided statistical guidance and assisted with development of the study design, analysis, and edited

For peer review only - http://bmjopen.bmj.com/site/about/guidelines.xhtml

manuscript drafts. MC assisted with the study conceptualisation and design, protocol development, clinical interpretation and editing the manuscript drafts. JS provided guidance with the study design, development of the analysis and protocol, interpretation of the analysis, as well as editing the study manuscripts. All authors read and approved this final manuscript.

CONFLICT OF INTEREST

None declared.

FUNDING

The lead researcher (TH) is receiving sponsorship from Manchester United Football Club to complete a postgraduate PhD study programme. This work was also supported by Versus Arthritis: grant number 21755.

ETHICS AND DATA USE

Informed consent was not required as data were captured from the mandatory PHE completed through the participants' employment. The data usage was approved by the football club and the Research Ethics Service at the University of Manchester.

DATA SHARING STATEMENT

An anonymised summary of the dataset that was analysed during this study may be available from the corresponding author on reasonable request.

PATIENT CONSENT FOR PUBLICATION

Not required.

For peer review only

References:

1 McCall A, Carling C, Davison M*, et al.* Injury risk factors, screening tests and preventative strategies: a systematic review of the evidence that underpins the perceptions and practices of 44 football (soccer) teams from various premier leagues. *Br J Sports Med* 2015;**49**:583-9.

2 Bahr R. Why screening tests to predict injury do not work-and probably never will...: a critical review. *Br J Sports Med* 2016;**50**:776-80.

3 Ljungqvist A, Jenoure PJ, Engebretsen AH*, et al.* The International Olympic Committee (IOC) Consensus Statement on Periodic Health Evaluation of Elite Athletes, March 2009. *Clin J Sport Med* 2009;**19**:347-60.

4 Hughes T, Sergeant JC, van der Windt DA*, et al.* Periodic Health Examination and Injury Prediction in Professional Football (Soccer): Theoretically, the Prognosis is Good. *Sports Med* 2018;**48**:2443-48.

5 Ekstrand J, Hagglund M, Walden M. Epidemiology of muscle injuries in professional football (soccer). *Am J Sports Med* 2011;**39**:1226-32.

6 Falese L, Della Valle P, Federico B. Epidemiology of football (soccer) injuries in the 2012/2013 and 2013/2014 seasons of the Italian Serie A. *Res Sports Med* 2016;**24**:426-32.

7 Larruskain J, Lekue JA, Diaz N*, et al.* A comparison of injuries in elite male and female football players: A five-season prospective study. *Scand J Med Sci Sports* 2018;**28**:237-45.

8 Leventer L, Eek F, Hofstetter S*, et al.* Injury Patterns among Elite Football Players: A Media-based Analysis over 6 Seasons with Emphasis on Playing Position. *Int J Sports Med* 2016;**37**:898-908.

9 Hawkins RD, Fuller CW. A prospective epidemiological study of injuries in four English professional football clubs. *Br J Sports Med* 1999;**33**:196-203.

10 Steyerberg EW, Moons KG, van der Windt DA*, et al.* Prognosis Research Strategy (PROGRESS) 3: prognostic model research. *PLoS Med* 2013;**10**:e1001381.

11 Riley RD, Hayden JA, Steyerberg EW*, et al.* Prognosis Research Strategy (PROGRESS) 2: prognostic factor research. *PLoS Med* 2013;**10**:e1001380.

12 Riley RD, van der Windt DA, Croft P*, et al. Prognosis Research in Healthcare: Concepts, Methods and Impact*. Oxford: Oxford University Press 2019.

13 Hughes T, Riley RD, Callaghan MJ*, et al.* The Value of Preseason Screening for Injury Prediction: The Development and Internal Validation of a Multivariable Prognostic Model to Predict Indirect Muscle Injury Risk in Elite Football (Soccer) Players. *Sports Medicine - Open* 2020;**6**.

For peer review only - http://bmjopen.bmj.com/site/about/guidelines.xhtml

14 Hughes T, Sergeant JC, Parkes M*, et al.* Prognostic factors for specific lower extremity and spinal musculoskeletal injuries identified through medical screening and training load monitoring in professional football (soccer): a systematic review. *BMJ Open Sport Exercise Med* 2017;**3**:1-18.

15 Arnason A, Sigurdsson SB, Gudmundsson A*, et al.* Risk factors for injuries in football. *Am J Sports Med* 2004;**32**:5S-16S.

16 Fousekis K, Tsepis E, Poulmedis P*, et al.* Intrinsic risk factors of non-contact quadriceps and hamstring strains in soccer: a prospective study of 100 professional players. *Br J Sports Med* 2011;**45**:709-14.

17 Hägglund M, Waldén M, Ekstrand J. Risk Factors for Lower Extremity Muscle Injury in Professional Soccer: The UEFA Injury Study. *Am J Sports Med* 2013;**41**:327-35.

18 Timmins RG, Bourne MN, Shield AJ*, et al.* Short biceps femoris fascicles and eccentric knee flexor weakness increase the risk of hamstring injury in elite football (soccer): a prospective cohort study. *Br J Sports Med* 2016;**50**:1524-35.

19 Hägglund M, Waldén M, Ekstrand J. Previous injury as a risk factor for injury in elite football: a prospective study over two consecutive seasons. *Br J Sports Med* 2006;**40**:767-72.

20 Henderson G, Barnes CA, Portas MD. Factors associated with increased propensity for hamstring injury in English Premier League soccer players. *J Sci Med Sport* 2010;**13**:397-402.

21 van Dyk N, Bahr R, Whiteley R*, et al.* Hamstring and Quadriceps Isokinetic Strength Deficits Are Weak Risk Factors for Hamstring Strain Injuries: A 4-Year Cohort Study. *Am J Sports Med* 2016;**44**:1789-95.

22 Moons KG, Altman DG, Reitsma JB*, et al.* Transparent Reporting of a multivariable prediction model for Individual Prognosis or Diagnosis (TRIPOD): explanation and elaboration. *Ann Intern Med* 2015;**162**:W1-73.

23 Royston P, Altman DG, Sauerbrei W. Dichotomizing continuous predictors in multiple regression: a bad idea. *Stat Med* 2006;**25**:127-41.

24 Altman DG, Royston P. The cost of dichotomising continuous variables. *Br Med J* 2006;**332**:1080.

25 Hughes T, Riley R, Sergeant J*, et al.* A study protocol for the development and internal validation of a multivariable prognostic model to determine lower extremity muscle injury risk in elite football (soccer) players, with further exploration of prognostic factors. *Diagn Progn Res* 2019;**3**.

26 Sauerbrei W, Taube SE, McShane LM*, et al.* Reporting Recommendations for Tumor Marker Prognostic Studies (REMARK): An Abridged Explanation and Elaboration. *J Natl Cancer Inst* 2018;**110**:803-11.

For peer review only - http://bmjopen.bmj.com/site/about/guidelines.xhtml

27 Altman DG, Lyman GH. Methodological challenges in the evaluation of prognostic factors in breast cancer. *Breast Cancer Res Treat* 1998;**52**:289-303.

28 Fuller CW, Ekstrand J, Junge A*, et al.* Consensus statement on injury definitions and data collection procedures in studies of football (soccer) injuries. *Br J Sports Med* 2006;**40**:193-201.

29 Mueller-Wohlfahrt HW, Haensel L, Mithoefer K*, et al.* Terminology and classification of muscle injuries in sport: the Munich consensus statement. *Br J Sports Med* 2013;**47**:342-50.

30 Ekstrand J, Askling C, Magnusson H*, et al.* Return to play after thigh muscle injury in elite football players: implementation and validation of the Munich muscle injury classification. *Br J Sports Med* 2013;**47**:769-74.

31 Hsieh FY, Block DA, Larsen MD. A simple method of sample size calculation fro linear and logistic regression. *Stat Med* 1998;**17**:1623-34.

32 Coppieters M, Stappaerts K, Janssens K*, et al.* Reliability of detecting 'onset of pain' and 'submaximal pain' during neural provocation testing of the upper quadrant. *Physiother Res Int* 2002;**7**:146-56.

33 Roach S, San Juan JG, Suprak DN*, et al.* Concurrent validity of digital inclinometer and universal goniometer assessing passive hip mobility in healthy subjects. *The Int J Sports Phys Ther* 2013;**8**:680-8.

34 Clapis PA, Davis SM, Davis RO. Reliability of inclinometer and goniometric measurements of hip extension flexibility using the modified Thomas test. *Phys Theory Pract* 2008;**24**:135-41.

35 Fraeulin L, Holzgreve F, Brinkbaumer M*, et al.* Intra- and inter-rater reliability of joint range of motion tests using tape measure, digital inclinometer and inertial motion capturing. *PLoS One* 2020;**15**:e0243646.

36 Gabbe BJ, Bennell KL, Wajswelner H*, et al.* Reliability of common lower extremity musculoskeletal screening tests. *Phys Ther Sport* 2004;**5**:90-7.

37 Boyd BS. Measurement properties of a hand-held inclinometer during straight leg raise neurodynamic testing. *Physiotherapy* 2012;**98**:174-9.

38 Williams CM, Caserta AJ, Haines TP. The TiltMeter app is a novel and accurate measurement tool for the weight bearing lunge test. *J Sci Med Sport* 2013;**16**:392-5.

39 Munteanu SE, Strawhorn AB, Landorf KB*, et al.* A weightbearing technique for the measurement of ankle joint dorsiflexion with the knee extended is reliable. *J Sci Med Sport* 2009;**12**:54-9.

40 Hall EA, Docherty CL. Validity of clinical outcome measures to evaluate ankle range ofmotion during the weight-bearing lunge test. *J Sports Sci Med* 2017;**20**:618-21
.

For peer review only - http://bmjopen.bmj.com/site/about/guidelines.xhtml

41 Redden J, Stokes K, Williams S. Establishing the Reliability and Limits of Meaningful Change of Lower Limb Strength and Power Measures during Seated Leg Press in Elite Soccer Players. *J Sports Sci Med* 2018;**17**:539-46.

42 Lindberg K, Erthorsdottir I, Solberg P*, et al.* Validity of Force–Velocity Profiling Assessed With a Pneumatic Leg Press Device. *Int J Sports Physiol Perform* 2021;**16**:1777-85.

43 Slinde F, Suber C, Suber L*, et al.* Test–Retest Reliability Of Three Different Countermovement Jumping Tests. *J Strength Cond Res* 2008;**22**:640-3.

44 Lake J, Mundy P, Comfort P*, et al.* Concurrent Validity of a Portable Force Plate Using Vertical Jump Force–Time Characteristics. *J Appl Biomech* 2018;**34**:410-3.

45 Jaric S. Muscle Strength Testing. Use of Normalisation for Body Size. *Sports Med* 2002;**32**:615-31.

46 Lunt M. nscore. Manchester University of Manchester 2007.

47 Sterne JAC, White IR, Carlin J*, et al.* Multiple imputation for missing data in epidemiological and clinical research: potential and pitfalls. *Br Med J* 2009;**338**:b2393.

48 Marshall A, Altman DG, Holder RL*, et al.* Combining estimates of interest in prognostic modelling studies after multiple imputation: current practice and guidelines. *BMC Med Res Methodol* 2009;**9**:57.

49 Bland M. *An Introduction to Medical Statistics*. New York: Oxford University Press 2015.

50 Royston P, Ambler G, Sauerbrei W. The Use of Fractional Polynomials to Model Continuous Risk Variables in Epidemiology. *Int J Epidemiol* 1999;**28**:964-74.

51 Morris TP, White IR, Carpenter JR*, et al.* Combining fractional polynomial model building with multiple imputation. *Stat Med* 2015;**34**:3298-317.

52 Bansal A, Heagerty PJ. A comparison of landmark methods and time-dependent ROC methods to evaluate the time-varying performance of prognostic markers for survival outcomes. *Diagn Progn Res* 2019;**3**:14.

53 Meeuwisse W, Tyreman H, Hagel B*, et al.* A Dynamic Model of Etiology in Sport Injury: The Recursive Nature of Risk and Causation. *Clin J Sports Med* 2007;**17**:215-9.

54 Riley RD, Sauerbrei W, Altman DG. Prognostic markers in cancer: the evolution of evidence from single studies to meta-analysis, and beyond. *Br J Cancer* 2009;**100**:1219-29.

For peer review only - http://bmjopen.bmj.com/site/about/guidelines.xhtml

Figure caption and legend:

Figure 1: Participant flow chart

Key: n = number of participants; I-IMI = index indirect muscle injury

For peer review only

For peer review only - http://bmjopen.bmj.com/site/about/guidelines.xhtml

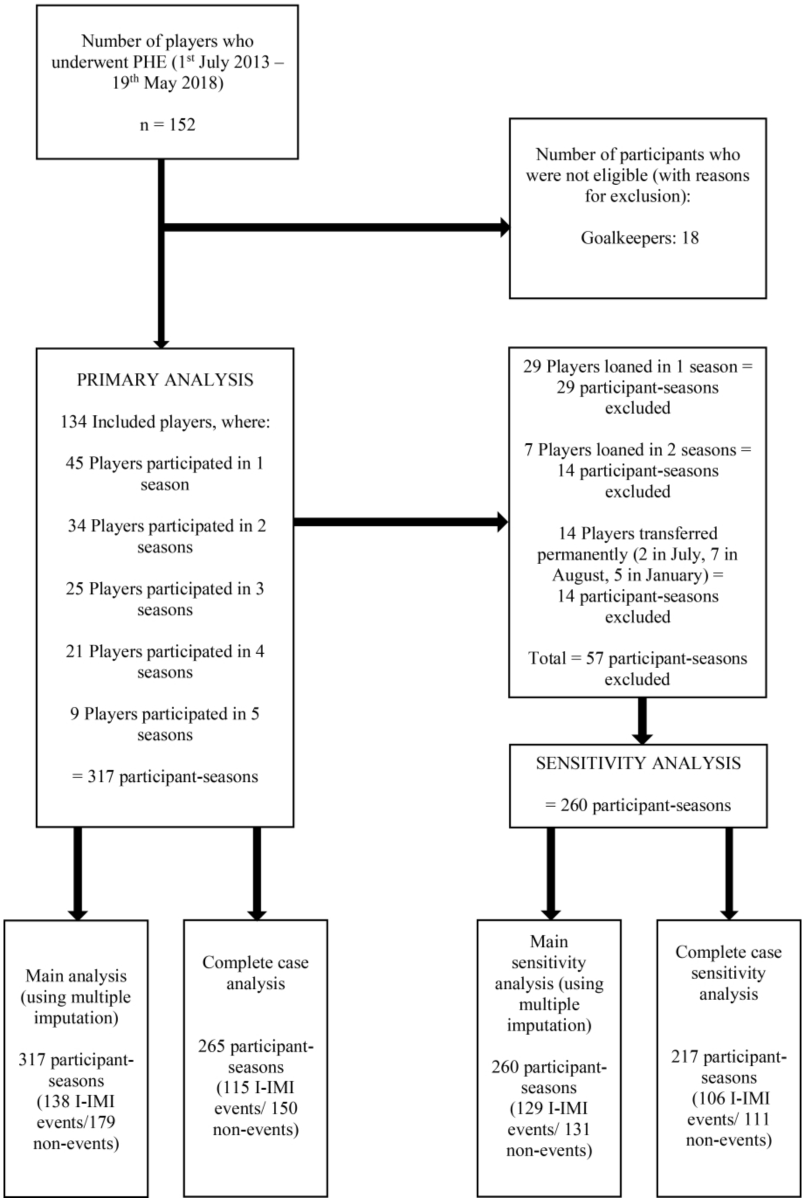

Figure 1: Participant flow chart

Key: I-IMI=index indirect muscle injury; n=number of participants

Does preseason screening provide a source of potential prognostic factors for indirect muscle injuries in elite football (soccer) players? An exploratory analysis using routinely-collected periodic health examination data

Hughes, T., Riley, R.D., Callaghan, M.J. and Sergeant, J.C. (2022)

For peer review only

Supplementary file 1: Detailed descriptions of all PHE tests.

Anthropometric measurements

For each participant, height was recorded in centimetres (cm) and body weight was measured in kilograms (kg) using a combined weight and height scales (Seca, Hamburg, Germany). Body mass index was calculated as weight (kg) divided by the squared height (metres). Body compositions were estimated through percentage body fat calculations; using a generic skinfold calliper, measurements were taken from the of triceps, subscapular, lower abdominal, anterior thigh, pectoral and iliac crest anatomical locations by the same examiner.

Past Medical/Injury history

All participants underwent an interview with a club medical doctor to discuss their past medical and injury history. For participants who were transferred to the club during the data collection period, their initial PHE included questions regarding family medical history and any previous significant injuries sustained at their previous club (which resulted in time lost to training of > 7-10 days). This information was cross referenced with their medical records received from the previous football club. Subsequent PHEs consisted of an interview which reviewed the medical information and injury history that was routinely recorded on the medical notes system during each participant's employment with the club.

Musculoskeletal examination

Participants underwent a series of musculoskeletal tests that were completed by one examiner, while another examiner monitored the procedure for any accessory or compensatory movements and verified the measurement before recording it into a database. If any accessory or compensatory movements were noted the procedure for that particular test was repeated. All examination procedures were performed according to a standardised protocol.

For peer review only

Passive Hip Internal/External Rotation

Hip rotational range of movement was used to evaluate hip joint mobility.(1) Participants laid supine with both thighs supported by the treatment plinth and knees flexed over the end of the plinth to allow the lower legs to hang freely. A digital inclinometer (Digi-Pas Uk – Tarax Technology Ltd, Cambridge) was placed on the medial aspect of the tibia immediately proximal to the medial malleolus of the tibia. The examiner instructed the participant to flex the contralateral hip and knee and place the foot on the plinth to stabilise the pelvis. The hip was passively rotated to the maximal range of external rotation, until resistance prevented any further movement or symptoms were experienced. The range was documented in degrees and entered into the database. The test was repeated for passive internal rotation. Both procedures were repeated on the contralateral limb. Use of a digital inclinometer to evaluate hip rotation has previously been shown to have excellent intra-rater (intraclass correlation coefficient (ICC)= 0.84- 0.90)(1, 2), inter-rater (ICC=0.89-0.93)(3) and test-retest reliability (ICC=0.93-0.96).(4)

Modified Thomas Test

The modified Thomas test was used to measure the flexibility of the anterior hip musculature.(5) Participants stood against a plinth which was adjusted so it was aligned to the height of the anterior superior iliac spine (ASIS). Participants lay supine on the edge of the plinth with both legs hanging freely. Both hips and knees were maximally flexed and held to the chest with both hands of the participant. One limb was released and the examiner extended the hip with as far as possible, whilst ensuring that the lumbar spine remained in contact with the plinth and the contralateral knee maintained the flexed position. A digital inclinometer (Digi-Pas Uk – Tarax Technology Ltd, Cambridge) was placed on the muscle belly of the upper 1/3 of the quadriceps and the measurement was recorded in degrees. If the femur was angled so it was above horizontal, a negative measurement was recorded. If it was below the horizontal, a positive measure was recorded. The procedure was then repeated on the contralateral leg. The inter-rater reliability of using an inclinometer with this test has be previously established as good (ICC= 0.89).(6)

For peer review only

Straight Leg Raise

The straight leg raise (SLR) was used to measure of hamstring and neurodynamic mobility.(5) The participant laid supine on a plinth. A digital inclinometer (Digi-Pas Uk – Tarax Technology Ltd, Cambridge) was placed on the anterior margin of the tibia (10 centimetres distal to the tibial plateau) by the examiner. The examiner maintained full knee extension and passively flexed the hip to its maximal range in the sagittal plane, or to the point where any symptoms were noted. The range was recorded in degrees and the test was repeated on the contralateral limb. Previously for this method of measurement, intra-rater reliability has been shown to be excellent (ICC 0.95-0.98) (7) and inter-rater reliability has been shown to be good (ICC 0.80-0.97). (8)

Weight Bearing Lunge

The weight bearing lunge (WBL) was used to measure dorsiflexion mobility of the ankle.(9) Participants were instructed to stand along a tape line which was perpendicular to the wall, where the second toe and centre of the heel were placed over the line. A digital inclinometer (Digi-Pas Uk – Tarax Technology Ltd, Cambridge) was placed over the anterior margin of the tibia (10 centimetres distal to the tibial plateau) in a neutral position. Participants were instructed to step as far forward as possible with the contralateral leg, into a forward stride position with both hands on the wall in front, which dorsiflexed the rear ankle. Participants were instructed to lean forward as much as possible until resistance or symptoms were experienced, whilst maintaining knee extension with contact of the heel on the floor. If the heel lifted from the ground the test was deemed as invalid and then repeated. The maximal range of dorsiflexion was documented in degrees. The test was repeated on the contralateral leg. Previously, this method has been shown to have good intra-rater reliability (ICC=0.88) and good to excellent inter-rater reliability (ICC 0.80- 0.95.(10, 11)

Strength and Power Tests

Maximal Loaded Leg Press

This test was used to measure the force, power and velocity for both lower limbs during an incremental leg double press test, which progressed from minimal to maximal resistance,

For peer review only

using a Keiser Air 300 machine (Keiser UK, Tetbury, Gloucestershire). Prior to this test participants warmed up on a Keiser M3 exercise cycle (Keiser UK, Tetbury, Gloucestershire) for 5 mins at resistance level one. Participants sat on the leg press machine and an appropriate seat position was selected, with the feet firmly rested on the footplates and the knees were at 90 degrees of flexion. Participants were instructed to generate as much force as possible, as quickly as possible by pushing against the footplate and keeping the spine flat against the seat during completion of the test movement. A test was deemed invalid if any compensatory movements were observed (such as elevating the pelvis from the seat to gain a mechanical advantage) and the test was repeated. The test consisted of repeated single repetitions, with each repetition increasing in resistance until the maximum resistance was reached and the participant could not complete the leg press movement. The measurements obtained for this test are peak power (Watts/kg(W/kg)), Peak velocity (metres/second (m/s)) and peak force (Newtons/kg (N/kg)) for each limb. The test-retest reliability has been shown to have good-excellent reliability for the power (ICC= 0.89), velocity (ICC= 0.79) and force (ICC=0.91) parameters. (12)

Countermovement jump (CMJ)

This test provided a functional measure of explosive power.(13, 14) All CMJs were performed on a dual plate force platform system (ForceDecks FD4000, ForceDecks, Salford, UK) and analysed using the manufacturer's dedicated software. The test procedure was explained and demonstrated to each participant. Participants were instructed to stand on the platform, with foot placement approximately hip width apart and their hands placed on the pelvis for the duration of the CMJ. In one dynamic movement, participants squatted to approximately 90 degrees of knee flexion before forcefully propelling vertically into the jump phase of the movement through triple extension of hips, knees and ankles, landing approximately in the original start position. Four CMJ repetitions were completed, although if jump height progressively increased with each effort, further CMJs were completed until height scores plateaued. If participants hands shifted position from the pelvis or the legs did not remain in an extended position during the take-off or flight phase then the repetition was deemed invalid and the test was repeated. The measurements taken from this test were peak jump height (cm), peak power (watts) and force per kg of body mass (N/kg). The test-retest reliability of peak jump height has been previously shown to be good (ICC=0.80-0.88).(15)

For peer review only

Similarly, for CMJ power, test-retest reliability has been shown to be excellent (ICC=0.92-0.98)(16).

References

1. Roach S, San Juan JG, Suprak DN, Lyda MA. Concurrent validity of digital inclinometer and universal goniometer assessing passive hip mobility in healthy subjects. The Int J Sport Phys Ther. 2013;8(5):680-8.

2. Krause DA, Hollman JH, Krych AJ, Kalisvaart MM, Levy BA. Reliability of hip internal rotation range of motion measurement using a digital inclinometer. Knee Surg Sports Traumatol Arthrosc. 2015;23(9):2562-7.

3. Hollman JH, Ginos BE, Kozuchowski J, Vaughn AS, Krause D, Youdas J. Relationships Between Knee Valgus, Hip-Muscle Strength, and Hip-Muscle Recruitment During a Single-Limb Step-Down. J Sport Rehab. 2009;18:104-17.

4. Pua YH, Wrigley TV, Cowan SM, Bennell KL. Intrarater test-retest reliability of hip range of motion and hip muscle strength measurements in persons with hip osteoarthritis. Arch Phys Med Rehabil. 2008;89(6):1146-54.

5. Magee DJ. Orthopedic Physical Assessment. 5th Ed. ed. Missouri: Saunders Elsevier; 2008.

6. Clapis PA, Davis SM, Davis RO. Reliability of inclinometer and goniometric measurements of hip extension flexibility using the modified Thomas test. Phys Theory Practice. 2008;24(2):135-41.

7. Boyd BS. Measurement properties of a hand-held inclinometer during straight leg raise neurodynamic testing. Physiotherapy. 2012;98(2):174-9.

8. Gabbe BJ, Bennell KL, Wajswelner H, Finch CF. Reliability of common lower extremity musculoskeletal screening tests. Phys Ther Sport. 2004;5(2):90-7.

9. Catalatayud J, Martin F, Gargallo P, Garcia-Redondo J, Colado JC, Marin PJ. The validity and reliability of a new instrumented device for measuring ankle dorsiflexion range of motion. Int J Sports Phys Ther. 2015;10(2):197-202.

10. Williams CM, Caserta AJ, Haines TP. The TiltMeter app is a novel and accurate measurement tool for the weight bearing lunge test. J Sci Med Sport. 2013;16(5):392-5.

11. Munteanu SE, Strawhorn AB, Landorf KB, Bird AR, Murley GS. A weightbearing technique for the measurement of ankle joint dorsiflexion with the knee extended is reliable. J Sci Med Sport. 2009;12(1):54-9.

For peer review only

12. Redden J, Stokes K, Williams S. Establishing the Reliability and Limits of Meaningful Change of Lower Limb Strength and Power Measures during Seated Leg Press in Elite Soccer Players. J Sports Sci Med. 2018;17:539-46.

13. Reiser RF, Rocheford EC, Armstrong CJ. Building a better understanding of basic mechanical prinicples through analysis of the vertical jump. Strength Cond J (Lippincott Williams & Wilkins). 2006;28(4):70-80.

14. Maulder P, Cronin J. Horizontal and vertical jump assessment: reliability, symmetry, discriminative and predictive ability. Phys Ther Sport. 2005;6(2):74-82.

15. Slinde F, Suber C, Suber L, Edwen CE, Svantesson U. Test–Retest Reliability Of Three Different Countermovement Jumping Tests. J Strength Cond Res. 2008;22(2):640-3.

16. Hori N, Newton RU, Kawamori N, McGuigan MR, Kraemer WJ, Nosaka K. Reliability of performance measurements derived from ground reaction force data during countermovement jump and the influence of sampling frequency. J Strength Cond Res (Allen Press Publishing Services Inc). 2009;23(3):874-82.

For peer review only

Does preseason screening provide a source of potential prognostic factors for indirect muscle injuries in elite football (soccer) players? An exploratory analysis using routinely-collected periodic health examination data

Hughes, T., Riley, R.D., Callaghan, M.J. and Sergeant, J.C. (2022)

Supplementary file 2: Sample size calculation

For peer review only

PASS 2019, v19.0.2 13/11/2019 14:40:18 1

Tests for the Odds Ratio in Logistic Regression with One Normal X and Other Xs (Wald Test)

Numeric Results

Power	N	P0	P1	Odds Ratio	R Squared	Alpha	Beta
0.79904	314	0.430	0.542	1.570	0.500	0.050	0.20096

References

Hsieh, F.Y., Block, D.A., and Larsen, M.D. 1998. 'A Simple Method of Sample Size Calculation for Linear and
Logistic Regression', Statistics in Medicine, Volume 17, pages 1623-1634.

Report Definitions

Power is the probability of rejecting a false null hypothesis. It should be close to one.
N is the size of the sample drawn from the population.
P0 is the response probability at the mean of X.
P1 is the response probability when X is increased to one standard deviation above the mean.
Odds Ratio is the odds ratio when P1 is on top. That is, it is [P1/(1-P1)]/[P0/(1-P0)].
R-Squared is the R2 achieved when X is regressed on the other independent variables in the regression.
Alpha is the probability of rejecting a true null hypothesis.
Beta is the probability of accepting a false null hypothesis.

Summary Statements

A logistic regression of a binary response variable (Y) on a continuous, normally distributed variable (X) with a sample size of 314 observations achieves 80% power at a 0.050 significance level to detect a change in Prob(Y=1) from the value of 0.430 at the mean of X to 0.542 when X is increased to one standard deviation above the mean. This change corresponds to an odds ratio of 1.570. An adjustment was made since a multiple regression of the independent variable of interest on the other independent variables in the logistic regression obtained an R-Squared of 0.500.

Dropout-Inflated Sample Size

Dropout Rate	Sample Size N	Dropout-Inflated Enrollment Sample Size N'	Expected Number of Dropouts D
20%	314	393	79

Definitions

Dropout Rate (DR) is the percentage of subjects (or items) that are expected to be lost at random during the
course of the study and for whom no response data will be collected (i.e. will be treated as "missing").
N is the evaluable sample size at which power is computed. If N subjects are evaluated out of the N' subjects
that are enrolled in the study, the design will achieve the stated power.
N' is the total number of subjects that should be enrolled in the study in order to end up with N

For peer review only

evaluable subjects, based on the assumed dropout rate. After solving for N, N' is calculated by inflating N using the formula N' = N / (1 - DR), with N' always rounded up. (See Julious, S.A. (2010) pages 52-53, or Chow, S.C., Shao, J., Wang, H., and Lokhnygina, Y. (2018) pages 32-33.)
D is the expected number of dropouts. D = N' - N.

For peer review only

Tests for the Odds Ratio in Logistic Regression with One Normal X and Other Xs (Wald Test)

Procedure Input Settings

Autosave Inactive

Design Tab

Solve For:	Sample Size
Alternative Hypothesis:	Two-Sided
Power:	0.80
Alpha:	0.05
P0 (Baseline Probability that Y=1):	0.43
Use P1 or Odds Ratio:	Odds Ratio
Odds Ratio (Odds1/Odds0):	1.57
R-Squared of X1 with Other X's:	0.5

For peer review only

Does preseason screening provide a source of potential prognostic factors for indirect muscle injuries in elite football (soccer) players? An exploratory analysis using routinely-collected periodic health examination data

Hughes, T., Riley, R.D., Callaghan, M.J. and Sergeant, J.C. (2022)

Supplementary file 3: Candidate prognostic factors that were excluded

For peer review only

Type of prognostic factor	Candidate Prognostic Factor	Composite variable created	Measurement unit	Measurement method	Data type	Reason for elimination
Anthropometric	Body fat	No	Percentage	Skin calipers	Continuous	Missing data > 15%
Musculoskeletal test	Quadriceps muscle length difference*	Yes	Degrees	Ely's test using digital inclinometer	Continuous	Intra-rater ICC = 0.69[1] Inter-rater ICC = 0.66. [1]
	Mean quadriceps muscle length**	Yes	Degrees	Ely's test using goniometer	Continuous	Intra-rater ICC = 0.69[1] Inter-rater ICC = 0.66. [1]
	Toe touch in standing	No	Centimetres	Fingertips to floor distance	Continuous	Missing data > 15%
	Sacroiliac joint kinematic function	No	Subjective score	Gillets test	Categorical	Missing data > 15%
Functional movement/balance tests	Y Balance Test – anterior translation difference*	Yes	Centimetres	Y Balance Test	Continuous	Missing data >15%
	Y Balance Test – Mean anterior translation**	Yes	Centimetres	Y Balance Test	Continuous	Missing data >15%
	Y Balance Test – posteromedial translation difference*	Yes	Centimetres	Y Balance Test	Continuous	Missing data >15%
	Y Balance Test – Mean posteromedial translation**	Yes	Centimetres	Y Balance Test	Continuous	Missing data >15%
	Y Balance Test –posterolateral translation difference*	Yes	Centimetres	Y Balance Test	Continuous	Missing data >15%
	Y Balance Test – Mean posterolateral translation**	Yes	Centimetres	Y Balance Test	Continuous	Missing data >15%
	R relative tibial angles	No	Degrees	SLS measurement with Dorsavi Viperform IMU	Continuous	Within-session ICCs=0.27-0.75 Between-session ICCs = 0.55-0.77[2]
	L relative tibial angles (left leg)	No	Degrees	SLS measurement with Dorsavi Viperform IMU	Continuous	Within-session ICCs= 0.27-0.75 Between-session ICCs = 0.55-0.77[2]
Strength/power tests	Upper body peak power difference*	Yes	Normalised watts per kilo ($W/kg^{-0.67}$)	Double horizontal press using a Keiser Chest Press Air 350 machine	Continuous	Missing data > 15%
	Mean upper body peak power**	Yes	Normalised watts per kilo ($W/kg^{-0.67}$)	Double horizontal press using a Keiser Chest Press Air 350 machine	Continuous	Missing data > 15%

Key: PHE=periodic health examination; WBL=weight bearing lunge; CMJ=countermovement jump; PROM=passive range of movement; ICC=intraclass correlation coefficient; SLR= straight leg raise; SLS = single leg squat; BMI= body mass index; f=force; W= watts; (note that $W/kg^{-0.67}$ *has a scaling factor to normalise power to body mass) Kg=kilos; IMU= inertial measurement units; m = mass; Note: composite factors are identified in the table with * denoting between limb differences and **denoting combined mean values of both limbs.*

References:

1. Peeler J, Anderson JE. Reliability of the Ely's test for assessing rectus femoris muscle flexibility and joint range of motion. *J Orth Res* 2008;26(6):793-9.

2. Hughes T, Jones RK, Starbuck C, et al. Are tibial angles measured with inertial sensors useful surrogates for frontal plane projection angles measured using 2-dimensional video analysis during single leg squat tasks? A reliability and agreement study in elite football (soccer) players. *J Electromyogr Kinesiol* 2019;44:21-30. doi: 10.1016/j.jelekin.2018.11.005

For peer review only

Does preseason screening provide a source of potential prognostic factors for indirect muscle injuries in elite football (soccer) players? An exploratory analysis using routinely-collected periodic health examination data

Hughes, T., Riley, R.D., Callaghan, M.J. and Sergeant, J.C. (2022)

Supplementary file 4: Anthropometric parameters and all included candidate prognostic factor characteristics for participants included in the sensitivity analyses

For peer review only

Characteristic/candidate prognostic factors		*Min*	*Lower quartile*	*Median*	*Upper quartile*	*Max.*	*Freq. (%) - if categorical*	*Missing values n (%)*
**Anthropometrics**								
1. Age at PHE (years)		16.01	17.64	19.20	24.30	39.59	-	0 (0)
2. Height (cm)		164.30	176.10	180.00	185.50	195.00	-	15 (5.77)
3. Weight (kg)		56.80	68.09	73.10	80.00	94.00	-	16 (6.15)
**Past medical history**								
Within 3 years prior to PHE, freq. of:								
4. foot/ankle injuries		0.00	0.00	1.00	2.00	7.00	-	0 (0)
5. Hip/groin injuries		0.00	0.00	0.00	1.00	5.00	-	
6. Knee injuries		0.00	0.00	0.00	1.00	3.00	-	0 (0)
7. Shoulder injuries		0.00	0.00	0.00	0.00	2.00	-	0 (0)
8. lumbar spine injuries		0.00	0.00	0.00	0.00	3.00	-	0 (0)
9. iliopsoas IMIs		0.00	0.00	0.00	0.00	2.00	-	0 (0)
10. hip adductor IMIs		0.00	0.00	0.00	0.00	3.00	-	0 (0)
11.hamstring IMIs		0.00	0.00	0.00	1.00	5.00	-	0 (0)
12. quadriceps IMIs		0.00	0.00	0.00	0.00	3.00	-	0 (0)
13. calf IMIs		0.00	0.00	0.00	0.00	4.00	-	0 (0)
**Within 3 years prior to PHE, most recent:**								
14. foot/ankle injury	*Never*	-	-	-	-	-	120 (46.15)	0 (0)
	< 6 months	-	-	-	-	-	37 (14.23)	0 (0)
	6-12 months	-	-	-	-	-	29 (11.15)	0 (0)
	>12 months	-	-	-	-	-	74 (28.46)	0 (0)
15. Hip/groin injury	*Never*	-	-	-	-	-	176 (67.69)	0 (0)
	< 6 months	-	-	-	-	-	21 (8.08)	0 (0)
	6-12 months	-	-	-	-	-	20 (7.69)	0 (0)
	>12 months	-	-	-	-	-	43 (16.54)	0 (0)
16. Knee injury	*Never*	-	-	-	-	-	165 (63.46)	0 (0)
	< 6 months	-	-	-	-	-	15 (5.77)	0 (0)
	6-12 months	-	-	-	-	-	24 (9.23)	0 (0)
	>12 months	-	-	-	-	-	56 (21.54)	0 (0)
17. Shoulder injury	*Never*	-	-	-	-	-	240 (92.31)	0 (0)
	< 6 months	-	-	-	-	-	6 (2.31)	0 (0)
	6-12 months	-	-	-	-	-	4 (1.54)	0 (0)
	>12 months	-	-	-	-	-	10 (3.85)	0 (0)
18. lumbar spine injury	*Never*	-	-	-	-	-	210 (80.77)	0 (0)
	< 6 months	-	-	-	-	-	8 (3.08)	0 (0)
	6-12 months	-	-	-	-	-	7 (2.69)	0 (0)
	>12 months	-	-	-	-	-	35 (13.46)	0 (0)
19. iliopsoas IMI	*Never*	-	-	-	-	-	237 (91.15)	0 (0)
	< 6 months	-	-	-	-	-	2 (0.77)	0 (0)
	6-12 months	-	-	-	-	-	7 (2.69)	0 (0)
	>12 months	-	-	-	-	-	14 (5.38)	0 (0)
20. hip adductor IMI	*Never*	-	-	-	-	-	214 (82.31)	0 (0)
	< 6 months	-	-	-	-	-	17 (6.54)	0 (0)
	6-12 months	-	-	-	-	-	10 (3.85)	0 (0)
	>12 months	-	-	-	-	-	19 (7.31)	0 (0)
21.hamstring muscle IMI	*Never*	-	-	-	-	-	193 (74.23)	0 (0)
	< 6 months	-	-	-	-	-	8 (3.08)	0 (0)
	6-12 months	-	-	-	-	-	23 (8.85)	0 (0)
	>12 months	-	-	-	-	-	36 (13.85)	0 (0)
22. quadriceps IMI	*Never*	-	-	-	-	-	165 (63.46)	0 (0)
	< 6 months	-	-	-	-	-	5 (1.92)	0 (0)
	6-12 months	-	-	-	-	-	12 (4.62)	0 (0)
	>12 months	-	-	-	-	-	24 (9.23)	0 (0)
23. calf IMI	*Never*	-	-	-	-	-	230 (88.46)	0 (0)
	< 6 months	-	-	-	-	-	10 (3.85)	0 (0)
	6-12 months	-	-	-	-	-	5 (1.92)	0 (0)
	>12 months	-	-	-	-	-	15 (5.77)	0 (0)
**Musculoskeletal Examination**								
24. Mean PROM hip IR (deg.)		9.50	22.00	32.50	40.00	55.00	-	15 (5.77)
25. Mean PROM hip ER (deg.)		17.50	33.70	39.00	43.50	62.00	-	15 (5.77)
26. Mean hip flexor length (deg.)		-7.00	3.00	8.68	15.00	55.00	-	18 (6.92)
27. Mean hamstring/neural mobility length (deg.)		45.00	83.00	90.00	90.00	102.00	-	15 (5.77)
28. Mean calf muscle length (deg.)		9.50	25.65	30.50	37.00	57.50	-	15 (5.77)
**Strength/Power Tests**								
29: Max. leg extension power difference ($W/kg^{-0.67}$)		-11.94	-1.47	0.43	1.91	15.26	-	39 (15.00)
30: Mean of max. leg extension power ($W/kg^{-0.67}$)		23.01	41.67	46.40	51.10	78.70	-	39 (15.00)
31: Max. leg extension velocity difference ($m.s^{-1}$)		-0.34	-0.05	0.02	0.11	0.40	-	35 (13.46)
32: Mean of max. leg extension velocity ($m.s^{-1}$)		1.02	1.68	1.84	1.98	2.35	-	35 (13.46)
33: Max leg extension force difference ($N/kg^{-0.67}$)		-83.59	-5.74	-1.07	4.39	55.41	-	39 (15.00)
34: Mean of max. leg extension force ($N/kg^{-0.67}$)		50.19	90.85	101.14	112.02	217.96	-	39 (15.00)

For peer review only

35: CMJ Force per kg of body mass	20.50	23.30	25.18	27.90	39.20	-	35 (13.46)
36: CMJ height (cm)	28.70	37.20	39.90	43.10	58.00	-	35 (13.46)

Key: PHE= periodic health examination; I-IMI=index indirect muscle injury; IMI= indirect muscle injury; min = minimum; max = maximum; n = observations; Freq= frequency; WBL=weight bearing lunge; CMJ=countermovement jump; PROM=passive range of movement; deg. = degrees; SLR= straight leg raise; BMI= body mass index; kg/m^2 = kilograms/body height (metres) squared; *cm = centimetres; Kg=kilograms; Cont.=continuous; dis./cont.= discrete treated as continuous; cat.= categorical. Note that for the Musculoskeletal Examination and Strength/Power factors, positive values indicate greater left limb values compared to right limb values; negative values indicate greater right limb*

For peer review only

Does preseason screening provide a source of potential prognostic factors for indirect muscle injuries in elite football (soccer) players? An exploratory analysis using routinely-collected periodic health examination data

Hughes, T., Riley, R.D., Callaghan, M.J. and Sergeant, J.C. (2022)

Supplementary file 5: Graph to show the distribution of observed and imputed values for continuous variables

For peer review only

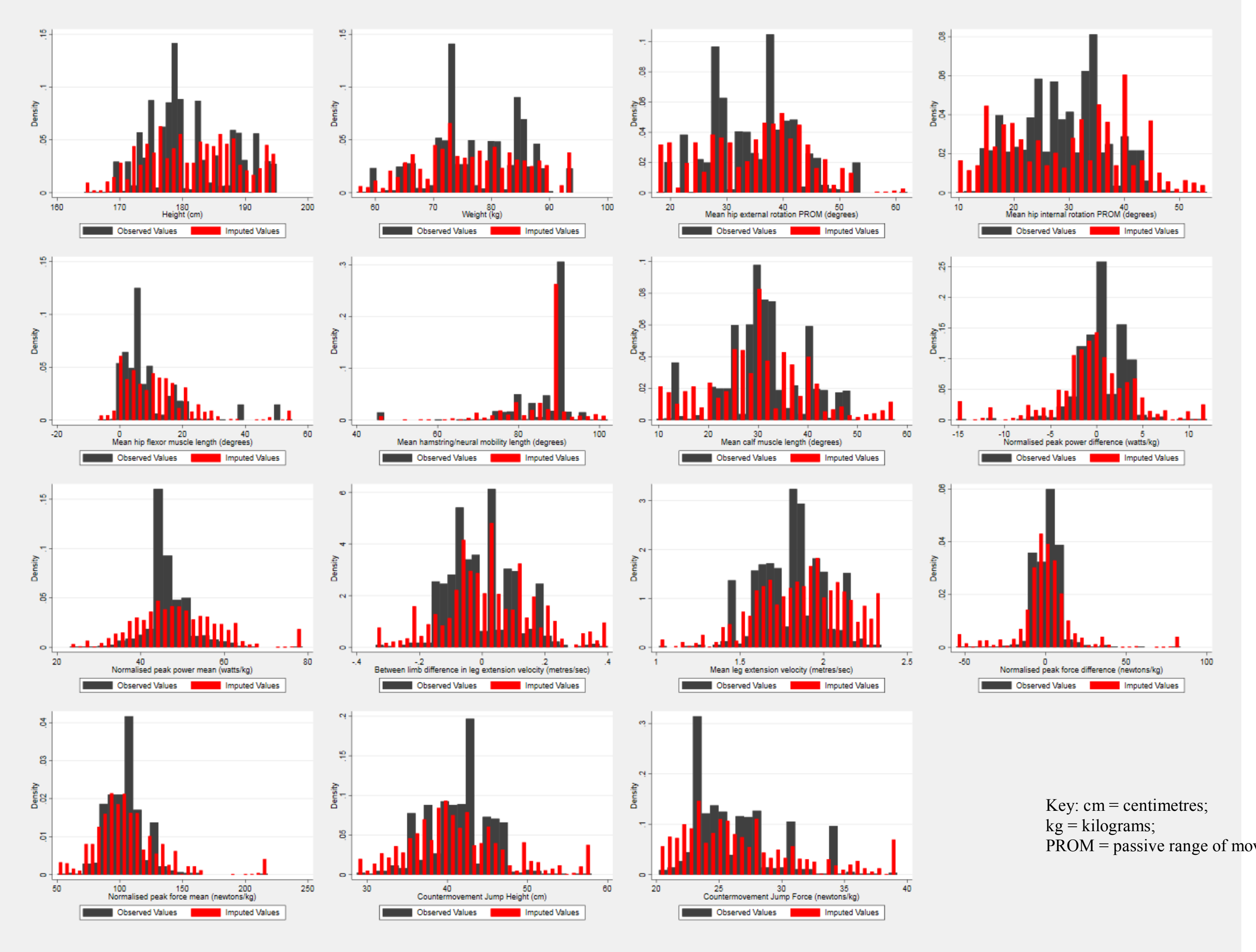

Key: cm = centimetres;
kg = kilograms;
PROM = passive range of movement

Does preseason screening provide a source of potential prognostic factors for indirect muscle injuries in elite football (soccer) players? An exploratory analysis using routinely-collected periodic health examination data

Hughes, T., Riley, R.D., Callaghan, M.J. and Sergeant, J.C. (2022)

Supplementary file 6: Results of univariable and multivariable analyses – Primary complete case analysis

For peer review only

	Univariable (unadjusted)				Multivariable (adjusted for age, height, weight)			
**Candidate PF & Type**	OR	95% CI	P	Best model fit	OR	95% CI	P	Best model fit
**Anthropometric (Adjustment Factors):**								
1: Age (years)	**1.12**	**1.06 to 1.18**	**<0.001**	**Linear**	**1.11**	**1.04 to 1.18**	**<0.001**	**Linear**
2: Height (cm)	1.03	0.99 to 1.06	0.18	-	1.02	0.97 to 1.07	0.50	-
3: Weight (kg)	1.03	0.99 to 1.06	0.07	-	0.99	0.95 to 1.04	0.81	-
**Within 3 years preceding PHE, frequency of:**								
4: foot/ankle injuries	1.04	0.87 to 1.23	0.68	-	1.04	0.86 to 1.25	0.70	-
5: hip/groin injuries	1.16	0.90 to 1.51	0.25	-	1.29	0.98 to 1.70	0.07	-
6: knee injuries	0.96	0.72 to 1.29	0.81	-	1.00	0.74 to 1.35	0.98	-
7: shoulder injuries	2.38	0.98 to 5.75	0.05	-	1.77	0.68 to 4.59	0.24	-
8: lumbar spine injuries	0.88	0.60 to 1.29	0.50	-	1.03	0.68 to 1.55	0.89	-
9: iliopsoas IMIs	0.73	0.38 to 1.43	0.37	-	0.88	0.44 to 1.76	0.72	-
10: hip adductor IMIs	1.38	0.92 to 2.09	0.12	-	1.22	0.78 to 1.90	0.39	-
11: hamstring IMIs	**1.56**	**1.17 to 2.09**	**<0.001**	**Linear**	1.30	0.96 to 1.77	0.09	-
12: quadriceps IMIs	1.08	0.67 to 1.73	0.75	-	1.00	0.61 to 1.63	0.99	-
13: calf IMIs	**1.80**	**1.09 to 2.97**	**0.02**	**Linear**	1.30	0.74 to 2.29	0.35	-
**Within 3 years preceding PHE, most recent:**								
14: foot/ankle injury (never)	ref	ref	ref	-	ref	ref	ref	-
14: foot/ankle injury (<6 months)	1.27	0.64 to 2.53	0.49	-	1.36	0.66 to 2.79	0.40	-
14: foot/ankle injury (6-12 months)	1.16	0.54 to 2.46	0.71	-	1.09	0.49 to 2.45	0.83	-
14: foot/ankle injury (>12 months)	1.27	0.75 to 2.13	0.37	-	1.05	0.60 to 1.84	0.86	-
15: hip/groin injury (never)	ref	ref	ref	-	ref	ref	ref	-
15: hip/groin injury (<6 months)	1.05	0.44 to 2.49	0.92	-	1.45	0.52 to 4.00	0.48	-
15: hip/groin injury (6-12 months)	0.59	0.23 to 1.50	0.27	-	1.17	0.35 to 3.84	0.80	-
15: hip/groin injury (>12months)	1.58	0.86 to 2.87	0.14	-	1.14	0.45 to 2.88	0.78	-
16: knee injury (never)	ref	ref	ref	-	ref	ref	ref	-
16: knee injury (<6 months)	1.15	0.40 to 3.28	0.80	-	1.11	0.33 to 3.72	0.87	-
16: knee injury (6-12 months)	1.23	0.58 to 2.62	0.60	-	1.24	0.57 to 2.72	0.59	-
16: knee injury (>12months)	0.93	0.53 to 1.60	0.79	-	1.10	0.61 to 1.98	0.76	-
17: shoulder injury (never)	ref	ref	ref	-	ref	ref	ref	-
17: shoulder injury (<6 months)	2.75	0.50 to 15.26	0.25	-	2.07	0.33 to 12.90	0.44	-
17: shoulder injury (6-12 months)	1.38	0.19 to 9.90	0.75	-	1.12	0.15 to 8.37	0.91	-
17: shoulder injury (>12months)	3.21	0.81 to 12.66	0.10	-	2.38	0.59 to 9.70	0.23	-
18: lumbar spine injury (never)	ref	ref	ref	-	ref	ref	ref	-
18: lumbar spine injury (<6 months)	1.24	0.30 to 5.05	0.77	-	2.66	0.57 to 12.51	0.22	-
18: lumbar spine injury (6-12 months)	0.62	0.15 to 2.52	0.50	-	0.72	0.17 to 3.03	0.66	-
18: lumbar spine injury (>12months)	0.70	0.34 to 1.44	0.33	-	0.90	0.42 to 1.94	0.79	-
19: iliopsoas IMI (never)	ref	ref	ref	-	ref	ref	ref	-
19: iliopsoas IMI (<6 months)	1.24	0.08 to 20.05	0.88	-	1.29	0.08 to 21.58	0.86	-
19: iliopsoas IMI (6-12 months)	0.62	0.15 to 2.53	0.51	-	0.80	0.19 to 3.33	0.76	-
19: iliopsoas IMI (>12months)	0.57	0.21 to 1.60	0.27	-	0.70	0.25 to 1.96	0.50	-
20: hip adductor IMI (never)	ref	ref	ref	-	ref	ref	ref	-
20: hip adductor IMI (<6 months)	1.37	0.53 to 3.56	0.52	-	1.45	0.52 to 4.00	0.48	-
20: hip adductor IMI (6-12 months)	1.37	0.43 to 4.36	0.60	-	1.17	0.35 to 3.84	0.80	-
20: hip adductor IMI (>12months)	1.37	0.59 to 3.16	0.46	-	1.14	0.45 to 2.88	0.78	-
21: hamstring IMI (never)	ref	ref	ref	-	ref	ref	ref	-
21: hamstring IMI (<6 months)	2.84	0.81 to 9.99	0.10	-	1.44	0.36 to 5.77	0.60	-
21: hamstring IMI (6-12 months)	1.42	0.66 to 3.06	0.37	-	1.25	0.55 to 2.86	0.60	-
21: hamstring IMI (>12months)	**2.95**	**1.51 to 5.73**	**<0.001**	**Linear**	2.10	1.01 to 4.34	0.05	-
22: quadriceps IMI (never)	ref	ref	ref	-	ref	ref	ref	-
22: quadriceps IMI (<6 months)	1.74	0.38 to 7.91	0.48	-	1.52	0.32 to 7.20	0.60	-
22: quadriceps IMI (6-12 months)	0.58	0.17 to 1.93	0.37	-	0.72	0.21 to 2.50	0.60	-
22: quadriceps IMI (>12months)	1.14	0.53 to 2.42	0.74	-	0.85	0.37 to 1.94	0.69	-
23: calf IMI (never)	ref	ref	ref	-	ref	ref	ref	-
23: calf IMI (<6 months)	3.78	0.98 to 14.56	0.05	-	2.80	0.68 to 11.47	0.15	-
23: calf IMI (6-12 months)	7.09	0.82 to 61.51	0.08	-	3.36	0.35 to 32.71	0.30	-
23: calf IMI (>12months)	1.26	0.56 to 0.89	0.64	-	0.87	0.29 to 2.62	0.80	-
**Musculoskeletal:**								
24. Mean PROM hip IR (deg.)	**0.97**	**0.95 to 0.99**	**0.01**	**Linear**	**0.97**	**0.95 to 1.00**	**0.04**	**Linear**
25. Mean PROM hip ER (deg.)	0.97	0.94 to 1.00	0.08	-	0.98	0.95 to 1.01	0.26	-
26. Mean hip flexor length (deg.)	1.01	0.98 to 1.04	0.50	-	1.01	0.98 to 1.04	0.50	-

For peer review only

27. Mean hamstring/neural mobility length (deg.)	0.99	0.96 to 1.02	0.53	-	0.98	0.94 to 1.01	0.21	-
28. Mean calf muscle length (deg.)	1.00	0.97 to 1.02	0.73	-	0.99	0.97 to 1.02	0.64	-
**Strength/Power:**								
29: Max. leg extension power difference ($W/kg^{-0.67}$)	1.00	0.93 to 1.07	0.92	-	0.99	0.92 to 1.07	0.78	-
30: Mean of max. leg extension power ($W/kg^{-0.67}$)	1.02	0.99 to 1.05	0.30	-	1.01	0.97 to 1.04	0.76	-
31: Max. leg extension velocity difference ($m.s^{-1}$)	2.14	0.30 to 15.00	0.45	-	2.61	0.34 to 19.87	0.36	-
32: Mean of max. leg extension velocity ($m.s^{-1}$)	1.86	0.63 to 5.53	0.26	-	1.44	0.46 to 4.53	0.53	-
33: Max leg extension force difference ($N/kg^{-0.67}$)	1.00	0.98 to 1.02	0.66	-	1.00	0.98 to 1.02	0.67	-
34: Mean of max. leg extension force ($N/kg^{-0.67}$)	1.00	0.99 to 1.01	0.96	-	1.00	0.98 to 1.01	0.69	-
35: CMJ Force per kg of body mass (N/kg)	0.99	0.92 to 1.06	0.73	-	0.99	0.92 to 1.06	0.82	-
36: CMJ height (cm)	1.03	0.97 to 1.07	0.34	-	1.01	0.96 to 1.07	0.59	-

Key: PHE= periodic health examination; PF= prognostic factor; OR=odds ratio; CI=confidence interval; ref=reference category; I-IMI=index indirect muscle injury; IMI= indirect muscle injury; Freq= frequency; WBL=weight bearing lunge; CMJ=countermovement jump; PROM=passive range of movement; deg. = degrees; SLR= straight leg raise; BMI= body mass index; kg/m^2 = kilograms/body height (metres) squared; *cm = centimetres; Kg=kilograms; W= watts (note:* $W/kg^{-0.67}$ *has a scaling factor to normalise force to body mass[42]); N= newtons (note:* $N/kg^{-0.67}$ *has a scaling factor to normalise force to body mass);max.=maximum; m.s.= metres per second; cm = centimetres; Kg=kilograms; - = not applicable.*
Note: *ORs are expressed per one-unit increase for all continuous factors, and according to category for all categorical factors; Factors in* **bold** *indicate significance at the 0.05 level.*

For peer review only

Does preseason screening provide a source of potential prognostic factors for indirect muscle injuries in elite football (soccer) players? An exploratory analysis using routinely-collected periodic health examination data

Hughes, T., Riley, R.D., Callaghan, M.J. and Sergeant, J.C. (2022)

Supplementary file 7: Results of univariable and multivariable analyses – Sensitivity analysis using imputed data

For peer review only

	Univariable (unadjusted)				Multivariable (adjusted for age, height, weight)			
**Candidate PF & Type**	OR	95% CI	P	Best model fit	OR	95% CI	P	Best model fit
**Anthropometric (Adjustment Factors):**								
1: Age (years)	**1.15**	1.08 to 1.22	**<0.001**	**Linear**	**1.14**	**1.06 to 1.22**	**<0.001**	**Linear**
2: Height (cm)	1.02	0.98 to 1.06	0.25	-	0.99	0.94 to 1.05	0.85	-
3: Weight (kg)	**1.05**	**1.02 to 1.08**	**0.01**	**Linear**	1.02	0.97 to 1.07	0.49	-
**Within 3 years preceding PHE, frequency of:**								
4: foot/ankle injuries	1.03	0.86 to 1.25	0.73	-	1.04	0.85 to 1.27	0.70	-
5: hip/groin injuries	1.09	0.83 to 1.43	0.54	-	1.23	0.92 to 1.64	0.16	-
6: knee injuries	0.91	0.67 to 1.23	0.54	-	0.92	0.67 to 1.26	0.59	-
7: shoulder injuries	1.91	0.80 to 4.59	0.15	-	1.34	0.53 to 3.39	0.54	-
8: lumbar spine injuries	0.78	0.53 to 1.16	0.22	-	0.99	0.66 to1.50	0.98	-
9: iliopsoas IMIs	0.74	0.36 to 1.50	0.40	-	0.86	0.41 to 1.81	0.70	-
10: hip adductor IMIs	1.46	0.93 to 2.30	0.10	-	1.14	0.70 to 1.87	0.60	-
11: hamstring IMIs	**2.39**	**1.55 to 3.69**	**<0.001**	**Linear**	**1.87**	**1.19 to 2.96**	**0.01**	**Linear**
12: quadriceps IMIs	1.12	0.67 to 1.86	0.66	-	1.09	0.64 to 1.87	0.75	-
13: calf IMIs	**2.17**	**1.13 to 4.16**	**0.02**	**Linear**	1.47	0.70 to 3.08	0.31	-
**Within 3 years preceding PHE, most recent:**								
14: foot/ankle injury (never)	ref	ref	ref	-	ref	ref	ref	-
14: foot/ankle injury (<6 months)	1.25	0.59 to 2.61	0.56	-	1.52	0.70 to 3.31	0.29	-
14: foot/ankle injury (6-12 months)	1.27	0.57 to 2.85	0.57	-	1.30	0.55 to 3.09	0.55	-
14: foot/ankle injury (>12 months)	1.39	0.78 to 2.49	0.27	-	1.26	0.67 to 2.34	0.47	-
15: hip/groin injury (never)	ref	ref	ref	-	ref	ref	ref	-
15: hip/groin injury (<6 months)	0.77	0.31 to 1.91	0.57	-	1.36	0.52 to 3.52	0.53	-
15: hip/groin injury (6-12 months)	0.55	0.21 to 1.45	0.23	-	0.57	0.20 to 1.58	0.28	-
15: hip/groin injury (>12months)	1.56	0.79 to 3.09	0.20	-	1.87	0.92 to 3.81	0.09	-
16: knee injury (never)	ref	ref	ref	-	ref	ref	ref	-
16: knee injury (<6 months)	0.89	0.31 to 2.55	0.82	-	0.87	0.28 to 2.67	0.81	-
16: knee injury (6-12 months)	1.20	0.51 to 2.82	0.68	-	1.15	0.47 to 2.83	0.76	-
16: knee injury (>12months)	0.94	0.51 to 1.72	0.85	-	1.01	0.54 to 1.91	0.97	-
17: shoulder injury (never)	ref	ref	ref	-	ref	ref	ref	-
17: shoulder injury (<6 months)	2.14	0.38 to 11.89	0.39	-	1.83	0.31 to 11.00	0.51	-
17: shoulder injury (6-12 months)	1.07	0.15 to 7.71	0.95	-	0.80	0.10 to 6.32	0.83	-
17: shoulder injury (>12months)	2.49	0.63 to 9.87	0.19	-	1.58	0.37 to 6.62	0.53	-
18: lumbar spine injury (never)	ref	ref	ref	-	ref	ref	ref	-
18: lumbar spine injury (<6 months)	0.93	0.23 to 3.80	0.92	-	1.70	0.40 to 7.18	0.47	-
18: lumbar spine injury (6-12 months)	0.69	0.15 to 3.18	0.64	-	0.86	0.18 to 4.18	0.85	-
18: lumbar spine injury (>12months)	0.55	0.26 to 1.14	0.11	-	0.80	0.37 to 1.73	0.57	-
19: iliopsoas IMI (never)	ref	ref	ref	-	ref	ref	ref	-
19: iliopsoas IMI (<6 months)	0.98	0.06 to 15.77	0.99	-	1.01	0.06 to 17.67	0.99	-
19: iliopsoas IMI (6-12 months)	0.39	0.07 to 2.05	0.27	-	0.54	0.10 to 2.92	0.48	-
19: iliopsoas IMI (>12months)	0.73	0.25 to 2.17	0.57	-	0.81	0.26 to 2.50	0.71	-
20: hip adductor IMI (never)	ref	ref	ref	-	ref	ref	ref	-
20: hip adductor IMI (<6 months)	1.24	0.46 to 3.32	0.68	-	1.00	0.35 to 2.83	0.99	-
20: hip adductor IMI (6-12 months)	1.65	0.45 to 6.00	0.45	-	1.24	0.32 to 4.84	0.75	-
20: hip adductor IMI (>12months)	1.88	0.71 to 4.96	0.20	-	1.36	0.47 to 3.90	0.57	-
21: hamstring IMI (never)	ref	ref	ref	-	ref	ref	ref	-
21: hamstring IMI (<6 months)	4.06	0.80 to 20.64	0.09	-	2.64	0.49 to 14.29	0.26	-
21: hamstring IMI (6-12 months)	1.76	0.74 to 4.21	0.20	-	1.42	0.58 to 3.52	0.44	-
21: hamstring IMI (>12months)	**4.74**	**2.05 to 10.93**	**<0.001**	**Linear**	**3.03**	**1.25 to 7.36**	**0.01**	**Linear**
22: quadriceps IMI (never)	**ref**	ref	**ref**	-	**ref**	**ref**	**ref**	**-**
22: quadriceps IMI (<6 months)	4.11	0.45 to 37.37	0.21	-	4.09	0.42 to 39.53	0.22	-
22: quadriceps IMI (6-12 months)	0.51	0.15 to 1.76	0.29	-	0.51	0.14 to 1.85	0.31	-
22: quadriceps IMI (>12months)	1.21	0.52 to 2.83	0.65	-	1.13	0.47 to 2.77	0.77	-
23: calf IMI (never)	ref	ref	ref	-	ref	ref	ref	-
23: calf IMI (<6 months)	4.44	0.92 to 21.36	0.06	-	3.62	0.71 to 18.47	0.12	-
23: calf IMI (>6 months)*	1.66	0.66 to 4.23	0.28	-	0.79	0.27 to 2.33	0.67	-
**Musculoskeletal:**								
24. Mean PROM hip IR (deg.)	**0.97**	**0.94 to 0.99**	**0.01**	**Linear**	0.98	0.95 to 1.01	0.12	-
25. Mean PROM hip ER (deg.)	**0.96**	**0.93 to 1.00**	**0.03**	**Linear**	0.99	0.95 to 1.02	0.46	-
26. Mean hip flexor length (deg.)	1.02	0.99 to 1.05	0.15	-	1.03	0.99 to 1.07	0.07	-
27. Mean hamstring/neural mobility length (deg.)	0.99	0.95 to 1.02	0.44	-	0.97	0.93 to 1.01	0.15	-
28. Mean calf muscle length (deg.)	0.99	0.96 to 1.02	0.45	-	0.99	0.96 to 1.02	0.34	-

For peer review only

**Strength/Power:**								
29: Max. leg extension power difference (W/kg$^{-0.67}$)	0.98	0.91 to 1.06	0.58	-	0.97	0.90 to 1.06	0.48	-
30: Mean of max. leg extension power (W/kg$^{-0.67}$)	1.03	0.99 to 1.06	0.14	-	1.00	0.97 to 1.04	0.77	-
31: Max. leg extension velocity difference (m.s^{-1})	2.28	0.28 to 18.29	0.44	-	3.24	0.37 to 28.60	0.29	-
32: Mean of max. leg extension velocity (m.s^{-1})	2.44	0.77 to 7.72	0.13	-	1.65	0.46 to 5.80	0.44	-
33: Max leg extension force difference (N/kg$^{-0.67}$)	0.99	0.97 to 1.01	0.50	-	0.99	0.98 to 1.01	0.44	-
34: Mean of max. leg extension force (N/kg$^{-0.67}$)	1.00	0.99 to 1.01	0.94	-	0.99	0.98 to 1.02	0.63	-
35: CMJ Force per kg of body mass (N/kg)	0.99	0.92 to 1.06	0.80	-	0.98	0.90 to 1.06	0.60	-
36: CMJ height (cm)	1.03	0.98 to 1.09	0.25	-	1.00	0.95 to 1.07	0.85	-

Key: PHE= periodic health examination; PF= prognostic factor; OR=odds ratio; CI=confidence interval; ref=reference category; I-IMI=index indirect muscle injury; IMI= indirect muscle injury; Freq= frequency; WBL=weight bearing lunge; CMJ=countermovement jump; PROM=passive range of movement; deg. = degrees; SLR= straight leg raise; BMI= body mass index; kg/m^2 = kilograms/body height (metres) squared; *cm = centimetres; Kg=kilograms; W= watts (note: W/kg$^{-0.67}$ has a scaling factor to normalise force to body mass[42]); N= newtons (note: N/kg$^{-0.67}$ has a scaling factor to normalise force to body mass);max.=maximum; m.s.= metres per second; cm = centimetres; Kg=kilograms;* - *= not applicable; * indicates merged category (using calf IMI (6-12 months) and calf IMI (>12months). This was required due to the occurrence of perfect predictions in the Calf (6-12 months) category as a consequence of reduced sample size.* ***Note:*** *ORs are expressed per one-unit increase for all continuous factors, and according to category for all categorical factors; Factors in* **bold** *indicate significance at the 0.05 level.*

For peer review only

Does preseason screening provide a source of potential prognostic factors for indirect muscle injuries in elite football (soccer) players? An exploratory analysis using routinely-collected periodic health examination data

Hughes, T., Riley, R.D., Callaghan, M.J. and Sergeant, J.C. (2022)

Supplementary file 8: Results of univariable and multivariable analyses – Sensitivity analysis using complete case data

For peer review only

	Univariable (unadjusted)				Multivariable (adjusted for age, height, weight)			
**Candidate PF & Type**	OR	95% CI	P	Best model fit	OR	95% CI	P	Best model fit
**Anthropometric (Adjustment Factors):**								
1: Age (years)	**1.15**	1.08 to 1.22	**<0.00**	**Linear**	**1.14**	**1.64 to 1.22**	**<0.00**	**Linear**
2: Height (cm)	1.02	0.98 to 1.05	0.36	-	0.99	0.94 to 1.05	0.73	-
3: Weight (kg)	**1.04**	**1.01 to 1.08**	**0.01**	**Linear**	1.01	0.96 to 1.06	0.60	-
**Within 3 years preceding PHE, frequency of:**								
4: foot/ankle injuries	1.03	0.86 to 1.25	0.73	-	1.05	0.85 to 1.30	0.63	-
5: hip/groin injuries	1.09	0.83 to 1.43	0.54	-	1.23	0.92 to 1.65	0.16	-
6: knee injuries	0.91	0.67 to 1.23	0.54	-	0.93	0.67 to 1.28	0.65	-
7: shoulder injuries	1.91	0.80 to 4.59	0.15	-	1.23	0.46 to 3.24	0.69	-
8: lumbar spine injuries	0.78	0.53 to 1.16	0.22	-	0.96	0.63 to 1.46	0.84	-
9: iliopsoas IMIs	0.74	0.36 to 1.50	0.40	-	0.86	0.41 to 1.79	0.68	-
10: hip adductor IMIs	1.46	0.93 to 2.30	0.10	-	1.18	0.71 to 1.95	0.53	-
11: hamstring IMIs	**2.39**	**1.60 to 3.69**	**<0.00**	**Linear**	**1.72**	**1.09 to 2.73**	**0.02**	**Linear**
12: quadriceps IMIs	1.12	0.67 to 1.86	0.66	-	1.05	0.61 to 1.82	0.90	-
13: calf IMIs	**2.17**	**1.13 to 4.16**	**0.02**	**Linear**	1.52	0.69 to 3.33	0.30	-
**Within 3 years preceding PHE, most recent:**								
14: foot/ankle injury (never)	ref	ref	ref	-	ref	ref	ref	-
14: foot/ankle injury (<6 months)	1.24	0.46 to 3.32	0.67	-	1.52	0.69 to 3.34	0.30	-
14: foot/ankle injury (6-12 months)	1.65	0.46 to 6.00	0.45	-	1.32	0.54 to 3.21	0.54	-
14: foot/ankle injury (>12 months)	1.90	0.71 to 4.97	0.20	-	1.12	0.58 to 2.14	0.74	-
15: hip/groin injury (never)	ref	ref	ref	-	ref	ref	ref	-
15: hip/groin injury (<6 months)	0.77	0.31 to 1.91	0.57	-	1.30	0.50 to 3.38	0.60	-
15: hip/groin injury (6-12 months)	0.55	0.31 to 1.91	0.23	-	0.72	0.25 to 2.05	0.54	-
15: hip/groin injury (>12months)	1.56	0.79 to 3.09	0.20	-	1.79	0.87 to 3.69	0.11	-
16: knee injury (never)	ref	ref	ref	-	ref	ref	ref	-
16: knee injury (<6 months)	0.88	0.31 to 2.55	0.82	-	0.92	0.27 to 3.12	0.89	-
16: knee injury (6-12 months)	1.20	0.51 to 2.82	0.68	-	1.14	0.46 to 2.82	0.77	-
16: knee injury (>12months)	0.94	0.51 to 1.72	0.85	-	1.10	0.57 to 2.12	0.77	-
17: shoulder injury (never)	ref	ref	ref	-	ref	ref	ref	-
17: shoulder injury (<6 months)	2.14	0.38 to 11.89	0.38	-	1.54	0.24 to 9.91	0.65	-
17: shoulder injury (6-12 months)	1.07	0.15 to 7.71	0.94	-	0.80	0.10 to 6.27	0.83	-
17: shoulder injury (>12months)	2.49	0.63 to 9.87	0.19	-	1.55	0.37 to 6.52	0.548	-
18: lumbar spine injury (never)	ref	ref	ref	-	ref	ref	ref	-
18: lumbar spine injury (<6 months)	0.93	0.23 to 3.80	0.92	-	2.20	0.46 to 10.40	0.32	-
18: lumbar spine injury (6-12 months)	0.69	0.15 to 3.18	0.91	-	0.84	0.17 to 4.06	0.83	-
18: lumbar spine injury (>12months)	0.55	0.26 to 1.14	0.11	-	0.75	0.34 to 1.66	0.48	-
19: iliopsoas IMI (never)	ref	ref	ref	-	ref	ref	ref	-
19: iliopsoas IMI (<6 months)	1.18	0.07 to 19.09	0.91	-	1.04	0.06 to 18.44	0.98	-
19: iliopsoas IMI (6-12 months)	0.47	0.09 to 2.48	0.38	-	0.53	0.10 to 2.85	0.46	-
19: iliopsoas IMI (>12months)	0.89	0.30 to 2.63	0.83	-	0.80	0.26 to 2.46	0.70	-
20: hip adductor IMI (never)	ref	ref	ref	-	ref	ref	Ref	-
20: hip adductor IMI (<6 months)	1.49	0.55 to 4.00	0.43	-	1.12	0.38 to 3.30	0.84	-
20: hip adductor IMI (6-12 months)	1.59	0.47 to 5.36	0.46	-	1.23	0.32 to 4.76	0.69	-
20: hip adductor IMI (>12months)	1.76	0.71 to 4.36	0.22	-	1.43	0.47 to 4.35	0.531	-
21: hamstring IMI (never)	ref	ref	ref	-	ref	ref	ref	-
21: hamstring IMI (<6 months)	4.06	0.80 to 20.63	0.09	-	1.64	0.27 to 9.99	0.59	-
21: hamstring IMI (6-12 months)	1.76	0.80 to 20.63	0.09	-	1.25	0.50 to 3.13	0.64	-
21: hamstring IMI (>12months)	**4.73**	**2.05 to 10.93**	**<0.00**	**Linear**	**2.94**	**1.15 to 7.53**	**0.03**	**Linear**
22: quadriceps IMI (never)	ref	ref	ref	-	ref	ref	ref	-
22: quadriceps IMI (<6 months)	4.11	0.45 to 37.37	0.21	-	4.03	0.41 to 39.22	0.23	-
22: quadriceps IMI (6-12 months)	0.51	0.15 to 1.76	0.29	-	0.66	0.18 to 2.39	0.52	-
22: quadriceps IMI (>12months)	1.21	0.52 to 2.83	0.65	-	0.89	0.35 to 2.28	0.81	-
23: calf IMI (never)	ref	ref	ref	-	ref	ref	ref	-
23: calf IMI (<6 months)	4.44	0.92 to 21.36	0.06	-	3.33	0.64 to 17.30	0.15	-
23: calf IMI (>6 months)*	1.66	0.66 to 4.23	0.28	-	0.88	0.29 to 2.76	0.84	-
**Musculoskeletal:**								
24. Mean PROM hip IR (deg.)	**0.97**	**0.94 to 0.99**	**0.01**	**Linear**	0.98	0.95 to 1.00	0.10	-
25. Mean PROM hip ER (deg.)	**0.96**	**0.93 to 0.99**	**0.02**	**Linear**	0.98	0.94 to 1.02	0.26	-
26. Mean hip flexor length (deg.)	1.02	0.99 to 1.05	0.21	-	1.03	0.99 to 1.06	0.13	-
27. Mean hamstring/neural mobility length (deg.)	0.98	0.95 to 1.02	0.40	-	0.96	0.92 to 1.00	0.06	-

For peer review only

28. Mean calf muscle length (deg.)	0.99	0.96 to 1.02	0.38	-	0.98	0.95 to 1.01	0.21	-
**Strength/Power:**								
29: Max. leg extension power difference ($W/kg^{-0.67}$)	0.98	0.90 to 1.06	0.63	-	0.97	0.89 to 1.06	0.59	-
30: Mean of max. leg extension power ($W/kg^{-0.67}$)	1.03	0.99 to 1.06	0.16	-	1.00	0.97 to 1.05	0.81	-
31: Max. leg extension velocity difference ($m.s^{-1}$)	2.64	0.31 to 22.58	0.38	-	3.87	0.39 to 38.79	0.25	-
32: Mean of max. leg extension velocity ($m.s^{-1}$)	2.49	0.74 to 8.32	0.14	-	1.60	0.42 to 6.09	0.49	-
33: Max leg extension force difference ($N/kg^{-0.67}$)	0.99	0.97 to 1.01	0.32	-	0.99	0.97 to 1.01	0.33	-
34: Mean of max. leg extension force ($N/kg^{-0.67}$)	1.00	0.99 to 1.01	0.98	-	1.00	0.98 to 1.01	0.59	-
35: CMJ Force per kg of body mass (N/kg)	0.98	0.91 to 1.05	0.58	-	0.97	0.89 to 1.05	0.47	-
36: CMJ height (cm)	1.03	0.97 to 1.09	0.35	-	1.00	0.95 to 1.07	0.86	-

Key: PHE= periodic health examination; PF= prognostic factor; OR=odds ratio; CI=confidence interval; ref=reference category; I-IMI=index indirect muscle injury; IMI= indirect muscle injury; Freq= frequency; WBL=weight bearing lunge; CMJ=countermovement jump; PROM=passive range of movement; deg. = degrees; SLR= straight leg raise; BMI= body mass index; kg/m^2 = kilograms/body height (metres) squared; *cm = centimetres; Kg=kilograms; W= watts (note: $W/kg^{-0.67}$ has a scaling factor to normalise force to body mass[42]); N= newtons (note: $N/kg^{-0.67}$ has a scaling factor to normalise force to body mass);max.=maximum; m.s.= metres per second; cm = centimetres; Kg=kilograms; - = not applicable; * indicates merged category (using calf IMI (6-12 months) and calf IMI (>12months). This was required due to the occurrence of perfect predictions in the Calf (6-12 months) category as a consequence of reduced sample size.* ***Note:*** *ORs are expressed per one-unit increase for all continuous factors, and according to category for all categorical factors; Factors in* ***bold*** *indicate significance at the 0.05 level.*

For peer review only

Does preseason screening provide a source of potential prognostic factors for indirect muscle injuries in elite football (soccer) players? An exploratory analysis using routinely-collected periodic health examination data

Hughes, T., Riley, R.D., Callaghan, M.J. and Sergeant, J.C. (2022)

Supplementary File 9: Comparison of candidate prognostic factors with statistically significant associations across the primary and sensitivity univariable analyses (using imputed and complete case datasets).

For peer review only

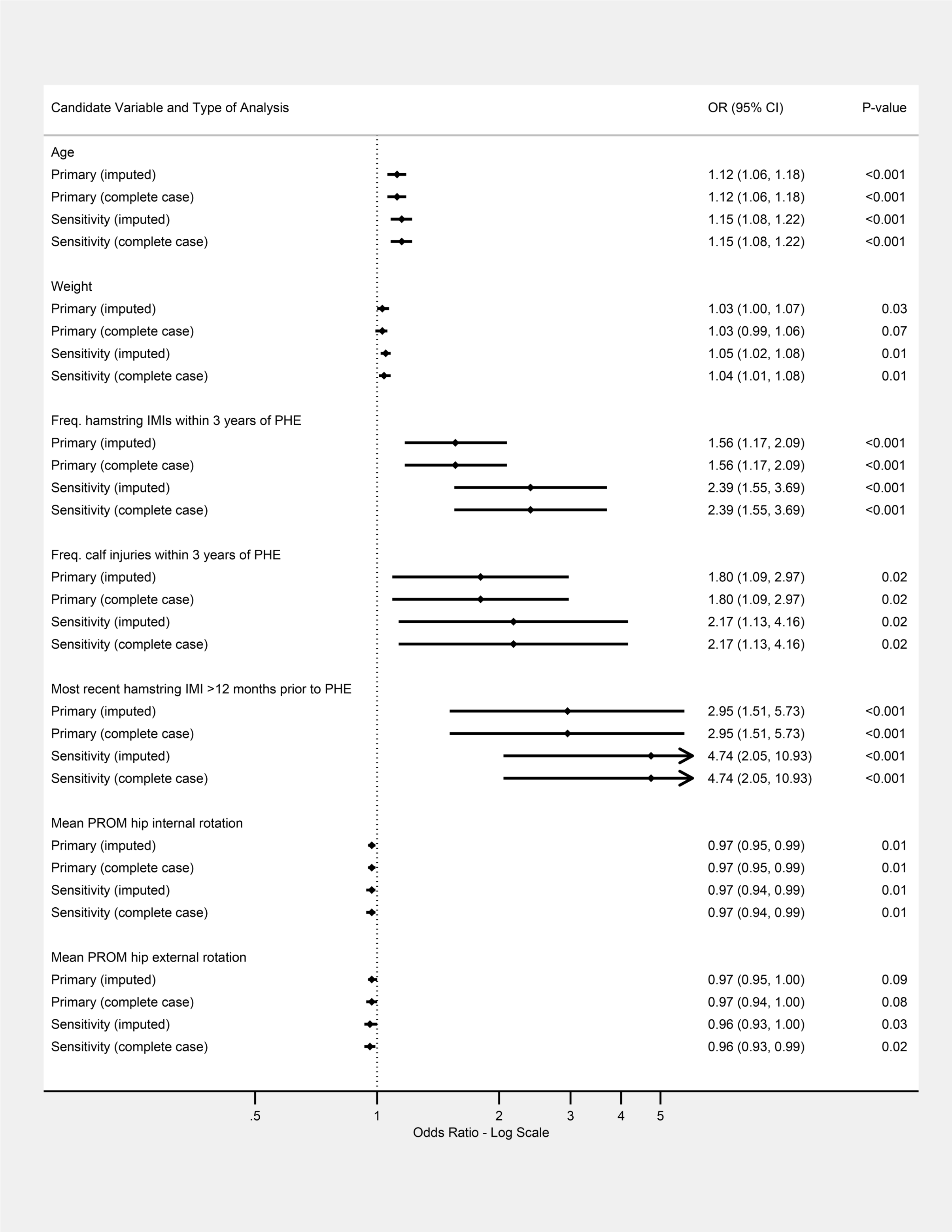

Key: OR = odds ratio; freq. = frequency; IMI = indirect muscle injury; PHE = periodic health examination; PROM = passive range of movement.

For peer review only

Does preseason screening provide a source of potential prognostic factors for indirect muscle injuries in elite football (soccer) players? An exploratory analysis using routinely-collected periodic health examination data

Hughes, T., Riley, R.D., Callaghan, M.J. and Sergeant, J.C. (2022)

Supplementary File 10: Comparison of candidate prognostic factors with statistically significant associations across the primary and sensitivity multivariable analyses (using imputed and complete case datasets).

For peer review only

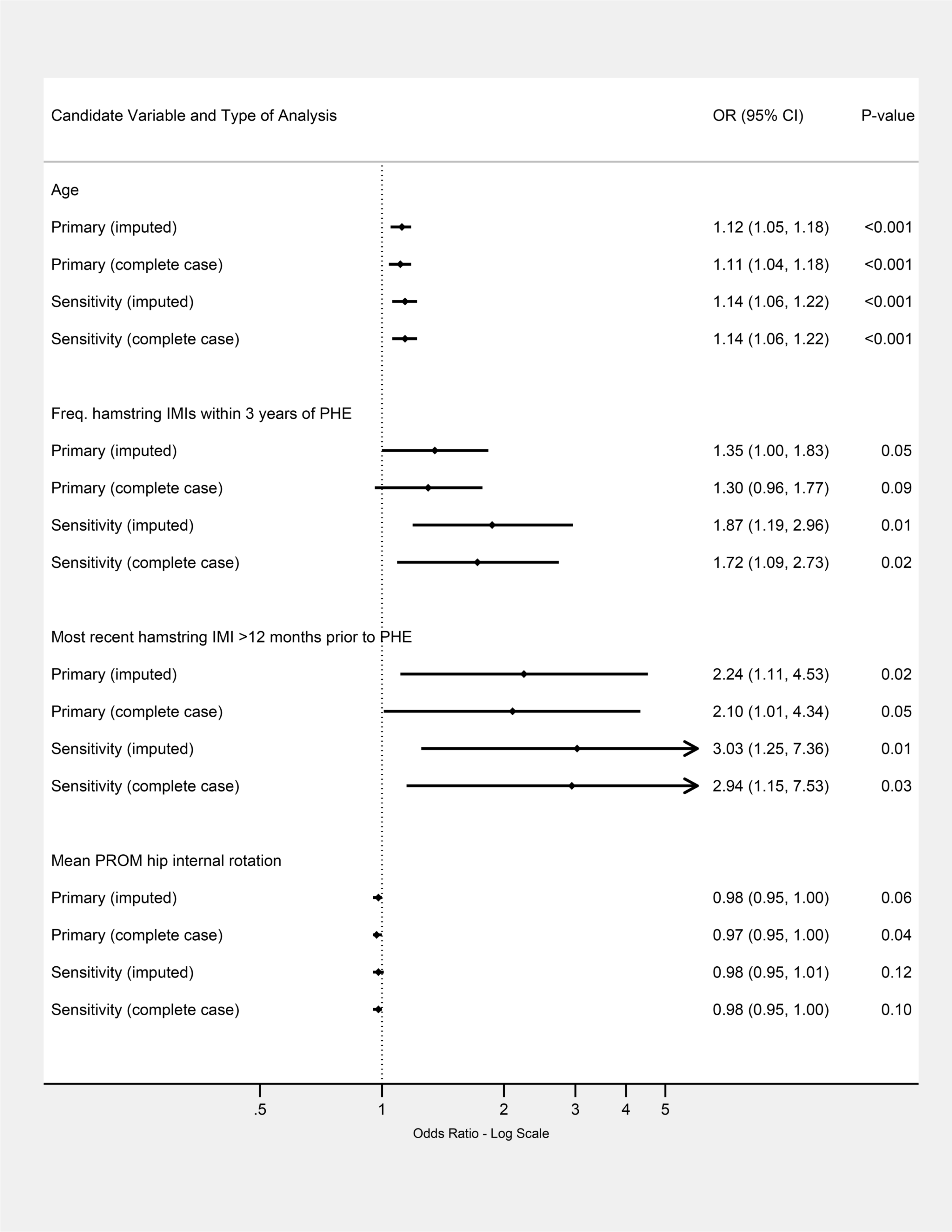

Key: IMI = indirect muscle injury; OR = odds ratio; freq. = frequency; PHE = periodic health examination; PROM = passive range of movement.

Note: the factor of age was adjusted for height and weight. All other factors were adjusted for age, height and weight.

The RECORD statement – checklist of items, extended from the STROBE statement, that should be reported in observational studies using routinely collected health data.

	Item No.	STROBE items	Location in manuscript where items are reported	RECORD items	Location in manuscript where items are reported
**Title and abstract**					
	1	(a) Indicate the study's design with a commonly used term in the title or the abstract (b) Provide in the abstract an informative and balanced summary of what was done and what was found	Page 2	RECORD 1.1: The type of data used should be specified in the title or abstract. When possible, the name of the databases used should be included.<br><br>RECORD 1.2: If applicable, the geographic region and timeframe within which the study took place should be reported in the title or abstract.<br><br>RECORD 1.3: If linkage between databases was conducted for the study, this should be clearly stated in the title or abstract.	Page 2<br><br>Page 2<br><br>n/a
**Introduction**					
Background rationale	2	Explain the scientific background and rationale for the investigation being reported	Pages 4-5		Pages 4-5
Objectives	3	State specific objectives, including any prespecified hypotheses	Page 5		Page 5
**Methods**					

For peer review only

Study Design	4	Present key elements of study design early in the paper	Page 5		Page 5
Setting	5	Describe the setting, locations, and relevant dates, including periods of recruitment, exposure, follow-up, and data collection	Page 6		Page 6

Participants	6	*(a) Cohort study*- Give the eligibility criteria, and the sources and methods of selection of participants. Describe methods of follow-up *Case-control study*- Give the eligibility criteria, and the sources and methods of case ascertainment and control selection. Give the rationale for the choice of cases and controls *Cross-sectional study*- Give the eligibility criteria, and the sources and methods of selection of participants<br><br>*(b) Cohort study*- For matched studies, give matching criteria and number of exposed and unexposed	Page 7<br><br>N/a<br><br>N/a	RECORD 6.1: The methods of study population selection (such as codes or algorithms used to identify subjects) should be listed in detail. If this is not possible, an explanation should be provided.<br><br>RECORD 6.2: Any validation studies of the codes or algorithms used to select the population should be referenced. If validation was conducted for this study and not published elsewhere, detailed methods and results should be provided.<br><br>RECORD 6.3: If the study involved linkage of databases, consider use of a flow diagram or other graphical display to demonstrate the data linkage process, including the number of individuals with linked data at each stage.	Page 7<br><br>N/a<br><br>N/a

For peer review only

		Case-control study- For matched studies, give matching criteria and the number of controls per case	N/a		
Variables	7	Clearly define all outcomes, exposures, predictors, potential confounders, and effect modifiers. Give diagnostic criteria, if applicable.	Pages 6-9	RECORD 7.1: A complete list of codes and algorithms used to classify exposures, outcomes, confounders, and effect modifiers should be provided. If these cannot be reported, an explanation should be provided.	Pages 6-9

For peer review only

Data sources/ measurement	8	For each variable of interest, give sources of data and details of methods of assessment (measurement). Describe comparability of assessment methods if there is more than one group	Page 6		Page 6

Bias	9	Describe any efforts to address potential sources of bias	Pages 6-9		Page 6-9
Study size	10	Explain how the study size was arrived at	Pages 7 -8		Pages 7-8
Quantitative variables	11	Explain how quantitative variables were handled in the analyses. If applicable, describe which groupings were chosen, and why	Pages 10-12		Pages 10-12

For peer review only

Statistical methods	12	(a) Describe all statistical methods, including those used to control for confounding<br>(b) Describe any methods used to examine subgroups and interactions<br>(c) Explain how missing data were addressed<br>(d) *Cohort study*- If applicable, explain how loss to follow-up was addressed<br>*Case-control study*- If applicable, explain how matching of cases and controls was addressed<br>*Cross-sectional study*- If applicable, describe analytical methods taking account of sampling strategy<br>(e) Describe any sensitivity analyses	Pages 10-12<br>Pages 10-12<br>Pages 10-12<br>Pages 10-12<br>Pages 10-12		Pages 10-12<br>Pages 10-12<br>Pages 10-12<br>Pages 10-12<br>Pages 10-12
Data access and cleaning methods		..		RECORD 12.1: Authors should describe the extent to which the investigators had access to the database population used to create the study population.	Pages 5-6

				RECORD 12.2: Authors should provide information on the data cleaning methods used in the study.	Page 6

For peer review only

Linkage		..	N/a	RECORD 12.3: State whether the study included person-level, institutional-level, or other data linkage across two or more databases. The methods of linkage and methods of linkage quality evaluation should be provided.	N/a
**Results**					
Participants	13	(a) Report the numbers of individuals at each stage of the study (*e.g.*, numbers potentially eligible, examined for eligibility, confirmed eligible, included in the study, completing follow-up, and analysed)<br>(b) Give reasons for non-participation at each stage.<br>(c) Consider use of a flow diagram		RECORD 13.1: Describe in detail the selection of the persons included in the study (*i.e.,* study population selection) including filtering based on data quality, data availability and linkage. The selection of included persons can be described in the text and/or by means of the study flow diagram.	Page 6
Descriptive data	14	(a) Give characteristics of study participants (*e.g.*, demographic, clinical, social) and information on exposures and potential confounders<br>(b) Indicate the number of participants with missing data for each variable of interest (c) *Cohort study*- summarise follow-up time (*e.g.*, average and total amount)	Pages 13-14<br><br>Figure 1		Pages 13-14<br><br>Figure 1

For peer review only

Outcome data	15	*Cohort study*- Report numbers of outcome events or summary measures over time<br>*Case-control study*- Report numbers in each exposure category, or summary measures of exposure<br>*Cross-sectional study*- Report numbers of outcome events or summary measures	Figure 1 and Pages 12-13		Figure 1 and Pages 12-13
Main results	16	(a) Give unadjusted estimates and, if applicable, confounder- adjusted estimates and their precision (e.g., 95% confidence interval). Make clear which confounders were adjusted for and why they were included<br>(b) Report category boundaries when continuous variables were categorized<br>(c) If relevant, consider translating estimates of relative risk into absolute risk for a meaningful time period	Page 17		Page 17
Other analyses	17	Report other analyses done—e.g., analyses of subgroups and interactions, and sensitivity analyses	Pages 18 and Supplementary files 5-9		Pages 18 and Supplementary files 5-9

For peer review only

**Discussion**					
Key results	18	Summarise key results with reference to study objectives	Pages 19-20		Pages 19-20
Limitations	19	Discuss limitations of the study, taking into account sources of potential bias or imprecision. Discuss both direction and magnitude of any potential bias	Page 22	RECORD 19.1: Discuss the implications of using data that were not created or collected to answer the specific research question(s). Include discussion of misclassification bias, unmeasured confounding, missing data, and changing eligibility over time, as they pertain to the study being reported.	Page 22
Interpretation	20	Give a cautious overall interpretation of results considering objectives, limitations, multiplicity of analyses, results from similar studies, and other relevant evidence	Page 23-25		Page 23-25
Generalisability	21	Discuss the generalisability (external validity) of the study results	Page 21		Page 21
**Other Information**					
Funding	22	Give the source of funding and the role of the funders for the present study and, if applicable, for the original study on which the present	Page 24		Page 24

For peer review only

		article is based			
Accessibility of protocol, raw data, and programming code		..	Page 24	RECORD 22.1: Authors should provide information on how to access any supplemental information such as the study protocol, raw data, or programming code.	Page 24

*Reference: Benchimol EI, Smeeth L, Guttmann A, Harron K, Moher D, Petersen I, Sørensen HT, von Elm E, Langan SM, the RECORD Working Committee. The REporting of studies Conducted using Observational Routinely-collected health Data (RECORD) Statement. *PLoS Medicine* 2015; in press.

*Checklist is protected under Creative Commons Attribution (CC BY) license.

For peer review only

The REMARK checklist

	Item to be reported	Page no.
**INTRODUCTION**		
1	State the marker examined, the study objectives, and any pre-specified hypotheses.	3-4
**MATERIALS AND METHODS**		
Patients		
2	Describe the characteristics (e.g., disease stage or co-morbidities) of the study patients, including their source and inclusion and exclusion criteria.	5-6
3	Describe treatments received and how chosen (e.g., randomized or rule-based).	n/a
Specimen/Prognostic Factor characteristics		
4	Describe type of biological material/prognostic factors used (including control samples) and methods of preservation and storage.	7-8
Assay methods/Prognostic factor measurement		
5	Specify the assay method used and provide (or reference) a detailed protocol, including specific reagents or kits used, quality control procedures, reproducibility assessments, quantitation methods, and scoring and reporting protocols. Specify whether and how assays were performed blinded to the study endpoint.	8
Study design		
6	State the method of case selection, including whether prospective or retrospective and whether stratification or matching (e.g., by stage of disease or age) was used. Specify the time period from which cases were taken, the end of the follow-up period, and the median follow-up time.	5
7	Precisely define all clinical endpoints examined.	5
8	List all candidate variables initially examined or considered for inclusion in models.	8
9	Give rationale for sample size; if the study was designed to detect a specified effect size, give the target power and effect size.	6
Statistical analysis methods		
10	Specify all statistical methods, including details of any variable selection procedures and other model-building issues, how model assumptions were verified, and how missing data were handled.	9-11
11	Clarify how marker values were handled in the analyses; if relevant, describe methods used for cutpoint determination.	9-11
**RESULTS**		
Data		
12	Describe the flow of patients through the study, including the number of patients included in each stage of the analysis (a diagram may be helpful) and reasons for dropout. Specifically, both overall and for each subgroup extensively examined report the numbers of patients and the number of events.	13
13	Report distributions of basic demographic characteristics (at least age and sex), standard (disease-specific) prognostic variables, and tumor marker, including numbers of missing values.	15
Analysis and presentation		
14	Show the relation of the marker to standard prognostic variables.	18
15	Present univariable analyses showing the relation between the marker and outcome, with the estimated effect (e.g., hazard ratio and survival probability). Preferably provide similar analyses for all other variables being analyzed. For the effect of a tumor marker on a time-to-event outcome, a Kaplan-Meier plot is recommended.	16-20
16	For key multivariable analyses, report estimated effects (e.g., hazard ratio) with confidence intervals for the marker and, at least for the final model, all other variables in the model.	17-21
17	Among reported results, provide estimated effects with confidence intervals from an analysis in which the marker and standard prognostic variables are included, regardless of their statistical significance.	16-21
18	If done, report results of further investigations, such as checking assumptions, sensitivity analyses, and internal validation.	19-22, Supp files 5-7)
**DISCUSSION**		
19	Interpret the results in the context of the pre-specified hypotheses and other relevant studies; include a discussion of limitations of the study.	22-26

Source: McShane LM, Altman DG, Sauerbrei W, Taube SE, Gion M, Clark GM: Reporting recommendations for tumor marker prognostic studies (REMARK). *J Natl Cancer Inst* 2005; 97: 1180-1184.

For peer review only - http://bmjopen.bmj.com/site/about/guidelines.xhtml

The REMARK checklist

20	Discuss implications for future research and clinical value.	

For peer review only

Source: McShane LM, Altman DG, Sauerbrei W, Taube SE, Gion M, Clark GM: Reporting recommendations for tumor marker prognostic studies (REMARK). *J Natl Cancer Inst* 2005; 97: 1180-1184.
